# A mathematical framework for the analysis and comparison of contact detection methods for ellipses and ellipsoids

**DOI:** 10.1007/s40571-022-00460-2

**Published:** 2022-02-01

**Authors:** Elham Kheradmand, Marc Laforest, Serge Prudhomme

**Affiliations:** Département de Mathématiques et génie industriel, Polytechnique Montréal, Montréal, QC H3T 1J4 Canada

**Keywords:** Contact detection, Collision detection, Ellipse, Ellipsoid, Discrete element method, Particle methods, Molecular dynamics, Minimization problems

## Abstract

The purpose of this research is to provide a framework for the analysis and comparison of contact detection algorithms for pairs of ellipses and ellipsoids. This work focuses primarily on the category of algorithms that are the most computationally efficient and can produce estimates of the separation and the penetration distance between ellipses and ellipsoids. Specifically, only analytic representations of the ellipses and ellipsoids are considered and contact detection for moving pairs of ellipsoids is not treated. The first contribution is a mathematical framework for the study of these algorithms, most notably with existence and uniqueness proofs for classes of contact detection algorithms, formal descriptions of the asymptotics of pairs of ellipses in close contact (or overlap), and a global analysis of constraints on the normals. The framework highlights the key role played by the different definitions of contact found in the literature, independent of the numerical strategies deployed to estimate the separation/penetration distance. Specifically, it is shown that all the studied algorithms can be expressed as minimization problems, with or without non-binding constraints on the normal(s) at the contact point(s), and that the constraints can be used to identify the global minima among the critical points in the minimization problem. Another contribution of this research, based on the mathematical framework introduced, is a better classification of the known algorithms. These algorithms are compared on established test problems, and their strengths and weaknesses are highlighted and explained in terms of their classification. Furthermore, this research provides comparisons in speed and stability between the most efficient algorithms in each category over a large sample size of test problems. Among the other contributions, this research describes inexpensive but effective initial estimates of the contact to be used in iterative algorithms. Finally, the usefulness of the new framework is illustrated with the introduction of a fast algorithm combining some new and old ideas.

## Introduction

This research describes a unifying framework for the analysis and comparison of contact detection algorithms (CDAs) for pairs of ellipses and ellipsoids^1^. The published CDAs for pairs of ellipses tend to propose incremental improvements over past methods, to offer few comparisons with significantly different algorithms and to rarely distinguish their underlying mathematical problem from their numerical algorithm. In contrast, this detailed study shows that the different definitions of *contact*, usually implicit, play a key role in explaining the numerical behavior of a CDA. A thorough existence and uniqueness theory is presented for each of the different definitions of *contact*, and several new results are presented which formalize intuitive notions, such as the ones of *small overlap* or *near contact*. This new framework leads to a re-classification of the known algorithms into minimization problems, possibly constrained, and a conceptual separation between the definition of contact and the numerical procedure used to characterize that contact.

The framework also includes an experimental methodology to compare different CDAs in terms of accuracy, robustness, and computational cost. Comparing CDAs involves applying them to a set of four illustrative test problems and to a large set of pairs of ellipses with partially known solutions, which are generated by a random but representative sampling from the space of configurations. Finally, this research presents a novel CDA which outperforms previous algorithms in computational time by a factor of at least three, thereby illustrating how these new mathematical notions can lead to significant progress on the numerics of contact detection. In summary, this research sheds new light on an old problem and provides many new directions for future research.

CDAs are an essential component of many complex simulations such as those of granular flows in geo-mechanics, but also of hopper and chute flows in the chemical and food industries, of failure and fracture of brittle materials, and even of the forces of ice flows on offshore structures. In the previous applications, CDAs play a key role in the computation of inter-particle forces and the use of ellipsoids rather than spheres or cylinders and increase the applicability and reliability of the simulations. These numerical applications are often variants of the discrete element method (DEM) [7], or of molecular dynamics simulations (with or without Coulomb forces) [1, 10, 22]. On the other hand, some applications require the same accuracy in the estimation of the separation/penetration distance between ellipses, but do not necessarily need to estimate forces, such as in robotics, computer graphics (CAD/CAM) [14], data uncertainty analysis, computer vision, or virtual reality applications, to name a few. In those cases, ellipses often serve as effective bounding boxes of geometrically complex objects for which collision detection and avoidance is crucial.

Given our intended applications, this study will omit discussing contact detection between particles not defined analytically by an ellipse and will also ignore contact detection for rapidly moving pairs of ellipses. In particular, we are not interested by the contact detection problem when the ellipse is approximated by segments of circles [25, 28, 37, 49], by grid or polar representation of particles [24], by the four arc approximation of ellipses [28, 49], as a polyhedral surface [17], or in the most general form as a combination of NURBS [30, 48]. The analysis is also limited to soft particles, that is to pairs of ellipses for which an accurate estimate of the separation/penetration distance is required, usually to compute forces between particles. This excludes a large set of methods used in static molecular assemblies and based on the Perram–Wertheim theory, and its many successful extensions. Furthermore, when the particles are moving (and rotating) rapidly with respect to their traveling distance, then the techniques of Wang et al. [26, 51] are more appropriate than those studied here. Nonetheless, similarities to these last two techniques are discussed, respectively, in Sects. 3.5 and 3.9. In this respect, this research will focus on highly accurate and computationally effective methods of contact detection for pairs of soft ellipses in the quasi-static regime.

Although the study of granular media should consider particles of almost any shape, it is clear that ellipses are the simplest convex models of particles which can adequately describe both kinetic and rotational energies. In fact, studies have shown that packed assemblies of ellipses exhibit behaviors absent from packed assemblies of disks [8]. Unfortunately, current CDAs for pairs of ellipses can incur a computational overhead that is more than an order of magnitude higher than between pairs of spheres [54], with CDAs between pairs of particles with even more complex geometries requiring at least two orders of magnitude more than spheres. This implies that there are limits to the scale that DEM/MD simulations can attain and of the conclusions that can be reached about the influence of particle geometry on macroscopic soil mechanics. In this sense, faster and more accurate CDAs for pairs of ellipses would help to scale simulations closer to realistic scales. Although it is conjectured that some of the results in this research will extend to hyperquadrics, the contact detection problem for pairs of particles with more general (non-convex) geometries might not fit into a similar framework.

The main idea of the CDA studied here is to find two points associated with the two particles, and from these determine a *contact zone.* One can then use those two points to define a contact point between elliptic particles, a normal direction, and the forces, of equal size but opposite direction. The accuracy and stability of the CDA therefore directly influence the behavior of the short- and long-term dynamics of granular assemblies. We emphasize here that the notion of contact point and of normal vector is not uniquely defined for two overlapping ellipses, in contrast to disks. In fact, as two disjoint ellipses approach each other, their relative velocities and angular motion play a role in determining the evolution of the contact region. In conclusion, our view is that it is more important to propose a well-defined and relevant notion of contact point and separation/penetration distance than to formulate the most general definition which might ultimately be impractical to compute.

The framework developed in this research leads to a natural classification of published CDAs into categories based on their chosen definition of contact, which we now summarize. The framework of Sect. 3 begins by motivating and defining three separate notions of contact, all expressed as minimization problems. The naïve approach for soft ellipses is to identify the points, if any, belonging to the intersection of the two ellipses. This method, referred to as the intersection algorithm (IA) in Sect. 5.1, was analyzed by Rothenburg et al. in [42] but is well known to be unstable and therefore to be avoided. The second most natural definition of contact, in the case when the pair is non-overlapping, begins by identifying the pair of closest points on the ellipses, formalized as the minimum distance pair (MDP) in Lemma 3. When the pair is overlapping, the solution to the minimization problem associated with the MDP is in fact the same as for the IA. For overlapping ellipses, it is therefore more natural to extend the definition of MDP by introducing an additional constraint on the normals, that was previously non-binding for disjoint ellipses. This extended notion of MDP serves as the basis for the closest co-normal algorithm (CCN) of Sect. 5.6, first studied by Wellmann et al. [53]. Unfortunately, this problem is quite difficult to solve since it leads to a large coupled nonlinear system of equations. For that reason, many researchers have developed algorithms that split into two separate smaller problems: for each ellipse, search for the point on that ellipse *closest* to the other ellipse. Typically, closeness is measured with respect to the induced norm of the associated ellipse and leads to the minimum potential pair (MPP) of Lemma 4. This approach serves as the basis to several algorithms [12, 34, 46], which differ only in the solution process but not in the underlying problem definition. This research also examines the common normal algorithm (CNA) of Lin et al. [31]. As far as we know, the only attempt at a survey of these algorithms has been a dedicated chapter in a monograph [32]. Finally, it should be noted that many of the published algorithms have been renamed in this survey because the original names often led to significant misunderstandings about their contact definition (IA, MDP, MPP) and their numerical formulation.

The contact theory of Perram–Wertheim [41, 47] can only be used to detect overlaps but cannot predict separation/penetration distance. This theory only estimates a proxy of contact separation/penetration, not two points on the ellipses, and therefore cannot be used to accurately estimate the force between two ellipses. Hence, we will only indicate some of its similarities to the MDP and the MPP in Sect. 3.5, namely that their *contact potentials* are expressed as minimization problems and require global constraints on the fields of normals associated with the particles. It was first constructed by Vieillard-Baron [47] in 1972 for pairs of ellipses before being generalized by Perram and Wertheim [41] into an effective tool for the computation of thermodynamic properties of dense static assemblies.

The engineering and computer science literature contains several variants of the contact detection problem that will not be studied here, but which nonetheless warrant mention. One of these is the *broad phase search* of contact detection where, for each particle, a preliminary list of neighbors is identified before a *narrow phase search* is completed between the particle and its neighbors, e.g., the type of accurate contact detection we are concerned with. Given the high cost of a CDA for a pair of particles during the narrow phase, an efficient broad phase search can be essential, such as the parallel algorithm over GPUs proposed by NVIDIA [36]. We also remark that there is a large and growing literature on contact detection for swarms of robots and drones [50], with or without centralized control. For the promise of driverless vehicles to become a reality, image recognition and contact detection algorithms will need to become more accurate and reliable.

The mathematical framework for contact detection introduced in this research was deemed necessary in order to unify the current state of the art on the topic, as past contributions were often based on the researchers’ intuition and sometimes lacked precise definitions, formal proofs and exhaustive benchmarks. From a mathematical point of view, this meant it was necessary to distinguish the underlying *contact detection problem*, with its own existence and uniqueness results for its solution, from the numerical *contact detection algorithm*, that is required in order to estimate that solution. The theory presented in Sect. 3 will deal only with the underlying mathematics of the problem, while Sect. 5 will distinguish algorithms based on their underlying contact detection problem and will detail the many different approximations that have been used to solve that problem.

The rigorous framework for CDA introduced in this paper is an attempt to formalize key concepts in contact detection. The first part of the analysis is to motivate the introduction of three separate notions of contact in the form of minimization problems: the intersection set (IS), the minimum distance pair (MDP), and the minimum potential pair (MPP). Existence and uniqueness theorems are given for these notions, which are, rather surprisingly, absent from the literature. In the case of the MPP, it is found that non-binding constraints on the normals exist and these are formalized in the notion of the co-gradient locus of Theorem 6, previously introduced by [46] as the *line of common slope*. The co-gradient locus, being an intrinsically defined object associated with each pair of ellipses, is then used to clarify the notion of two ellipses in *near-perfect contact*. Theorem 7 states that if an intrinsically numerical constraint, as introduced in Definition 6, is satisfied, then the ellipses are guaranteed to be in a configuration consistent with the use case in DEM, namely with at most two points of intersection. As far as the mathematical theory is concerned, not all results have been demonstrated in 3D, but the remaining elements of theory are easily formulated and will be highlighted throughout Sect. 3. Although we have attempted to present a rigorous and self-contained presentation of the framework, many open questions remain and it is our hope that this framework will help to clarify and drive forward future research on this basic question.

The framework goes beyond the theory and contains numerical examples and procedures to compare the different CDA. The comparison between CDAs is particularly difficult because they may have different problem definitions, rely on different numerical algorithms to solve the same intermediate problems, have different implementations, use different tolerance definitions and values, etc. This explains why the literature rarely contains comparisons between more than two CDAs. The approach taken is to generate a large set of pairs of ellipses, appropriately sampled from the space of configurations, to solve the contact detection problem for each CDA, and extract statistical information from the results. Given that we cannot predict beforehand all the configurations of pairs of ellipses which might highlight the weaknesses of different CDAs, it is important to sample the entire space of configurations of pairs of ellipses, notably the ratios of areas, the aspect ratios, the separation/penetration distance, the relative orientations, and the locations of the contact points along the perimeter of the ellipses. The statistical data that are extracted are the mean and variance of the computational time, the accuracy (because the exact MPP is known by construction), and the number of iterations of the underlying nonlinear solvers. These numerical experiments are carried out in Sect. 6, but the detailed algorithm used to generate random pairs of ellipses is detailed in a second paper by the authors [29].

A final contribution of this research is a novel CDA that incorporates some new and old elements and illustrates how the framework presented here can lead one to construct new algorithms. The new CDA, which we have named the *Steered Geometric Potential Algorithm* (S-GPA) for reasons that will become clear later, is an algorithm to compute MPP using the mapping of Sect. 4.2 and Newton’s method. It includes both a novel technique to generate an initial guess of the contact points and a novel constraint ensuring that the algorithm converges iteratively to the unique MPP. Both of these new elements are of independent interest. This new CDA is only briefly explained in Sect. 5.4, but a complete description of the algorithm can be found in [29].

This review paper is organized as follows. We describe in Sect. 2 the mathematical representations of ellipses and ellipsoids. Section 3 introduces a detailed mathematical formulation of the contact detection problem, thereby laying the foundation results for our description of the various techniques in Sect. 5. Section 4.3 briefly reviews efficient initial estimates of the contact point. Afterward, we briefly review the methods and analyze their respective advantages and disadvantages in two- and three-dimensional problems. The last section presents numerical results comparing the accuracy, stability, and cost of the different algorithms.

## Preliminaries on ellipses and ellipsoids

This section sets the stage for the comparison between different contact detection algorithms by collecting often recurring notation and definitions. By beginning with a compact but coherent introduction to the terms and expressions, we hope to make the similarities between the different algorithms quickly transparent.

### Representation of ellipses

An ellipse ${\mathcal {E}}$ is the set of roots ${\varvec{x}}=[x,y]^T \in {\mathbb {R}}^2$ of a quadratic polynomial of the form

1 $$\begin{aligned} f({\varvec{x}}) := ({\varvec{x}}- {\varvec{c}})^T {\mathcal {Q}}({\varvec{x}}- {\varvec{c}}) - 1, \end{aligned}$$

where ${\mathcal {Q}}$ is a symmetric positive-definite (SPD) matrix in ${\mathbb {R}}^{2\times 2}$ and ${\varvec{c}}= [c_x,c_y]^T \in {\mathbb {R}}^2$ is the center of the ellipse. Formally written, an ellipse is defined as

$$\begin{aligned} {\mathcal {E}}= \big \{ {\varvec{x}}\in {\mathbb {R}}^2\,; ({\varvec{x}}- {\varvec{c}})^T {\mathcal {Q}}({\varvec{x}}- {\varvec{c}}) - 1 =0 \big \}. \end{aligned}$$

**Fig. 1:**
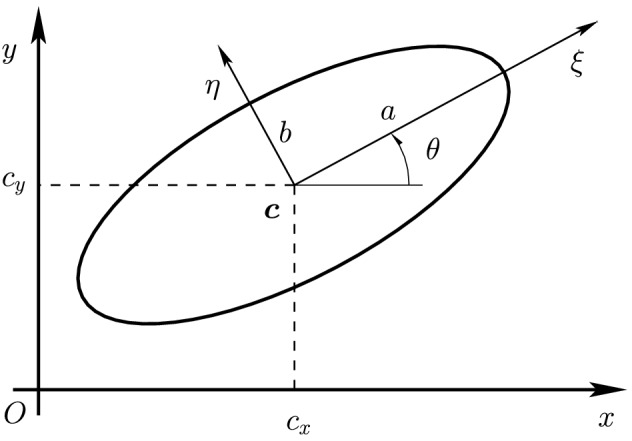
Ellipse in global coordinate system (*O*, *x*, *y*) with local coordinate system $(O,\xi ,\eta )$ centered at ${\varvec{c}}$

The coordinates ${\varvec{x}}$ in which an ellipse is initially given will be referred to as the *global coordinates*, but there exists an isometry to a system of coordinates in which the geometry of ${\mathcal {E}}$ is especially straightforward. Indeed, a fundamental result of linear algebra states that for each SPD matrix ${\mathcal {Q}}$, there exists an orthogonal matrix ${\mathcal {R}}$, that is satisfying ${\mathcal {R}}^{-1} = {\mathcal {R}}^T$ and therefore in the form

2 $$\begin{aligned} {\mathcal {R}}:= \begin{bmatrix} \cos \theta &{} - \sin \theta \\ \sin \theta &{} ~ \cos \theta \end{bmatrix}, \qquad \theta \in [-\pi , \pi [, \end{aligned}$$

such that the matrix

3 $$\begin{aligned} {\mathcal {D}}:= {\mathcal {R}}^T {\mathcal {Q}}{\mathcal {R}}= \begin{bmatrix} 1/a^2 &{} 0 \\ 0 &{} 1/b^2 \end{bmatrix}, \end{aligned}$$

is diagonal with strictly positive entries, i.e., the eigenvalues of ${\mathcal {Q}}$. An example of an ellipse is shown in Fig. 1 under the assumption that $a \ge b$. The axes corresponding to *a* and *b* are called the *semimajor axis* and the *semiminor axis*, respectively. Accordingly, *a* and *b* are usually referred to as the *semi-axes* of the ellipse. Further including a translation to send the center ${\varvec{c}}$ to the origin, we can introduce the *local coordinates* $\varvec{\xi }= [\xi ,\eta ]^T$,

4 $$\begin{aligned} \varvec{\xi }= {\mathcal {R}}^T ({\varvec{x}}- {\varvec{c}}), \end{aligned}$$

with respect to which the ellipse consists in the set of roots of

5 $$\begin{aligned} {\widehat{f}}(\varvec{\xi }) = \varvec{\xi }^T {\mathcal {D}}\varvec{\xi }- 1, \end{aligned}$$

which can be recast in the classical form

6 $$\begin{aligned} {\widehat{f}}(\xi ,\eta ) = \frac{\xi ^2}{a^2} + \frac{\eta ^2}{b^2} - 1. \end{aligned}$$

In this paper, *f* will be called the *global geometric potential*, or simply potential, of the ellipse, while ${\widehat{f}}$ will be called the *local potential*. Clearly, the potential will always be a convex function with a minimum at the center ${\varvec{c}}$ of the ellipse.

#### Definition 1

(${\mathcal {Q}}$*-norm)* A SPD matrix ${\mathcal {Q}}$ induces the so-called ${\mathcal {Q}}$-norm

7 $$\begin{aligned} \Vert {\varvec{x}}\Vert _{{\mathcal {Q}}} := \sqrt{{\varvec{x}}^T {\mathcal {Q}}{\varvec{x}}}, \quad \forall {\varvec{x}}\in {\mathbb {R}}^2. \end{aligned}$$

This definition allows one to interpret an ellipse ${\mathcal {E}}$ as the “circle” satisfying $\Vert {\varvec{x}}- {\varvec{c}}\Vert _{{\mathcal {Q}}}^2 = 1$ in the global coordinates. Eventually, when we consider the contact problem for two ellipses ${\mathcal {E}}_i$ and ${\mathcal {E}}_j$, we may replace the subscript ${\mathcal {Q}}$ by the index *i* of the ellipse ${\mathcal {E}}_i$. Throughout this paper, the norm $\Vert \cdot \Vert$ written without a subscript will denote the usual Euclidean norm.

We now proceed with an explicit description of an ellipse as defined by (1). Let the matrix ${\mathcal {Q}}$ be given by

8 $$\begin{aligned} {\mathcal {Q}}= \begin{bmatrix} A &{} C \\ C &{} B \end{bmatrix}, \end{aligned}$$

where positive-definiteness is ensured by the conditions $A>0$, $B>0$, and ${\text {det}} {\mathcal {Q}}= AB - C^2 > 0$. Then, in the global coordinates, the potential (1) is given as

9 $$\begin{aligned} f(x,y)&= A(x - c_x)^2 + B(y - c_y)^2 \nonumber \\&\quad + 2C(x - c_x)(y - c_y) - 1. \end{aligned}$$

For convenience, we provide below the explicit relationships between ${\mathcal {D}}$ and ${\mathcal {R}}$, and ${\mathcal {Q}}$, namely

10 $$\begin{aligned} \begin{aligned}&A = \frac{\cos ^2\theta }{a^2} + \frac{\sin ^2\theta }{b^2}, \\&B = \frac{\sin ^2\theta }{a^2} + \frac{\cos ^2\theta }{b^2}, \\&C = \sin \theta \cos \theta \left( \frac{1}{{a}^2} - \frac{ 1}{{b}^2}\right) . \end{aligned} \end{aligned}$$

An alternative form in which to express the potential is based on separating out the quadratic, linear, and constant terms. Starting from (1), we find that the points ${\varvec{x}}$ on an ellipse satisfy

11 $$\begin{aligned} \begin{aligned} f({\varvec{x}})&= ({\varvec{x}}- {\varvec{c}})^T {\mathcal {Q}} ({\varvec{x}}- {\varvec{c}}) - 1\\&= {\varvec{x}}^T {\mathcal {Q}} {\varvec{x}}- {\varvec{x}}^T {\mathcal {Q}} {\varvec{c}}- {\varvec{c}}^T {\mathcal {Q}} {\varvec{x}}+ {\varvec{c}}^T {\mathcal {Q}} {\varvec{c}}- 1\\&= {\varvec{x}}^T {\mathcal {Q}} {\varvec{x}}- {\varvec{x}}^T ({\mathcal {Q}} {\varvec{c}}) - ({\mathcal {Q}} {\varvec{c}})^T {\varvec{x}}+ {\varvec{c}}^T {\mathcal {Q}} {\varvec{c}}- 1. \end{aligned} \end{aligned}$$

Introducing

12 $$\begin{aligned} F&= {\varvec{c}}^T {\mathcal {Q}} {\varvec{c}}-1, \quad \begin{bmatrix} D \\ E \end{bmatrix} = - {\mathcal {Q}} {\varvec{c}}, \quad {\mathcal {P}} = \begin{bmatrix} A &{} C &{} D \\ C &{} B &{} E \\ D &{} E &{} F \end{bmatrix}, \quad \nonumber \\ {\varvec{z}}&= \begin{bmatrix} x \\ y \\ 1 \end{bmatrix}, \end{aligned}$$

the potential *f* can then be rewritten in augmented matrix form as

13 $$\begin{aligned} f(x,y)&= {\varvec{z}}^T {\mathcal {P}} {\varvec{z}}\nonumber \\&= Ax^2+By^2+2Cxy+2Dx+2Ey+F. \end{aligned}$$

Another useful description is based on the parameterization of the ellipse in terms of a parameter $t \in [-\pi , \pi [$ such that all points given by

14 $$\begin{aligned} \varvec{\xi }(t) = \begin{bmatrix} \xi (t) \\ \eta (t) \end{bmatrix} = {\mathcal {D}}^{-1/2} \begin{bmatrix} \cos t \\ \sin t \end{bmatrix} = \begin{bmatrix} a \cos t \\ b \sin t \end{bmatrix} \end{aligned}$$

lie on the ellipse. Using the mapping (4), the ellipse in the global coordinate system consists then of the points

15 $$\begin{aligned} {\varvec{x}}(t) = {\mathcal {R}}\varvec{\xi }(t) + {\varvec{c}}= {\mathcal {R}}{\mathcal {D}}^{-1/2} \begin{bmatrix} \cos t \\ \sin t \end{bmatrix} + {\varvec{c}}, \qquad t \in [-\pi ,\pi [.\nonumber \\ \end{aligned}$$

Certain algorithms for contact detection between two ellipses require the outward unit normal vector at a point ${\varvec{x}}$ or, equivalently, at a point $\varvec{\xi }$, on an ellipse. From the definitions of the potentials *f* and ${\widehat{f}}$, see Eqs. (1) and (5), respectively, a simple calculation shows that the normal is given in global coordinates as

16 $$\begin{aligned} {\varvec{n}}({\varvec{x}}) = \frac{\nabla f ({\varvec{x}}) }{\Vert \nabla f ({\varvec{x}}) \Vert } = \frac{ {\mathcal {Q}}({\varvec{x}}- {\varvec{c}}) }{\Vert {\mathcal {Q}}({\varvec{x}}- {\varvec{c}}) \Vert }, \end{aligned}$$

or in local coordinates as

17 $$\begin{aligned} {\varvec{n}}(\varvec{\xi }) = \frac{\nabla {\widehat{f}} (\varvec{\xi }) }{\Vert \nabla {\widehat{f}} (\varvec{\xi }) \Vert } = \frac{ {\mathcal {D}} \varvec{\xi }}{\Vert {\mathcal {D}} \varvec{\xi }\Vert } \, . \end{aligned}$$

Without delving into the explicit calculations, which can be found in several references [4, 5], we note that the minimum radius of curvature is given by

18 $$\begin{aligned} {\underline{\rho }} = \frac{b^2}{a}. \end{aligned}$$

There exist several alternative descriptions of ellipses. For the sake of completeness, we mention here some of the most important descriptions even if they are not used in this review. The first one will nevertheless motivate an algorithm for finding initial guess points, to be detailed in Sect. 4.3. We recall that the focal points of an ellipse, ${\varvec{f}}_1$ and ${\varvec{f}}_2$, are located on its semimajor axis, at equal distance from the center, and are explicitly given in local coordinates as

19 $$\begin{aligned} {\varvec{f}}_1 = \big (-\sqrt{a^2-b^2},0 \big ), \qquad {\varvec{f}}_2 = \big ( + \sqrt{a^2-b^2},0 \big ). \end{aligned}$$

The ellipse can then be defined as the set of points ${\varvec{x}}$ that satisfy

20 $$\begin{aligned} \Vert {\varvec{x}}- {\varvec{f}}_1 \Vert + \Vert {\varvec{x}}- {\varvec{f}}_2 \Vert = 2a. \end{aligned}$$

Moreover, it is possible to show that the normal vector at ${\varvec{x}}$ generates a line that bisects the angle $\angle {\varvec{f}}_1 {\varvec{x}}{\varvec{f}}_2$. There is also a well-known description of an ellipse in terms of its eccentricity $e = \sqrt{a^2 - b^2}/a \in [0,1]$, with $e = 0$ corresponding to a circle [18]. Ellipses can be geometrically obtained as the cross section of the intersection of an inclined plane with a conic section, but this description of a 2-D ellipse requires three dimensions, thus making it less practical. Ellipses can also be described using mechanical means, such as the Trammel of Archimedes, the Tusi couple, or the ellipsograph of Benjamin Bramer [2, 11, 18]. The Steiner method for the construction of an ellipse is quite elegant but requires a discretization and is therefore not relevant to the continuous contact detection problem. In summary, ellipses possess a wealth of fascinating and unexpected properties, but none of these seem to be as useful as (1) or (5) when one needs to numerically estimate contact points.

### Representation of ellipsoids

Similarly to ellipses, an ellipsoid ${\mathcal {E}}\subset {\mathbb {R}}^3$ is the set of roots to the potential:

21 $$\begin{aligned} f({\varvec{x}}) := ({\varvec{x}}- {\varvec{c}})^T {\mathcal {Q}}({\varvec{x}}- {\varvec{c}}) - 1 \end{aligned}$$

where ${\mathcal {Q}}$ is a $3\times 3$ SPD matrix and ${\varvec{c}}\in {\mathbb {R}}^3$ is the center of the ellipsoid. As in 2-D, there exists an orthogonal change of variable ${\mathcal {R}}\in {\mathbb {R}}^{3\times 3}$, ${\mathcal {R}}^{-1} = {\mathcal {R}}^T$, which diagonalizes ${\mathcal {Q}}$ such that ${\mathcal {D}}= {\mathcal {R}}^T {\mathcal {Q}}{\mathcal {R}}$. The eigenvalues of ${\mathcal {Q}}$, i.e., the entries of ${\mathcal {D}}$, are all strictly positive and are denoted by $a^{-2}$, $b^{-2}$, and $c^{-2}$, where the positive constants *a*, *b*, and *c* are assumed to be ordered as $c \le b \le a$. Using the change of variable (4), with $\varvec{\xi }= [\xi ,\eta ,\zeta ]^T$, one can write the local potential in its so-called local coordinate system $(O,\xi ,\eta ,\zeta )$ as

$$\begin{aligned} {\widehat{f}}(\varvec{\xi }) = \varvec{\xi }^T {\mathcal {D}}\varvec{\xi }- 1, \end{aligned}$$

or

22 $$\begin{aligned} {\widehat{f}}(\xi ,\eta ,\zeta ) = \frac{\xi ^2}{a^2} + \frac{\eta ^2}{b^2} + \frac{\zeta ^2}{c^2} - 1. \end{aligned}$$

The positive constants *a*, *b*, and *c* are called the semi-axes of the ellipsoid.

#### Remark 1

Unlike in 2-D, the explicit form of the rotation matrix ${\mathcal {R}}$ can be obtained in several manners. We first note that an arbitrary ellipsoid is defined in terms of nine parameters: the coordinates of its center ${\varvec{c}}= [c_x,c_y,c_z]^T$ and the six entries of the symmetric matrix ${\mathcal {Q}}$. However, since the matrix ${\mathcal {Q}}$ can also be written as ${\mathcal {R}}{\mathcal {D}}{\mathcal {R}}^T$, these six entries can also be identified with the three positive eigenvalues appearing as the diagonal elements of ${\mathcal {D}}$, and the three parameters needed to describe a general orthogonal transformation ${\mathcal {R}}$. In other words, one needs to introduce three angles, each corresponding to an elementary rotation, and write ${\mathcal {R}}$ as the composition of the three rotation matrices in order to map the local coordinate system into the principal axes of the ellipsoid in the global coordinate system. The choice of the three angles is actually not unique and depends on the representation considered, for example the Euler rotations [19] or the quaternion rotations [21]. We will not describe these methods here and will simply assume that ${\mathcal {R}}$, if necessary, is provided using one of the methods as

$$\begin{aligned} {\mathcal {R}}= \begin{bmatrix} r_{11} &{} r_{12} &{} r_{13} \\ r_{21} &{} r_{22} &{} r_{23} \\ r_{31} &{} r_{32} &{} r_{33} \end{bmatrix}. \end{aligned}$$

An ellipsoid in the local coordinate system can be represented in terms of the spherical coordinates $(u,v) \in [-\pi ,\pi [ \times [0,\pi [$ as

23 $$\begin{aligned} \varvec{\xi }(u,v)= & {} D^{-1/2} \begin{bmatrix} \cos u \sin v \\ \sin u \sin v \\ \cos v \end{bmatrix} = \begin{bmatrix} a &{} 0 &{} 0 \\ 0 &{} b &{} 0 \\ 0 &{} 0 &{} c \end{bmatrix} \begin{bmatrix} \cos u \sin v \\ \sin u \sin v \\ \cos v \end{bmatrix}\nonumber \\= & {} \begin{bmatrix} a \cos u \sin v \\ b \sin u \sin v \\ c \cos v \end{bmatrix}. \end{aligned}$$

Using the mapping ${\varvec{x}}= {\mathcal {R}}\varvec{\xi }+ {\varvec{c}}$, the ellipsoid in the global coordinates system is therefore parameterized as

24 $$\begin{aligned} {\varvec{x}}(u,v)= & {} {\mathcal {R}}\varvec{\xi }(u,v) + {\varvec{c}}= {\mathcal {R}}{\mathcal {D}}^{-1/2} \begin{bmatrix} \cos u \sin v \\ \sin u \sin v \\ \cos v \end{bmatrix} + \begin{bmatrix} c_x\\ c_y \\ c_z \end{bmatrix},\nonumber \\&\quad \forall (u,v) \in [-\pi ,\pi [ \times [0,\pi [. \end{aligned}$$

As in 2-D, the outward unit normal vector at a point ${\varvec{x}}$ on an ellipsoid is given in global coordinates by

25 $$\begin{aligned} {\varvec{n}}({\varvec{x}}) = \frac{\nabla f ({\varvec{x}}) }{\Vert \nabla f ({\varvec{x}}) \Vert } = \frac{ {\mathcal {Q}}({\varvec{x}}- {\varvec{c}}) }{\Vert {\mathcal {Q}}({\varvec{x}}- {\varvec{c}}) \Vert }, \end{aligned}$$

or in local coordinates by

26 $$\begin{aligned} {\varvec{n}}(\varvec{\xi }) = \frac{\nabla {\widehat{f}} (\varvec{\xi }) }{\Vert \nabla {\widehat{f}} (\varvec{\xi }) \Vert } = \frac{ {\mathcal {D}} \varvec{\xi }}{\Vert {\mathcal {D}} \varvec{\xi }\Vert } \, . \end{aligned}$$

We conclude by observing that the gradient in 3-D is given by the same formula as (18), while the minimum radius of curvature is, assuming $a \ge b \ge c$,

27 $$\begin{aligned} {\underline{\rho }} = \frac{c^2}{a}. \end{aligned}$$

### Family of concentric similar ellipses and ellipsoids

Let ${\mathcal {E}}$ be an arbitrary ellipse or ellipsoid with potential

$$\begin{aligned} f({\varvec{x}}) = ({\varvec{x}}-{\varvec{c}})^T {\mathcal {Q}}({\varvec{x}}-{\varvec{c}}) - 1. \end{aligned}$$

Then, the family of concentric similar ellipses ($$d=2)$$ and ellipsoids ($$d=3$$) associated with $${\mathcal {E}}$$ consists of the sets

28 $$\begin{aligned} {\mathcal {E}}(r) := \big \{ {\varvec{x}}\in {\mathbb {R}}^d;\ f({\varvec{x}}) - (r^2-1) = 0 \big \}, \qquad \forall r \ge 0, \end{aligned}$$

or as the roots of $f_r({\varvec{x}}):= f({\varvec{x}})-(r^2-1)=({\varvec{x}}-{\varvec{x}})^T{\mathcal {Q}}({\varvec{x}}-{\varvec{x}})-r^2$. We note that two ellipses or two ellipsoids within the same family, i.e., ${\mathcal {E}}(r_1)$ and ${\mathcal {E}}(r_2)$ with $r_1 \ne r_2$, form a homoeoid, that is, the bounded region between ${\mathcal {E}}(r_1)$ and ${\mathcal {E}}(r_2)$. Moreover, ${\mathcal {E}}(0)$ reduces to the singleton $\{ {\varvec{c}}\}$, while ${\mathcal {E}}(1)$ corresponds to ${\mathcal {E}}$. Furthermore, for every point ${\varvec{x}}\in {\mathbb {R}}^d$, there exists a unique $r\ge 0$ such that ${\varvec{x}}\in {\mathcal {E}}(r)$ and the gradient of $f_r$ at ${\varvec{x}}\in {\mathcal {E}}(r)$ is given by

$$\begin{aligned} \nabla f_r({\varvec{x}}) = \nabla f ({\varvec{x}}) = 2 {\mathcal {Q}}({\varvec{x}}-{\varvec{c}}). \end{aligned}$$

In other words, the outward unit normal vector to the ellipse/ellipsoid ${\mathcal {E}}(r)$ associated with ${\mathcal {E}}$ at an arbitrary point ${\varvec{x}}\in {\mathbb {R}}^d \backslash \{{\varvec{c}}\}$ is given by

29 $$\begin{aligned} {\varvec{n}}({\varvec{x}}) = \frac{\nabla f_r({\varvec{x}})}{\Vert \nabla f_r({\varvec{x}}) \Vert } = \frac{\nabla f ({\varvec{x}})}{\Vert \nabla f ({\varvec{x}}) \Vert } = \frac{{\mathcal {Q}}({\varvec{x}}-{\varvec{c}})}{\Vert {\mathcal {Q}}({\varvec{x}}-{\varvec{c}}) \Vert }. \end{aligned}$$

We now provide some properties satisfied by the vector field ${\varvec{n}}({\varvec{x}})$ associated with an ellipse ${\mathcal {E}}$, which will be extensively used in Sect. 3. These properties will be expressed using complex multiplication and elements of projective geometry which we now recall. Let $S^1$ be the set of points of unit modulus in the complex plane ${\mathbb {C}}$, which will be used to represent both the unit gradient field ${\varvec{n}}$ and unit direction ${\varvec{w}}$. Given the points $e^{i\omega }$ and $e^{i\theta }$ in $S^1$, complex multiplication between the two points can be written as

$$\begin{aligned} e^{i\omega } e^{i\theta } = e^{i (\omega +\theta )}, \end{aligned}$$

thereby representing the composition of two rotations.

In projective geometry, the real plane ${\mathbb {R}}^2$ is imbedded into the compact space of all directions in ${\mathbb {R}}^3$ using the association of $[x,y]^T \in {\mathbb {R}}^3$ with the direction

$$\begin{aligned}{}[x:y:1] := \{ [xt,yt,t]^T \in {\mathbb {R}}^3 \, | \, t\in {\mathbb {R}}^+ \}, \end{aligned}$$

identified here in *homogeneous coordinates*. The space of all directions

$$\begin{aligned}{}[x:y:z] := \{ [xt,yt,zt]^T \in {\mathbb {R}}^3 {\setminus }\{ {\mathbf {0}} \} \, | \, t\in {\mathbb {R}}^+ \}, \end{aligned}$$

is called the *projective sphere* $\text { SP}^{\,2}$, not to be confused with the projective plane obtained after associating antipodal points in the projective sphere. The *points at infinity* are those corresponding to the directions [*x* : *y* : 0], thus forming a circle. In fact, given an affine map $g: {\mathbb {R}}^2 \rightarrow {\mathbb {R}}^2$, say

$$\begin{aligned} g({\varvec{x}}) = {\mathcal {T}} {\varvec{x}}+ {\mathbf {b}} , \end{aligned}$$

for ${\mathbf {b}} \in {\mathbb {R}}^2$ and ${\mathcal {T}}$ a $2 \times 2$ matrix, then along the segment in direction ${\varvec{w}}\in S^1$

$$\begin{aligned} {\varvec{x}}= {\varvec{c}}+ t {\varvec{w}}\, , \quad t \in {\mathbb {R}}^+ , \end{aligned}$$

we can define a limiting direction

$$\begin{aligned} \lim _{t \rightarrow \infty } \frac{1}{t} g({\varvec{x}}) = \lim _{t \rightarrow \infty } \frac{1}{t} g({\varvec{c}}+ t {\varvec{w}}) = \lim _{t \rightarrow \infty } {\mathcal {T}} {\varvec{w}}+ \frac{1}{t} {\mathcal {T}} {\varvec{c}}= {\mathcal {T}} {\varvec{w}}. \end{aligned}$$

This association is independent of ${\varvec{c}}$ and parameterization *t*, thus leading to a well-defined map $g^\infty : S^1 \rightarrow S^1$ according to

$$\begin{aligned} {\varvec{w}}\longmapsto \frac{{\mathcal {T}} {\varvec{w}}}{\Vert {\mathcal {T}} {\varvec{w}}\Vert } . \end{aligned}$$

This map is the restriction at infinity of the extension of *g* from the projective sphere to itself.

#### Lemma 1

Consider an ellipse ${\mathcal {E}}\subset {\mathbb {R}}^2$ centered at ${\varvec{c}}\in {\mathbb {R}}^2$ whose unit vectors associated with the semimajor and semiminor axes are $\varvec{\xi }$ and $\varvec{\eta }$, respectively, oriented counterclockwise. The vector field ${\varvec{n}}$ given by (29) satisfies the following properties:

(i) The vector field ${\varvec{n}}({\varvec{x}})$ is well defined $\forall {\varvec{x}}\in {\mathbb {R}}^2 {\setminus } \{ {\varvec{c}}\}$.
(ii) The relations ${\varvec{n}}({\varvec{c}}\pm t \varvec{\xi }) = \pm \varvec{\xi }$ and ${\varvec{n}}({\varvec{c}}\pm t \varvec{\eta }) = \pm \varvec{\eta }$ hold $\forall t \in {\mathbb {R}}^+$.
(iii) Given ${\varvec{w}}\in S^1$, ${\varvec{n}}( {\varvec{c}}+ t {\varvec{w}})$ is constant $\forall t \in {\mathbb {R}}^+$.
(iv) The map
  30 $$\begin{aligned} \begin{aligned} {\mathcal {N}}: S^1&\longrightarrow S^1 \\ {\varvec{w}}&\longmapsto \lim _{r \rightarrow \infty } {\varvec{n}}({\varvec{c}}+ r {\varvec{w}}) , \end{aligned} \end{aligned}$$
  is well defined and satisfies the following properties.
  - $\pm \varvec{\xi }$ and $\pm \varvec{\eta }$ are fixed points.
  - ${\mathcal {N}}$ is bijective and ${\mathcal {N}}$ is the identity if and only if ${\mathcal {E}}$ is a circle.
  - If ${\varvec{w}}=e^{i\sigma } \varvec{\xi }\in S^1$, $\sigma \in [0,2\pi [$, there exists $\theta \in \ ] -\pi /2,\pi /2[$ such that
    31 $$\begin{aligned} {\mathcal {N}}({\varvec{w}})&= e^{i \theta } {\varvec{w}}=e^{i (\theta +\sigma )} \varvec{\xi }, \quad \text {with} \quad \tan (\theta + \sigma ) \nonumber \\&= (a/b)^2 \tan \sigma . \end{aligned}$$
  - If ${\varvec{x}}_0 \ne {\varvec{c}}$, there exists $R= R({\varvec{x}}_0) \in {\mathbb {R}}^+$, such that for $r \ge R$ the estimate
    32 $$\begin{aligned}&\big \Vert {\mathcal {N}}({\varvec{w}}) - {\varvec{n}}({\varvec{x}}_0 + r{\varvec{w}}) \big \Vert \nonumber \\&\quad = {\mathcal {O}}\big (r^{-1}\Vert {\varvec{x}}_0 - {\varvec{c}}\Vert \big ), \end{aligned}$$
    is uniform with respect to ${\varvec{w}}\in S^1$.

**Fig. 2:**
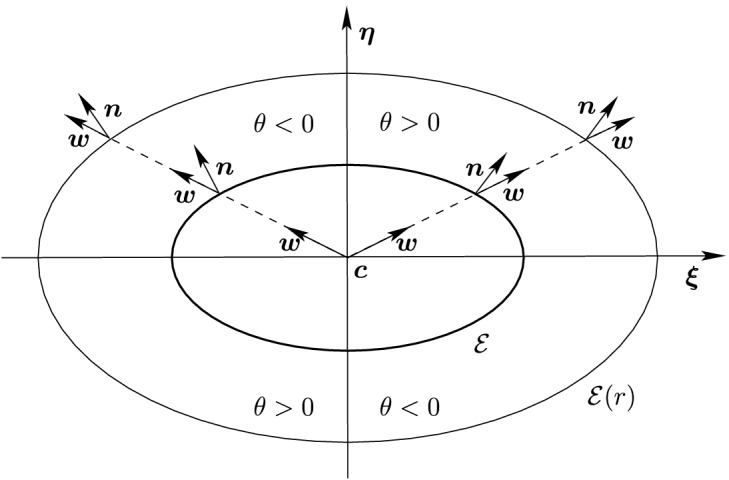
Illustration of the Property (iii) of Lemma 1, showing that the vector ${\varvec{n}}({\varvec{c}}+t{\varvec{w}})$ is constant for a given vector ${\varvec{w}}$ and $t>0$. The angle $\theta$ is the angle between ${\varvec{n}}$ and ${\varvec{w}}$ measured counterclockwise

#### Proof

From (29), the vector field ${\varvec{n}}({\varvec{x}})$ is the unit vector field associated with the gradient field

33 $$\begin{aligned} \nabla f({\varvec{x}}) = 2 {\mathcal {Q}}({\varvec{x}}-{\varvec{c}}). \end{aligned}$$

Given that ${\mathcal {Q}}$ is SPD, $\nabla f$ only vanishes at ${\varvec{x}}= {\varvec{c}}$. This proves Property (i). To demonstrate Property (ii), we observe that $\varvec{\xi }$ and $\varvec{\eta }$ are the eigenvectors of ${\mathcal {Q}}$ associated with the eigenvalues $1/a^2$ and $1/b^2$, respectively; see (3). Hence, substituting ${\varvec{x}}= {\varvec{c}}\pm t \varvec{\xi }$ into (33), we find

$$\begin{aligned} 2 {\mathcal {Q}}({\varvec{x}}- {\varvec{c}}) = 2 {\mathcal {Q}}( \pm t \varvec{\xi }) = \pm \frac{2t}{a^2} \varvec{\xi }, \end{aligned}$$

which implies that ${\varvec{n}}({\varvec{x}}) = \pm \varvec{\xi }$. Similarly, substituting ${\varvec{x}}= {\varvec{c}}\pm t \varvec{\eta }$ into (33) shows that ${\varvec{n}}({\varvec{x}}) = \pm \varvec{\eta }$. Let ${\varvec{w}}\in S^1$ and consider the half-line supported by ${\varvec{w}}$, i.e., the set of points ${\varvec{x}}= {\varvec{c}}+ t {\varvec{w}}$ with $t >0$. The gradients along the half-line

34 $$\begin{aligned} \nabla f({\varvec{x}}) = 2t {\mathcal {Q}}{\varvec{w}}\end{aligned}$$

are all positive multiples of the same vector ${\mathcal {Q}}{\varvec{w}}$. Hence, the vector field ${\varvec{n}}({\varvec{x}})$ is constant along the half-line, which proves Property (iii). This is illustrated in Fig. 2.

We now consider the proof of (iv) by first demonstrating (d). The bound (32) implies that the function

$$\begin{aligned} {\mathcal {N}}({\varvec{w}}) = \lim _{r \rightarrow \infty } {\varvec{n}}( {\varvec{c}}+ r {\varvec{w}}) \end{aligned}$$

is in fact the same as if we had taken $\lim {\varvec{n}}({\varvec{x}}_0 + r {\varvec{w}})$ and therefore does not depend on the origin ${\varvec{x}}_0$ of the segment ${\varvec{x}}_0+r {\varvec{w}}$. For any ${\varvec{x}}_0 \ne {\varvec{c}}$ and any direction ${\varvec{w}}$,

$$\begin{aligned} \nabla f({\varvec{x}}_0+r {\varvec{w}})&= 2 {\mathcal {Q}}\big (r {\varvec{w}}- ({\varvec{c}}-{\varvec{x}}_0)\big )\\&= 2r {\mathcal {Q}}{\varvec{w}}- 2 {\mathcal {Q}}({\varvec{c}}-{\varvec{x}}_0). \end{aligned}$$

For large and positive *r*, we have that

$$\begin{aligned} {\varvec{n}}( {\varvec{x}}_0+ r {\varvec{w}})= & {} \frac{{\mathcal {Q}}{\varvec{w}}- r^{-1} {\mathcal {Q}}({\varvec{c}}-{\varvec{x}}_0)}{\Vert {\mathcal {Q}}{\varvec{w}}-r ^{-1} {\mathcal {Q}}({\varvec{c}}-{\varvec{x}}_0) \Vert } = \frac{{\mathcal {Q}}{\varvec{w}}}{\Vert {\mathcal {Q}}{\varvec{w}}\Vert } \\&\quad +\, {\mathcal {O}}\big (r^{-1}\Vert {\varvec{x}}_0 - {\varvec{c}}\Vert \big ) \approx {\mathcal {N}}( {\varvec{w}}). \end{aligned}$$

As a matter of fact, this approximation can be made uniform in ${\varvec{w}}$ for *r* sufficiently large. In other words, there exist constants *R*, $\delta$, and *C* such that $\forall r \ge R$ and $\forall {\varvec{x}}_0 \in {\mathbb {R}}^2$ satisfying $\Vert {\varvec{x}}_0 - {\varvec{c}}\Vert < \delta$, one has

$$\begin{aligned} \big \Vert {\mathcal {N}}( {\varvec{w}}) - {\varvec{n}}({\varvec{x}}_0+ r {\varvec{w}}) \big \Vert < C \frac{\Vert {\varvec{x}}_0 - {\varvec{c}}\Vert }{r}, \qquad \forall {\varvec{w}}\in S^1. \end{aligned}$$

Property (iv)–(a) follows immediately from ii), while Property (iv)–(b) follows from (iv)–(c). In particular, we observe that ${\mathcal {N}}({\varvec{w}})$ is the identity if and only if $\theta = 0$, which, according to relation (31), occurs if and only if $a/b=1$.

Only Property (iv)–(c) still needs to be demonstrated. We shall begin by proving it for ${\varvec{w}}\in [\varvec{\xi },\varvec{\eta }] \subset S^1$. Consider the parameterization by $\sigma \in [0,\pi /2]$ of every direction ${\varvec{w}}( \sigma ) \in [\varvec{\xi },\varvec{\eta }]$ according to

35 $$\begin{aligned} \sigma \longmapsto {\varvec{w}}(\sigma ) := \cos \sigma \, \varvec{\xi }+ \sin \sigma \, \varvec{\eta }, \end{aligned}$$

and remark that

$$\begin{aligned} \nabla f \big ({\varvec{c}}+ r {\varvec{w}}(\sigma )\big ) = 2r {\mathcal {Q}} {\varvec{w}}(\sigma ) = 2r \bigg [ \frac{\cos \sigma }{a^2} \, \varvec{\xi }+ \frac{\sin \sigma }{b^2}\, \varvec{\eta }\bigg ] . \end{aligned}$$

From this expression, it is clear that ${\mathcal {N}}\big ({\varvec{w}}(\sigma )\big )$ belongs to $[\varvec{\xi },\varvec{\eta }] \subset S^1$. Hence, there exists an angle ${\widehat{\sigma }} \in [0,\pi /2]$ such that

$$\begin{aligned} {\mathcal {N}}\big ( {\varvec{w}}(\sigma ) \big ) = {\varvec{w}}({\widehat{\sigma }}). \end{aligned}$$

In fact, for all $\sigma \in [0,\pi /2[$ and ${\widehat{\sigma }} \in [0,\pi /2[$, we have the relation

36 $$\begin{aligned} \tan {\widehat{\sigma }} = \frac{a^2}{b^2} \tan \sigma \end{aligned}$$

announced in (31). We remark that the derivative of the map (30) in the coordinates (35) satisfies

37 $$\begin{aligned} \frac{d {\widehat{\sigma }}}{d \sigma } = \frac{a^2}{b^2} \frac{\cos ^2 {\widehat{\sigma }}}{\cos ^2 \sigma } > 0. \end{aligned}$$

This shows that the map is bijective over $[\varvec{\xi },\varvec{\eta }]$ and that the map is the identity if and only if the ellipse is a circle (i.e., $b=a$). In the map (30), the angle $\theta$ is given by

$$\begin{aligned} \theta = {\widehat{\sigma }} - \sigma , \end{aligned}$$

and because ${\widehat{\sigma }} > \sigma$ by (36) while both angles belong to $[0,\pi /2[$, then $\theta \in [0,\pi /2[$. Since ${\mathcal {N}}$ has a fixed point at ${\varvec{w}}= \varvec{\eta }$, that is when $\sigma = \pi /2$, we conclude that $\theta (\pi /2)=0$. Therefore, for all $\sigma \in [0,\pi /2]$, we have $\theta (\sigma ) \in [0,\pi /2[$. In fact, the parameterization (35) with $\sigma \in [-\pi /2,0]$ can be used for ${\varvec{w}}(\sigma ) \in [-\varvec{\eta },\varvec{\xi }] \subset S^1$ and leads again to the relation (36). Applying the same argument (or by symmetry along the $\varvec{\eta }$ axis) over $[\varvec{\eta },-\varvec{\xi }]$ and $[-\varvec{\xi },-\varvec{\eta }]$ demonstrates (iv)–(c). In all these cases, we have that $\theta \in \ ]-\pi /2,\pi /2[$. This concludes the proof. $\square$

## Contact detection problems

The purpose of this section is to introduce elementary notions of *contact points* and of their properties for pairs of ellipses. Lacking a common framework, much of the past work provided little indication of the connections between the different algorithms. This section is an attempt at filling this void by presenting a few precise definitions and results which will serve as a common thread in later comparisons of the different contact detection algorithms. The first element to comparing *contact detection algorithms* is to identify the mathematical *contact detection problems* which they are attempting to solve. In numerical analysis, this requires the demonstration of the existence of a unique solution to the contact detection problem, and often some stability bounds. This will be established for the three contact detection problems we have identified in the literature, but stability bounds and accuracy estimates for contact detection algorithms remain open. Complete proofs will be provided in ${\mathbb {R}}^2$ as some gaps in the theory remain in ${\mathbb {R}}^3$.

The *Potential Contact Theory* of Perram and Wertheim [40, 41], based on the earlier work of Veillard-Baron [47], is formulated in algebraic terms that makes it one of the most mathematically complete theories to date. We will not be examining in detail the Perram–Wertheim theory because it does not provide estimates of separation/penetration distance [9], although it is widely used in static molecular dynamics where continuous force potentials are present. The *continuous* contact detection algorithm developed by Wang and his collaborators [6, 51, 52] is also well formulated mathematically, but it is too computationally expensive for the evaluation of the force in the quasi-static regime and therefore will not be described here. Nevertheless, we will make connections to those theories in Sects. 3.5 and 3.9. Unfortunately, the most computationally efficient algorithms are not always expressed with the same level of rigor, and the topic has suffered from some of this confusion.

In practice, every contact detection algorithm for ellipses should provide a single contact point, a single contact normal, and either a separation or a penetration distance. However, given two elliptical particles^2^${\mathcal {E}}_i$ and ${\mathcal {E}}_j \subset {\mathbb {R}}^2$, and two points judiciously constructed on each particle, say ${\varvec{x}}_i \in {\mathcal {E}}_i$ and ${\varvec{x}}_j \in {\mathcal {E}}_j$, one could compute the distance between ${\varvec{x}}_i$ and ${\varvec{x}}_j$ as the separation or penetration distance and define the midpoint between ${\varvec{x}}_i$ and ${\varvec{x}}_j$ as the contact point (which should provide reasonable approximations of the contact properties in case of ellipses with small overlap). The contact normal could then be defined in terms of the segment joining ${\varvec{x}}_i$ to ${\varvec{x}}_j$. For most of the algorithms, we shall describe in Sect. 5, this is precisely how the contact point and contact normal are actually computed.

Although the regularity of functions usually plays an important role in numerical analysis, all the objects studied below will be expressed as algebraic constraints between real analytic functions and hence will also be real analytic, which in practice means all functions will be infinitely differentiable away from a finite number of obvious singularities.

**Fig. 3:**
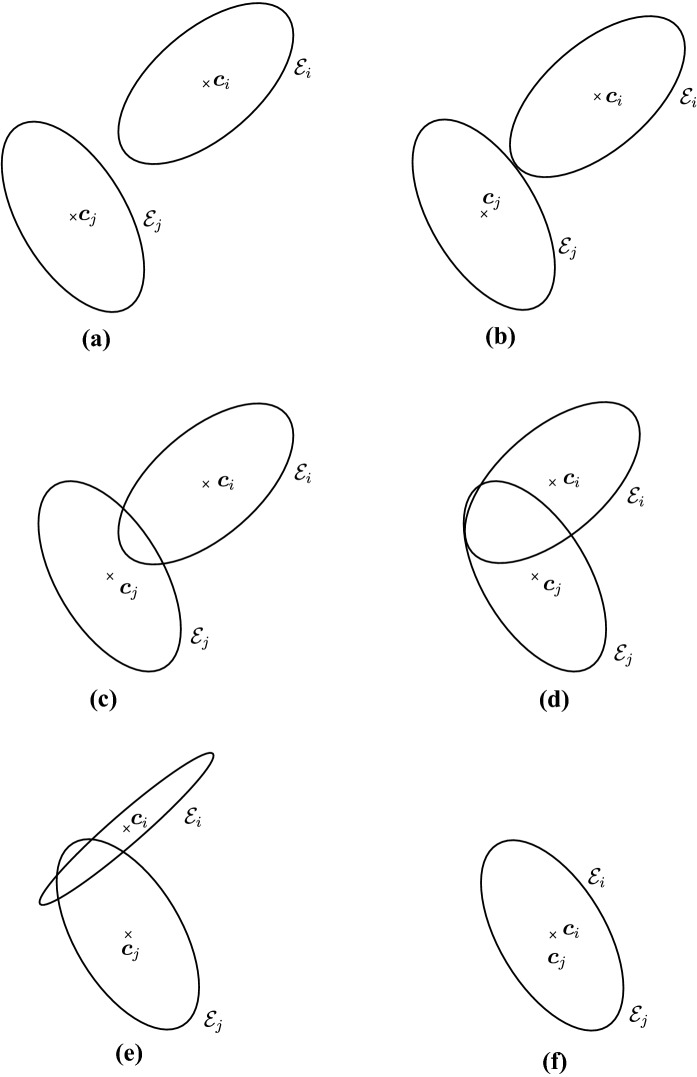
Illustration of possible configurations for a pair of ellipses ${\mathcal {E}}_i$ and ${\mathcal {E}}_j$: **a** disjoint ellipses with no overlap, **b** ellipses in perfect contact (see Definition 4), **c** ellipses with two intersection points, **d** ellipses with three intersection points, **e** ellipses with four intersection points, **f**) ellipses that perfectly coincide. Note that all configurations satisfy the non-penetrating CoM condition, except (**f**)

Estimating the contact point between all possible configurations of pairs of ellipses is in general not necessary in particle simulations and can lead to a number of degenerate cases, such as when the center of mass of one of the ellipses is inside the area of the second ellipse or when one ellipse is virtually penetrating completely through the other. We will therefore restrict ourselves to a limited number of configurations of the ellipses which we may encounter, illustrated in Fig. 3, thus avoiding degenerate contacts. Wang and his collaborators have produced a complete classification of all configurations, including degenerate contacts, using the theory of arrangements from computational geometry [27]. The first few definitions and lemmas below aim at characterizing such configurations, which we will refer to as *near-perfect contact*. When pairs of ellipses are in near-perfect contact, Theorem 7 will show that only a few cases need to be studied.

### Intersection of ellipses

#### Definition 2

(*Intersection set*) Let ${\mathcal {E}}_i$, ${\mathcal {E}}_j \subset {\mathbb {R}}^2$ be two ellipses. Their intersection set is defined as

38 $$\begin{aligned} {\mathcal {I}}_{ij} = {\mathcal {E}}_i \cap {\mathcal {E}}_j . \end{aligned}$$

Before proceeding with an analysis of the intersection set, observe that it is, at least formally, computable as the solution to a minimization problem when ${\mathcal {I}}_{ij} \ne \varnothing$. Although not the only possible formulation, this section will show that its connection to other auxiliary problems makes it the richest formulation.

#### Lemma 2

Given two ellipses ${\mathcal {E}}_i$ and ${\mathcal {E}}_j$ such that ${\mathcal {I}}_{ij} \ne \varnothing$, all solutions

39 $$\begin{aligned} {\mathcal {X}}_{ij} := \mathop {\hbox {argmin}}\limits _{({\hat{{\varvec{x}}}}_i,{\hat{{\varvec{x}}}}_j) \in {\mathcal {E}}_i \times {\mathcal {E}}_j} \Vert {\hat{{\varvec{x}}}}_i - {\hat{{\varvec{x}}}}_j\Vert \, , \end{aligned}$$

satisfy ${\hat{{\varvec{x}}}}_i = {\hat{{\varvec{x}}}}_j := {\hat{{\varvec{x}}}}$. According to this identification, the set of pairs $({\hat{{\varvec{x}}}},{\hat{{\varvec{x}}}}) \in {\mathcal {X}}_{ij}$ correspond to the points ${\hat{{\varvec{x}}}} \in {\mathcal {I}}_{ij}$.

#### Proof

If the intersection is not empty, then there exists a point ${\varvec{x}}\in {\mathcal {E}}_i \cap {\mathcal {E}}_j$ for which the pair $({\varvec{x}},{\varvec{x}}) \in {\mathcal {E}}_i \times {\mathcal {E}}_j$ and the distance $\Vert {\varvec{x}}- {\varvec{x}}\Vert$ vanishes. Any other pair of points $({\hat{{\varvec{x}}}}_i,{\hat{{\varvec{x}}}}_j) \in {\mathcal {E}}_i \times {\mathcal {E}}_j$ for which $\Vert {\hat{{\varvec{x}}}}_i - {\hat{{\varvec{x}}}}_j\Vert$ reaches the minimum value, which we have shown is zero, must satisfy ${\varvec{x}}_i = {\varvec{x}}_j$. Thus, the single point ${\varvec{x}}_i={\varvec{x}}_j$ must belong to both ${\mathcal {E}}_i$ and ${\mathcal {E}}_j$. $\square$

Intuitively, it is easy to imagine the different forms that the intersection set (see Fig. 3) may take, but it is less straightforward to give a complete and thorough description. Bézout’s theorem [15, 23] applied to the roots of two quadratic bivariate polynomials in ${\mathbb {R}}^2$ states that the intersection set ${\mathcal {I}}_{ij}$ can be either

1. empty: the ellipses are disjoint;
2. one point: the ellipses are in perfect contact (see Definition 4);
3. two, three, or four points: the ellipses intersect;
4. or an entire ellipse, if the two ellipses coincide.

We first state the following definition that will allow us to disregard trivial cases such as when one ellipse lies fully within a second ellipse or when two ellipses coincide. Although this condition will appear innocuous, it will play a key role in Lemma 11 and be a geometrical motivation for the numerical condition (60).

#### Definition 3

(*Ellipses with non-penetrating centers of mass*) Two ellipses ${\mathcal {E}}_i$, ${\mathcal {E}}_j \subset {\mathbb {R}}^2$ are said to have non-penetrating centers of mass (CoM) if the distances between the centers, evaluated in both the ${\mathcal {E}}_i$- and ${\mathcal {E}}_j$-norms (7), satisfy

40 $$\begin{aligned}&\Vert {\varvec{c}}_j - {\varvec{c}}_i \Vert _{{\mathcal {E}}_i} \ge 1, \end{aligned}$$

41 $$\begin{aligned}&\Vert {\varvec{c}}_i - {\varvec{c}}_j \Vert _{{\mathcal {E}}_j} \ge 1. \end{aligned}$$

### Case of two disjoint ellipses

In this section, we consider the case of two disjoint ellipses ${\mathcal {E}}_i$ and ${\mathcal {E}}_j$, i.e., ${\mathcal {I}}_{ij} = \varnothing$, that satisfy the non-penetrating CoM condition, see Definition 3. In this case, there is obviously no contact nor overlap, but one can estimate the distance between the two particles. Our objective in doing so is to find formulations of the separation distance that can be extended to the definition of distance, or contact point, when the ellipses are overlapping.

The most obvious and straightforward formulation of the distance between two ellipses, which could naturally be applied to any pair of objects, is characterized in the following lemma.

#### Lemma 3

(Minimum Distance Pair) Let ${\mathcal {E}}_i$ and ${\mathcal {E}}_j$ be two disjoint ellipses with non-penetrating CoM. Then, there exists a unique pair of points $({\varvec{x}}_i,{\varvec{x}}_j) \in {\mathcal {E}}_i \times {\mathcal {E}}_j$ that minimize the Euclidean norm $\Vert {\varvec{x}}_i -{\varvec{x}}_j\Vert$, i.e.,

42 $$\begin{aligned} ({\varvec{x}}_i,{\varvec{x}}_j) &= \mathop {\hbox {argmin}}\limits _{({\hat{{\varvec{x}}}}_i,{\hat{{\varvec{x}}}}_j) \in {\mathcal {E}}_i \times {\mathcal {E}}_j} \Vert {\hat{{\varvec{x}}}}_i - {\hat{{\varvec{x}}}}_j\Vert  \nonumber \\&= \mathop {\hbox {argmin}}\limits _{({\hat{{\varvec{x}}}}_i,{\hat{{\varvec{x}}}}_j) \in {\mathcal {E}}_i \times {\mathcal {E}}_j} \frac{1}{2} \Vert {\hat{{\varvec{x}}}}_i - {\hat{{\varvec{x}}}}_j\Vert ^2. \end{aligned}$$

Moreover, at the minimum, the unit normal vectors ${\varvec{n}}_i({\varvec{x}}_i)$ and ${\varvec{n}}_j({\varvec{x}}_j)$ to ${\mathcal {E}}_i$ and ${\mathcal {E}}_j$, respectively, are opposite

43 $$\begin{aligned} {\varvec{n}}_i({\varvec{x}}_i) + {\varvec{n}}_j( {\varvec{x}}_j) = {\varvec{0}}. \end{aligned}$$

The pair $({\varvec{x}}_i,{\varvec{x}}_j) \in {\mathcal {E}}_i \times {\mathcal {E}}_j$ will be referred to as the minimum distance pair (MDP) of ellipses ${\mathcal {E}}_i$ and ${\mathcal {E}}_j$.

#### Proof

The existence of a unique pair $({\varvec{x}}_i,{\varvec{x}}_j) \in {\mathcal {E}}_i \times {\mathcal {E}}_j$ that minimizes distance $\Vert {\varvec{x}}_i - {\varvec{x}}_j\Vert$ follows from elementary results in linear algebra [43], which we now present.

Consider the convex and compact set $E_k := \big \{ {\varvec{x}}\in {\mathbb {R}}^2;\ f_k({\varvec{x}}) \le 0 \big \}$, $k=i,j$, formed by ${\mathcal {E}}_k$ and its *interior*. Since ${\mathcal {E}}_i$ and ${\mathcal {E}}_j$ are disjoint and have non-penetrating CoM, then $E_i \cap E_j = \varnothing$. It implies that the set

$$\begin{aligned} D := \big \{ {\varvec{d}}\in {\mathbb {R}}^2 ;\ {\varvec{d}}= {\varvec{x}}_i - {\varvec{x}}_j,\ \forall ({\varvec{x}}_i,{\varvec{x}}_j) \in E_i \times E_j \big \} \end{aligned}$$

is also compact and convex. Hence, there exists a unique ${\varvec{d}}\in D$ minimizing $\Vert {\varvec{d}}\Vert$, according to the Hilbert projection theorem. In fact, if ${\varvec{d}}= {\varvec{x}}_i - {\varvec{x}}_j$ for $({\varvec{x}}_i,{\varvec{x}}_j) \in E_i \times E_j$, then the pair must belong to ${\mathcal {E}}_i \times {\mathcal {E}}_j$. If not, say ${\varvec{x}}_i \in E_i {\setminus } {\mathcal {E}}_i$, then one could always find an $\varepsilon$, $0 < \varepsilon \ll 1$, such that ${\varvec{x}}_i - \varepsilon {\varvec{d}}\in E_i$ and the pair $({\varvec{x}}_i-\epsilon {\varvec{d}},{\varvec{x}}_j)$ would define a smaller distance

$$\begin{aligned} \big \Vert ({\varvec{x}}_i - \varepsilon {\varvec{d}}) - {\varvec{x}}_j \big \Vert = \big \Vert (1-\varepsilon ) {\varvec{d}}\big \Vert < \Vert {\varvec{d}}\Vert . \end{aligned}$$

Finally, we observe that the pair $({\varvec{x}}_i , {\varvec{x}}_j) \in {\mathcal {E}}_i \times {\mathcal {E}}_j$ must be unique. Indeed, if one can find $({\varvec{y}}_i , {\varvec{y}}_j) \in {\mathcal {E}}_i \times {\mathcal {E}}_j$ such that ${\varvec{d}}= {\varvec{x}}_i - {\varvec{x}}_j = {\varvec{y}}_i - {\varvec{y}}_j$, then all pairs

$$\begin{aligned} (1-\lambda ) \big ( {\varvec{x}}_i, {\varvec{x}}_j \big ) + \lambda \big ( {\varvec{y}}_i, {\varvec{y}}_j\big ), \quad \forall \lambda \in [0,1], \end{aligned}$$

would also minimize distance

$$\begin{aligned} \begin{aligned}&\big \Vert \big ( (1-\lambda ) {\varvec{x}}_i + \lambda {\varvec{y}}_i \big ) - \big ( (1-\lambda ) {\varvec{x}}_j + \lambda {\varvec{y}}_j\big ) \big \Vert \\&\quad = \big \Vert (1-\lambda )({\varvec{x}}_i - {\varvec{x}}_j) + \lambda ({\varvec{y}}_i - {\varvec{y}}_j) \big \Vert \\&\quad \le (1-\lambda ) \Vert {\varvec{x}}_i - {\varvec{x}}_j\Vert + \lambda \Vert {\varvec{y}}_i - {\varvec{y}}_j\Vert = \Vert {\varvec{d}}\Vert \end{aligned} \end{aligned}$$

and thus, by virtue of the previous result, should lie on the boundaries of ${\mathcal {E}}_i$ and ${\mathcal {E}}_j$. However, the points $(1-\lambda ) {\varvec{x}}_i + \lambda {\varvec{y}}_i$, (resp. $(1-\lambda ) {\varvec{x}}_j + \lambda {\varvec{y}}_j$), $\forall \lambda \in [0,1]$, form a straight segment and cannot lie on the boundary of ${\mathcal {E}}_i$ (resp. ${\mathcal {E}}_j$), since the ellipses are strictly convex. This shows that the pair $({\varvec{x}}_i,{\varvec{x}}_j) \in {\mathcal {E}}_i \times {\mathcal {E}}_j$ is unique.

Let $f_i$ and $f_j$ be the global potentials of ${\mathcal {E}}_i$ and ${\mathcal {E}}_j$, respectively. In order to show that the unit normal vectors are opposite, we introduce the Lagrangian functional:

44 $$\begin{aligned} {\mathcal {L}}({\hat{{\varvec{x}}}}_i,{\hat{{\varvec{x}}}}_j, \lambda _i,\lambda _j) = \frac{1}{2} \Vert {\hat{{\varvec{x}}}}_i - {\hat{{\varvec{x}}}}_j\Vert ^2 - \lambda _i f_i({\hat{{\varvec{x}}}}_i) - \lambda _j f_j({\hat{{\varvec{x}}}}_j)\nonumber \\ \end{aligned}$$

where $\lambda _i \in {\mathbb {R}}$ and $\lambda _j \in {\mathbb {R}}$ denote the Lagrange multipliers associated with the constraints $f_i({\hat{{\varvec{x}}}}_i) = 0$ and $f_j({\hat{{\varvec{x}}}}_j)=0$, i.e., ${\hat{{\varvec{x}}}}_i \in {\mathcal {E}}_i$ and ${\hat{{\varvec{x}}}}_j \in {\mathcal {E}}_j$, respectively, in the minimization problem (42). The derivative ${\mathcal {L}}_{({\hat{{\varvec{x}}}}_i,{\hat{{\varvec{x}}}}_j)}$ of ${\mathcal {L}}$ with respect to $({\hat{{\varvec{x}}}}_i,{\hat{{\varvec{x}}}}_j)$ is given, $\forall ({\varvec{v}}_i,{\varvec{v}}_j) \in {\mathbb {R}}^2 \times {\mathbb {R}}^2$, by

$$\begin{aligned}&{\mathcal {L}}_{({\hat{{\varvec{x}}}}_i,{\hat{{\varvec{x}}}}_j)} ({\hat{{\varvec{x}}}}_i,{\hat{{\varvec{x}}}}_j, \lambda _i,\lambda _j, {\varvec{v}}_i,{\varvec{v}}_j) \\&\quad = \big [ ({\hat{{\varvec{x}}}}_i-{\hat{{\varvec{x}}}}_j) - \lambda _i \nabla f_i ({\hat{{\varvec{x}}}}_i) \big ] \cdot {\varvec{v}}_i \\&\qquad + \, \big [ - ({\hat{{\varvec{x}}}}_i-{\hat{{\varvec{x}}}}_j) - \lambda _j \nabla f_j ({\hat{{\varvec{x}}}}_j) \big ] \cdot {\varvec{v}}_j. \end{aligned}$$

The solution $({\varvec{x}}_i,{\varvec{x}}_j)$ to (42) is a stationary point of ${\mathcal {L}}$ and must satisfy

$$\begin{aligned} {\mathcal {L}}_{({\varvec{x}}_i,{\varvec{x}}_j)} ({\varvec{x}}_i,{\varvec{x}}_j, \lambda _i,\lambda _j, {\varvec{v}}_i, {\varvec{v}}_j) = 0, \quad \forall ({\varvec{v}}_i,{\varvec{v}}_j) \in {\mathbb {R}}^2 \times {\mathbb {R}}^2, \end{aligned}$$

or, equivalently,

45 $$\begin{aligned}&({\varvec{x}}_i-{\varvec{x}}_j) - \lambda _i \nabla f_i ({\varvec{x}}_i) = {\varvec{0}}, \end{aligned}$$

46 $$\begin{aligned}&({\varvec{x}}_i-{\varvec{x}}_j) + \lambda _j \nabla f_j ({\varvec{x}}_j) = {\varvec{0}}. \end{aligned}$$

Combining those two equations leads to

$$\begin{aligned} \lambda _i \nabla f_i ({\varvec{x}}_i) + \lambda _j \nabla f_j ({\varvec{x}}_j) = {\varvec{0}}, \end{aligned}$$

meaning that the gradients $\nabla f_i ({\varvec{x}}_i)$ and $\nabla f_j ({\varvec{x}}_j)$ share the same or opposite direction. The identity (45) states that

$$\begin{aligned} \lambda _i \nabla f_i({\varvec{x}}_i) = ({\varvec{x}}_i-{\varvec{x}}_j) \end{aligned}$$

but since $\nabla f_i({\varvec{x}}_i)$ points out of $E_i$, while, in the case that ${\mathcal {E}}_i$ and ${\mathcal {E}}_j$ are disjoint and satisfy the property of non-penetrating CoM, $({\varvec{x}}_i-{\varvec{x}}_j)$ points inside $E_i$, we can conclude that $\lambda _i<0$. Similarly, the identity (46) shows that $\lambda _j<0$. Since both Lagrange multipliers are of the same sign,

$$\begin{aligned} {\varvec{0}}= & {} \lambda _i \nabla f_i ({\varvec{x}}_i) + \lambda _j \nabla f_j ({\varvec{x}}_j) = \lambda _i \Vert \nabla f_i ({\varvec{x}}_i) \Vert \, {\varvec{n}}_i({\varvec{x}}_i) \\&\quad +\, \lambda _j \Vert \nabla f_j ({\varvec{x}}_j) \Vert \, {\varvec{n}}_j({\varvec{x}}_j), \end{aligned}$$

which allows one to conclude that (43) is satisfied. $\square$

It is worth noting that (43) represents a non-binding constraint as it is automatically verified by the solution to the minimization problem. Therefore, (42) can be recast as

47 $$\begin{aligned} ({\varvec{x}}_i,{\varvec{x}}_j)  =  \mathop {\hbox {argmin}}\limits _{ \begin{array}{c} ({\hat{{\varvec{x}}}}_i,{\hat{{\varvec{x}}}}_j) \in {\mathcal {E}}_i \times {\mathcal {E}}_j \\ {\varvec{n}}_i({\hat{{\varvec{x}}}}_i) + {\varvec{n}}_j({\hat{{\varvec{x}}}}_j) = {\varvec{0}} \end{array}}  \Vert {\hat{{\varvec{x}}}}_i - {\hat{{\varvec{x}}}}_j\Vert  =  \mathop {\hbox {argmin}}\limits _{ \begin{array}{c} ({\hat{{\varvec{x}}}}_i,{\hat{{\varvec{x}}}}_j) \in {\mathcal {E}}_i \times {\mathcal {E}}_j \\ {\varvec{n}}_i({\hat{{\varvec{x}}}}_i) + {\varvec{n}}_j({\hat{{\varvec{x}}}}_j) = {\varvec{0}} \end{array} }  \frac{1}{2} \Vert {\hat{{\varvec{x}}}}_i - {\hat{{\varvec{x}}}}_j\Vert ^2.\nonumber \\ \end{aligned}$$

#### Remark 2

Although Lemma 3 is stated only for pairs of ellipses, the statement and the proof clearly also apply to pairs of ellipsoids.

#### Remark 3

In the case of two disjoint circles ${\mathcal {C}}_i$ and ${\mathcal {C}}_j$, the solution pair $({\varvec{x}}_i,{\varvec{x}}_j)$ to (42) is actually aligned with the centers of the circles, ${\varvec{c}}_i$ and ${\varvec{c}}_j$. From this observation, one can reformulate the distance $\Vert {\varvec{x}}_i - {\varvec{x}}_j \Vert$ as

$$\begin{aligned} \Vert {\varvec{x}}_i - {\varvec{x}}_j \Vert = \Vert {\varvec{x}}_i - {\varvec{c}}_j \Vert + \Vert {\varvec{x}}_j - {\varvec{c}}_i \Vert - \Vert {\varvec{c}}_i - {\varvec{c}}_j \Vert \end{aligned}$$

so that the minimization problem (42) can be recast as

$$\begin{aligned}&\min _{({\hat{{\varvec{x}}}}_i,{\hat{{\varvec{x}}}}_j) \in {\mathcal {C}}_i \times {\mathcal {C}}_j} \big [ \Vert {\hat{{\varvec{x}}}}_i - {\varvec{c}}_j \Vert + \Vert {\hat{{\varvec{x}}}}_j - {\varvec{c}}_i \Vert \big ] \\&\qquad \quad = \min _{{\hat{{\varvec{x}}}}_i \in {\mathcal {C}}_i } \Vert {\hat{{\varvec{x}}}}_i - {\varvec{c}}_j\Vert  + \min _{{\hat{{\varvec{x}}}}_j \in {\mathcal {C}}_j} \Vert {\hat{{\varvec{x}}}}_j - {\varvec{c}}_i\Vert . \end{aligned}$$

It follows that the minimization problem can be separated into the fully decoupled minimization problems

$$\begin{aligned} \begin{aligned}&{\varvec{x}}_i = \mathop {\hbox {argmin}}\limits _{{\varvec{x}}\in {\mathcal {C}}_i} \Vert {\varvec{x}}- {\varvec{c}}_j \Vert , \\&{\varvec{x}}_j = \mathop {\hbox {argmin}}\limits _{{\varvec{x}}\in {\mathcal {C}}_j} \Vert {\varvec{x}}- {\varvec{c}}_i \Vert . \end{aligned} \end{aligned}$$

In other words, the points ${\varvec{x}}_i$ and ${\varvec{x}}_j$ are the closest points on ${\mathcal {C}}_i$ and ${\mathcal {C}}_j$ to the centers ${\varvec{c}}_j$ and ${\varvec{c}}_i$, respectively. Recalling that ellipses can be viewed as circles in their respective ${\mathcal {E}}$-norms, one can actually introduce similar decoupled minimization problems in the case of ellipses.

#### Lemma 4

(Minimum Potential Pair) Let ${\mathcal {E}}_i$ and ${\mathcal {E}}_j$ be two disjoint ellipses with non-penetrating CoM with global potentials $f_i$ and $f_j$, respectively. Then, there exists a unique pair of points $({\varvec{x}}_i,{\varvec{x}}_j) \in {\mathcal {E}}_i \times {\mathcal {E}}_j$ satisfying the two problems

48 $$\begin{aligned}&{\varvec{x}}_i = \mathop {\hbox {argmin}}\limits _{{\varvec{x}}\in {\mathcal {E}}_i} \Vert {\varvec{x}}- {\varvec{c}}_j \Vert _{{\mathcal {E}}_j} = \mathop {\hbox {argmin}}\limits _{{\varvec{x}}\in {\mathcal {E}}_i} f_j({\varvec{x}}), \end{aligned}$$

49 $$\begin{aligned}&{\varvec{x}}_j = \mathop {\hbox {argmin}}\limits _{{\varvec{x}}\in {\mathcal {E}}_j} \Vert {\varvec{x}}- {\varvec{c}}_i \Vert _{{\mathcal {E}}_i} = \mathop {\hbox {argmin}}\limits _{{\varvec{x}}\in {\mathcal {E}}_j} f_i({\varvec{x}}). \end{aligned}$$

Moreover, following the convention (29), we have

50 $$\begin{aligned}&{\varvec{n}}_i ({\varvec{x}}_i) + {\varvec{n}}_j({\varvec{x}}_i) = {\varvec{0}}, \end{aligned}$$

51 $$\begin{aligned}&{\varvec{n}}_i({\varvec{x}}_j) + {\varvec{n}}_j({\varvec{x}}_j) = {\varvec{0}}. \end{aligned}$$

The unique pair $({\varvec{x}}_i, {\varvec{x}}_j) \in {\mathcal {E}}_i \times {\mathcal {E}}_j$ will be referred to as the minimum potential pair (MPP) with respect to the *i*-norm and the *j*-norm.

#### Proof

Since $f_j({\varvec{x}}) = \Vert {\varvec{x}}- {\varvec{c}}_j \Vert _{{\mathcal {E}}_j}^2 - 1$, it follows that the two minimization problems in (48) are equivalent. The same reasoning implies that the two minimization problems in (49) are also equivalent. The demonstration of the existence and uniqueness to the minimization problems (48), or (49), is similar to the one given in Lemma 3. Consider the minimization problem

52 $$\begin{aligned} \min _{{\varvec{x}}\in E_i} \Vert {\varvec{x}}- {\varvec{c}}_j \Vert _{{\mathcal {E}}_j}, \end{aligned}$$

where $E_i = \{ {\varvec{x}}\in {\mathbb {R}}^2;\ f_i({\varvec{x}}) \le 0 \}$ is compact and strictly convex. Then, it is well known that (52) has a unique solution, say ${\varvec{x}}_i$. As we argued earlier, ${\varvec{x}}_i$ must in fact belong to the boundary ${\mathcal {E}}_i$ and is unique because $E_i$ is strictly convex.

The Lagrangian functional associated with the constrained minimization problem (48) is given by

$$\begin{aligned} {\mathcal {L}}_i ({\varvec{x}}, \lambda ) = f_j({\varvec{x}}) - \lambda f_i({\varvec{x}}). \end{aligned}$$

Since the solution ${\varvec{x}}_i$ to (48) is a stationary point of ${\mathcal {L}}_i$, it necessarily satisfies

$$\begin{aligned} \nabla f_j({\varvec{x}}_i) - \lambda \nabla f_i({\varvec{x}}_i) = {\varvec{0}}, \end{aligned}$$

which, using the fact that the two ellipses are disjoint and have non-penetrating CoM, implies that the two normals at ${\varvec{x}}_i$ are in opposite direction. Indeed, as was explained in the proof of Lemma 3, it is easy to see that $\lambda < 0$, hence

$$\begin{aligned} {\varvec{n}}_i ({\varvec{x}}_i) + {\varvec{n}}_j({\varvec{x}}_i) = {\varvec{0}}. \end{aligned}$$

The relation (51) is shown in the same manner by introducing the Lagrangian functional ${\mathcal {L}}_j$ associated with the minimization problem (49). $\square$

Since the relations (50) and (51) are satisfied at the points of the MPP $({\varvec{x}}_i,{\varvec{x}}_j)$, they can each be added to the minimization problems (48) and (49), respectively, as non-binding constraints so that the two problems can be recast as

53 $$\begin{aligned}&{\varvec{x}}_i  =  \mathop {\hbox {argmin}}\limits _{\begin{array}{c} {\varvec{x}}\in {\mathcal {E}}_i \\ {\varvec{n}}_i({\varvec{x}})+{\varvec{n}}_j({\varvec{x}}) = {\varvec{0}} \end{array}}  \Vert {\varvec{x}}- {\varvec{c}}_j \Vert _{{\mathcal {E}}_j}  =  \mathop {\hbox {argmin}}\limits _{\begin{array}{c} {\varvec{x}}\in {\mathcal {E}}_i \\ {\varvec{n}}_i({\varvec{x}})+{\varvec{n}}_j({\varvec{x}}) = {\varvec{0}} \end{array}}  f_j({\varvec{x}}), \end{aligned}$$

and

54 $$\begin{aligned}&{\varvec{x}}_j  =  \mathop {\hbox {argmin}}\limits _{\begin{array}{c} {\varvec{x}}\in {\mathcal {E}}_j \\ {\varvec{n}}_i({\varvec{x}})+{\varvec{n}}_j({\varvec{x}}) = {\varvec{0}} \end{array}}  \Vert {\varvec{x}}- {\varvec{c}}_i \Vert _{{\mathcal {E}}_i}  =  \mathop {\hbox {argmin}}\limits _{\begin{array}{c} {\varvec{x}}\in {\mathcal {E}}_j \\ {\varvec{n}}_i({\varvec{x}})+{\varvec{n}}_j({\varvec{x}}) = {\varvec{0}} \end{array}}  f_i({\varvec{x}}). \end{aligned}$$

#### Remark 4

As we observed earlier after Lemma 3, the statement and proof of Lemma 4 apply equally well to pairs of disjoint ellipsoids.

**Fig. 4:**
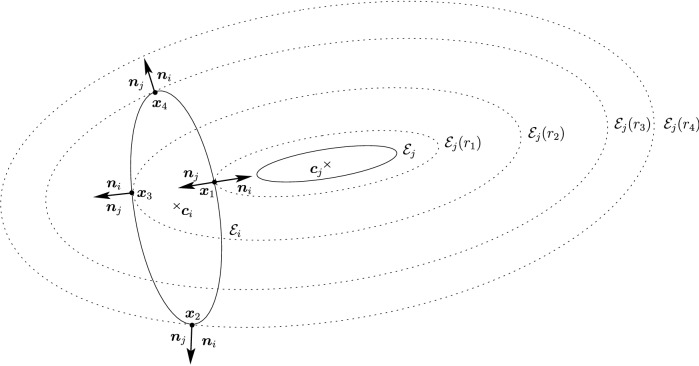
Illustration that shows that Problem (48) may have up to four critical points ${\varvec{x}}_{k}$, $k=1,\cdots ,4$. One can observe that ${\varvec{n}}_i({\varvec{x}}_k)+{\varvec{n}}_j({\varvec{x}}_k)={\varvec{0}}$ only if $k=1$. The ellipses ${\mathcal {E}}_j(r_k)$ are dilations of ${\mathcal {E}}_j$ defined in Sect. 2.3

Before proceeding with the other cases, we will make a few remarks on the solution to Problem (48). One classical approach for solving the constrained minimization problem proceeds by means of Lagrange multipliers, as seen earlier. However, the resulting problem could lead to several solutions as the nonlinear Lagrangian functional may have up to four critical points depending on the configuration and size of the ellipses. In other words, the solutions correspond to local minima and maxima of the potential function $f_j$ restricted to ${\mathcal {E}}_i$. This is exemplified in Fig. 4. In practice, all of the known methods identify all of the critical points and distinguish the global minimum by explicitly evaluating the distance at each critical point.

It is worth noting here that the non-binding constraint in the minimization problem (53) has the added benefit of yielding a Lagrangian functional with a unique critical point. Indeed, the constraint (50) is only satisfied at the global minimum. Alternatively, one may enforce the uniqueness of the critical point by considering the inequality constraint ${\varvec{n}}_i({\varvec{x}}) \cdot {\varvec{n}}_j({\varvec{x}}) < 0$ or $\nabla f_i({\varvec{x}})\cdot \nabla f_j({\varvec{x}})<0$.

### Case of two ellipses in perfect contact

The case of perfect contact between ellipses with non-penetrating CoM can be viewed as a limiting case of two disjoint ellipses. Therefore, we shall quickly verify that the previous results straightforwardly apply to this particular case.

#### Definition 4

(*Perfect contact point*)  Two ellipses ${\mathcal {E}}_i$, ${\mathcal {E}}_j \subset {\mathbb {R}}^2$ are said to be in perfect contact if ${\mathcal {I}}_{ij}$ consists of a single point and if they have non-penetrating CoM. That point is then called a perfect contact point.

#### Lemma 5

Let ${\mathcal {E}}_i$ and ${\mathcal {E}}_j$ be two ellipses in perfect contact at point ${\varvec{x}}_c$ with non-penetrating CoM. Moreover, let ${\varvec{n}}_i({\varvec{x}}_c)$ and ${\varvec{n}}_j({\varvec{x}}_c)$ denote the outward normal unit vectors at ${\varvec{x}}_c$ to ${\mathcal {E}}_i$ and ${\mathcal {E}}_j$, respectively. Then, the pair $({\varvec{x}}_c,{\varvec{x}}_c)$ is the MDP and MPP of the two ellipses. Moreover, it holds that

55 $$\begin{aligned} {\varvec{n}}_i({\varvec{x}}_c) + {\varvec{n}}_j( {\varvec{x}}_c) = {\varvec{0}}. \end{aligned}$$

#### Proof

We first show that $({\varvec{x}}_c,{\varvec{x}}_c)$ is the MDP. If ${\varvec{x}}_c$ is a perfect contact point, then for all $({\varvec{x}}_i,{\varvec{x}}_j) \in {\mathcal {E}}_i \times {\mathcal {E}}_j$ such that $({\varvec{x}}_i,{\varvec{x}}_j) \ne ({\varvec{x}}_c,{\varvec{x}}_c)$, the distance $\Vert {\varvec{x}}_i - {\varvec{x}}_j\Vert > 0$. Hence, $({\varvec{x}}_c,{\varvec{x}}_c)$ is the unique solution to (42). To show that $({\varvec{x}}_c,{\varvec{x}}_c)$ is the MPP, we observe that for any point ${\varvec{x}}\in {\mathcal {E}}_i {\setminus }\{ {\varvec{x}}_c\}$, ${\varvec{x}}\notin {\mathcal {E}}_j$ and therefore $f_j({\varvec{x}}) > 0$. Hence,

$$\begin{aligned} 1 = \Vert {\varvec{x}}_c - {\mathbf {c}}_j \Vert _{{\mathcal {E}}_j} < \Vert {\varvec{x}}- {\mathbf {c}}_j \Vert _{{\mathcal {E}}_j}. \end{aligned}$$

This shows that ${\varvec{x}}_c$ is the unique solution to (48), and, in a similar manner, ${\varvec{x}}_c$ is also the unique solution to (49). Finally, the relation (55) is clearly a consequence of (50) and (51) when ${\varvec{x}}_c = {\varvec{x}}_i = {\varvec{x}}_j$. $\square$

### Case of two ellipses with overlap

The goal of this section is to extend the definitions of MDP and MPP to pairs of ellipses with *small overlap* and to establish existence and uniqueness of those pairs. The description of the geometry at the intersection of ellipses with *small overlap* will therefore be a prerequisite for the analysis of a contact detection problem. Unfortunately, to make this intuitive notion precise we will need to introduce the *co-gradient locus* which formalizes the fundamental constraints (50)–(51) on the normals. In order to emphasize the key concepts and the structure of the theory, the proofs of the results will be delayed to Sects. 3.6 and 3.7. In contrast to the previous sections, most of the results below have only been established in ${\mathbb {R}}^2$ and we will delay to Sect. 3.8 any speculation on extensions to ${\mathbb {R}}^3$.

The co-gradient locus is the set of points ${\varvec{x}}$ where ${\varvec{n}}_i({\varvec{x}}) \propto {\varvec{n}}_j({\varvec{x}})$, which includes points where (50)–(51) are satisfied. To the best of our knowledge, this set was first introduced in [45, 46] where it was called the *locus of common slope* but was not characterized.

#### Definition 5

(*Co-gradient function and co-gradient locus*) Given two ellipses ${\mathcal {E}}_i$, ${\mathcal {E}}_j \subset {\mathbb {R}}^2$, the co-gradient function is defined as

56 $$\begin{aligned} {\varvec{h}}({\varvec{x}}) := \nabla f_i({\varvec{x}}) \times \nabla f_j({\varvec{x}}). \end{aligned}$$

The associated co-gradient locus is the set of all roots of the co-gradient function, i.e.,

57 $$\begin{aligned} {\mathcal {H}}_{ij} := \big \{ {\varvec{x}}\in {\mathbb {R}}^2;\ {\varvec{h}}({\varvec{x}}) = {\varvec{0}} \big \} . \end{aligned}$$

**Fig. 5:**
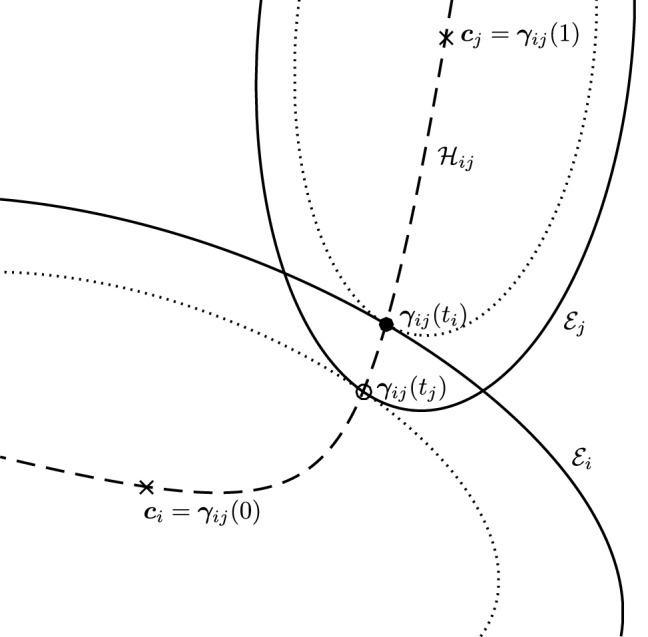
Illustration of the smooth injection $\varvec{\gamma }_{ij}$ onto the gradient locus ${\mathcal {H}}_{ij}$, as formulated in Theorem 6. The smooth injection $\varvec{\gamma }_{ij}$ from Theorem 6 is a single component of the hyperbola ${\mathcal {H}}_{ij}$ which passes through both centers ${\varvec{c}}_i$ ad ${\varvec{c}}_j$

In Sect. 3.6, we will provide a few alternative descriptions of ${\varvec{h}}$ and its zero set ${\mathcal {H}}_{ij}$. The previous definition has an obvious analogue in ${\mathbb {R}}^3$. Building on Lemma 1, it is possible to prove the following elegant result illustrated in Fig. 5.

#### Theorem 6

(Co-gradient locus) Let ${\mathcal {E}}_i$, ${\mathcal {E}}_j \subset {\mathbb {R}}^2$ be two ellipses with non-penetrating CoM. Then, the co-gradient locus ${\mathcal {H}}_{ij}$ is a hyperbola and the two centers of the ellipses belong to the same branch of the hyperbola. The branch of ${\mathcal {H}}_{ij}$ between ${\varvec{c}}_i$ and ${\varvec{c}}_j$ can be parameterized by a smooth injection $\varvec{\gamma }_{ij} : [0,1] \longrightarrow {\mathcal {H}}_{ij}$ satisfying

58 $$\begin{aligned} \left\{ \begin{aligned}&\varvec{\gamma }_{ij}(0) = {\varvec{c}}_i, \\&\varvec{\gamma }_{ij}(1) = {\varvec{c}}_j. \end{aligned} \right. \end{aligned}$$

Moreover, there exists a unique pair of parameters $t_k \in ]0,1[$, for $k=i,j$, such that

59 $$\begin{aligned} \varvec{\gamma }_{ij}(t_k) \in {\mathcal {E}}_k. \end{aligned}$$

A long and detailed proof of this result can be found in Sect. 3.6, but there exists a shorter proof sketched out in Remark 6 of Sect. 4.2, based on mapping the pair of ellipses to a circle and an ellipse.

The curve ${\mathcal {H}}_{ij}$ is an intrinsically defined invariant of the pair of ellipses, no matter whether they are in contact or not. The points $\varvec{\gamma }_{ij}(t_i)$ and $\varvec{\gamma }_{ij}(t_j)$ are therefore properties of only the pair and it would be natural to use $\Vert \varvec{\gamma }_{ij}(t_i) - \varvec{\gamma }_{ij}(t_j) \Vert$ as a measure of closeness for pair of ellipses. Unfortunately, it is insufficient because it does not account for the relative size and orientations of the ellipses. The following numerical criterion will ensure a *small overlap*, in the sense of Theorem 7.

#### Definition 6

(*Ellipses in near-perfect contact*) Two ellipses ${\mathcal {E}}_i$, ${\mathcal {E}}_j \subset {\mathbb {R}}^2$ with non-penetrating CoM are said to be in near-perfect contact if

60 $$\begin{aligned} \frac{|t_i - t_j|}{\min _{k=i,j} \, 2 {\underline{\rho }}_k | \varvec{\gamma }_{ij}'(t_k) \cdot {\varvec{n}}_k(\varvec{\gamma }_{ij}(t_k)) | } \ll 1, \end{aligned}$$

where $\varvec{\gamma }_{ij}$, $t_i$, $t_j$ are defined as in Theorem 6, ${\varvec{n}}_k(\varvec{\gamma }_{ij}(t_k))$ is the outward normal unit vector to ${\mathcal {E}}_k$ at point $\varvec{\gamma }_{ij}(t_k)$ on ${\mathcal {E}}_k$, and ${\underline{\rho }}_k= b_k^2/a_k$, introduced in (18), is the smallest radius of curvature on ${\mathcal {E}}_k$, $k=i,j$.

This definition has geometric content that will become apparent in Sect. 3.6, particularly during the proof of the following theorem; see Sect. 3.7 for the proof.

#### Theorem 7

Consider two ellipses ${\mathcal {E}}_i$, ${\mathcal {E}}_j \subset {\mathbb {R}}^2$ in near-perfect contact, and let $t_i$ and $t_j$ be defined as in Theorem 6. Then, the intersection ${\mathcal {I}}_{ij} = {\mathcal {E}}_i \cap {\mathcal {E}}_j$ is one of the three options:

1. if $t_i < t_j$, then ${\mathcal {I}}_{ij} = \varnothing$, i.e., the ellipses are disjoint;
2. if $t_i = t_j$, then ${\mathcal {I}}_{ij}$ consists of a singleton, i.e., the ellipses are in perfect contact;
3. if $t_i > t_j$, then ${\mathcal {I}}_{ij}$ consists of two distinct points, i.e., the ellipses have small overlap.

With this theorem, we have shown that the constraint (60) is sufficient to avoid degenerate contacts between ellipses, that is those with three or four contact points. The proof of this theorem also leads to an explicit description of the intersection; see Corollary 12. We are now in a position to introduce the extensions of MDP and MPP.

#### Theorem 8

Let ${\mathcal {E}}_i$ and ${\mathcal {E}}_j$ be two ellipses in near-perfect contact. Then, there exists a unique pair of points $({\varvec{x}}_i,{\varvec{x}}_j) \in {\mathcal {E}}_i \times {\mathcal {E}}_j$ (known as MDP) which minimizes the Euclidean norm $\Vert {\varvec{x}}_i -{\varvec{x}}_j\Vert$, with the condition of opposite outward unit normal vectors ${\varvec{n}}_i({\varvec{x}}_i)$ and ${\varvec{n}}_j({\varvec{x}}_j)$ to ${\mathcal {E}}_i$ and ${\mathcal {E}}_j$, respectively, i.e.,

61 $$\begin{aligned} ({\varvec{x}}_i,{\varvec{x}}_j)  =  \mathop {\hbox {argmin}}\limits _{ \begin{array}{c} ({\hat{{\varvec{x}}}}_i,{\hat{{\varvec{x}}}}_j) \in {\mathcal {E}}_i \times {\mathcal {E}}_j \\ {\varvec{n}}_i({\hat{{\varvec{x}}}}_i) + {\varvec{n}}_j({\hat{{\varvec{x}}}}_j) = {\varvec{0}} \end{array}}  \Vert {\hat{{\varvec{x}}}}_i - {\hat{{\varvec{x}}}}_j\Vert  =  \mathop {\hbox {argmin}}\limits _{ \begin{array}{c} ({\hat{{\varvec{x}}}}_i,{\hat{{\varvec{x}}}}_j) \in {\mathcal {E}}_i \times {\mathcal {E}}_j \\ {\varvec{n}}_i({\hat{{\varvec{x}}}}_i) + {\varvec{n}}_j({\hat{{\varvec{x}}}}_j) = {\varvec{0}} \end{array} }  \frac{1}{2} \Vert {\hat{{\varvec{x}}}}_i - {\hat{{\varvec{x}}}}_j\Vert ^2.\nonumber \\ \end{aligned}$$

In light of Lemmas 3 and 5, we can conclude that the correct formulation for the MDP, in all configurations of pairs of ellipses that are small perturbations of perfect contact, is the doubly constrained minimization problem (61). The proof of this fundamental result requires Theorem 6, Theorem 7, and Corollary 12; hence, it is deferred to Sect. 3.7. There is a similar result below for the MPP which shows that the general formulation of the MPP for all pairs of ellipses requires a condition on opposing normals, even though this condition was optional for disjoint pairs of ellipses.

#### Theorem 9

Let ${\mathcal {E}}_i$ and ${\mathcal {E}}_j$ be two ellipses in near-perfect contact with global potentials $f_i$ and $f_j$, respectively. Then, there exists a unique pair of points $({\varvec{x}}_i,{\varvec{x}}_j) \in {\mathcal {E}}_i \times {\mathcal {E}}_j$ (known as MPP) satisfying the independent problems

62 $$\begin{aligned}&{\varvec{x}}_i  =  \mathop {\hbox {argmin}}\limits _{\begin{array}{c} {\varvec{x}}\in {\mathcal {E}}_i \\ {\varvec{n}}_i({\varvec{x}})+{\varvec{n}}_j({\varvec{x}}) = {\varvec{0}} \end{array}}  \Vert {\varvec{x}}- {\varvec{c}}_j \Vert _{{\mathcal {E}}_j}  =  \mathop {\hbox {argmin}}\limits _{\begin{array}{c} {\varvec{x}}\in {\mathcal {E}}_i \\ {\varvec{n}}_i({\varvec{x}})+{\varvec{n}}_j({\varvec{x}}) = {\varvec{0}} \end{array}}  f_j({\varvec{x}}), \end{aligned}$$

and

63 $$\begin{aligned}&{\varvec{x}}_j  =  \mathop {\hbox {argmin}}\limits _{\begin{array}{c} {\varvec{x}}\in {\mathcal {E}}_j \\ {\varvec{n}}_i({\varvec{x}})+{\varvec{n}}_j({\varvec{x}}) = {\varvec{0}} \end{array}}  \Vert {\varvec{x}}- {\varvec{c}}_i \Vert _{{\mathcal {E}}_i}  =  \mathop {\hbox {argmin}}\limits _{\begin{array}{c} {\varvec{x}}\in {\mathcal {E}}_j \\ {\varvec{n}}_i({\varvec{x}})+{\varvec{n}}_j({\varvec{x}}) = {\varvec{0}} \end{array}}  f_i({\varvec{x}}). \end{aligned}$$

#### Proof

Theorem 7 characterizing the intersection $E_i \cap E_j$ shows that ${\mathcal {H}}_{ij} \cap E_i \cap E_j$ only crosses ${\mathcal {E}}_i$ at $\varvec{\gamma }_{ij}(t_i)$ and ${\mathcal {E}}_j$ at $\varvec{\gamma }_{ij}(t_j)$. If ${\varvec{x}}\in {\mathcal {E}}_i \cap {\mathcal {H}}_{ij}$ does not belong to the intersection, then it must belong outside of $E_j$, hence

$$\begin{aligned} f_j({\varvec{x}}) > 0 \ge f_j({\varvec{x}}_i). \end{aligned}$$

This shows that ${\varvec{x}}_i = \varvec{\gamma }_{ij}(t_i)$ must be the unique minimum of Problem (62). A similar argument applies to $\varvec{\gamma }_{ij}(t_j) \in {\mathcal {E}}_j \cap {\mathcal {H}}_{ij}$, thus completing the proof. $\square$

### Contact potentials of Perram and Wertheim

The formulation of the MDP and the MPP has many similarities to the theory of *contact potentials* of Perram and Wertheim [41]. Although the purpose of contact potentials is only to identify contacts, and as such does not estimate the separation/penetration distance between particles, the mathematical similarities warrant a brief description.

Perram and Wertheim developed functions, called contact potentials, whose sign could efficiently compute if two convex smooth particles were disjoint ($>0$), overlapping ($<0$), or tangent ($=0$). This technique, extending earlier work of Vieillard-Baron [47], was widely adopted by statistical physicists studying phase transitions in specific models [9, 40]. Today, simulations in statistical physics have also adopted even more efficient approximations to contact potentials, such as the Gay–Berne potential [16], for the generation of dense particle assemblies and fast computation of their thermodynamic properties. We note that contact potentials are equally well defined in both two and three space dimensions.

Consider two convex smooth particles, described by indexes *i* and *j*, whose interiors are defined by $\zeta _i({\varvec{x}}) < 0$ and $\zeta _j({\varvec{x}}) < 0$, respectively, and whose boundaries occur at $\zeta _i({\varvec{x}}) = 0$ and $\zeta _j({\varvec{x}}) = 0$, respectively. The global potentials of two ellipses satisfy these properties, but technically speaking the contact potentials do not require $\zeta _i$ and $\zeta _j$ to be of this form. The contact potential $\zeta _{ij}$ for the two particles is the quantity

$$\begin{aligned} \zeta _{ij} = \max _{0 \le \lambda \le 1} \min _{{\varvec{z}}} \lambda \zeta _i({\varvec{z}}) + (1-\lambda )\zeta _j({\varvec{z}}), \end{aligned}$$

and so it is clearly also defined as an optimization problem, just like the MDP and the MPP. For each fixed value of $\lambda$, the minimum with respect to ${\varvec{z}}$ occurs at the critical point, i.e., the solution to

64 $$\begin{aligned} 0&= \nabla _{{\varvec{z}}} \big ( \lambda \zeta _i({\varvec{z}}) + (1-\lambda )\zeta _j({\varvec{z}}) \big ) \nonumber \\&= \lambda \nabla _{{\varvec{z}}} \zeta _i({\varvec{z}}) + (1-\lambda ) \nabla _{{\varvec{z}}} \zeta _j({\varvec{z}}). \end{aligned}$$

This shows that for all $\lambda \in [0,1]$, the critical point ${\varvec{z}}$ belongs to the co-gradient locus. Similarly, the maximum with respect to $\lambda$ occurs at a value of ${\varvec{z}} = {\varvec{z}}(\lambda )$ satisfying

65 $$\begin{aligned} \zeta _i({\varvec{z}}) = \zeta _j({\varvec{z}}). \end{aligned}$$

As Perram and Wertheim explained in their original paper, $\zeta _{ij}$ corresponds to the value of

$$\begin{aligned} \lambda \zeta _i({\varvec{x}}) + (1-\lambda )\zeta _j({\varvec{x}}) \end{aligned}$$

at the point ${\varvec{x}}\in {\mathcal {H}}_{ij}$ where equal scalings of the original surfaces, in the sense of (65), are in perfect contact, according to Definition 4. More details about the geometry of contact potentials can be found in an article of Paramonov and Yaliraki [39].

The development of the theory of contact potentials is largely expressed in terms of explicit algebraic constraints, and so most questions surrounding existence and uniqueness are rather easy to deal with. In fact, many of the key results were already demonstrated in an appendix to the original paper by Vieillard-Baron [47].

### The co-gradient locus

The analysis of the previous sections has highlighted the key role that the co-gradient locus, see Definition 5, plays in characterizing intersections for ellipses in near-perfect contact, and even in solving for the MDP and the MPP. The purpose of this section is to make a thorough study of ${\mathcal {H}}_{ij}$ in ${\mathbb {R}}^2$, including when the hyperbola degenerates. We begin by offering a few equivalent definitions of the co-gradient locus that will be useful later on.

In 2-D, the cross-product defining the co-gradient function is interpreted as a cross-product in 3-D between the gradients in the 2-D plane. It therefore results in a 3-D vector with only one nonzero component along the *z*-axis, which is given by the scalar function

66 $$\begin{aligned} h({\varvec{x}})&:= {\text {det}} \begin{bmatrix} \nabla f_i({\varvec{x}})&\nabla f_j({\varvec{x}}) \end{bmatrix} \nonumber \\&\,= \partial _x f_i({\varvec{x}}) \partial _y f_j({\varvec{x}}) -\, \partial _y f_i({\varvec{x}}) \partial _x f_j({\varvec{x}}). \end{aligned}$$

We shall consider this definition of the co-gradient function when dealing with ellipses rather than the vector-valued ${\varvec{h}}$ in (56). Introducing the anti-symmetric matrix,

67 $$\begin{aligned} A = \begin{bmatrix} ~ 0 &{} 1 \\ -1 &{} 0 \end{bmatrix}, \end{aligned}$$

the co-gradient function can also be written as

68 $$\begin{aligned} h({\varvec{x}}) = (\nabla f_i({\varvec{x}}))^T A \, \nabla f_j({\varvec{x}}) = 4 ({\varvec{x}}-{\varvec{c}}_i)^T {\mathcal {Q}}_i A {\mathcal {Q}}_j ({\varvec{x}}-{\varvec{c}}_j),\nonumber \\ \end{aligned}$$

where we have used the fact that ${\mathcal {Q}}_i$ is symmetric. We immediately observe that *h* is a quadratic polynomial in ${\varvec{x}}$ and that the centers ${\varvec{c}}_i$ and ${\varvec{c}}_j$ of the ellipses belong to ${\mathcal {H}}_{ij}$. If the product ${\mathcal {Q}}_i A {\mathcal {Q}}_j$ was symmetric, then the determinant of the product could immediately tell us the geometry of the co-gradient locus. Unfortunately, the detailed characterization of ${\mathcal {H}}_{ij}$ presented in Theorem 6 requires significantly more work.

A second characterization can be made by normalizing the gradients in (56). Recalling the definition of the unit normal vectors (16), i.e.,

69 $$\begin{aligned} {\varvec{n}}_k({\varvec{x}}) = \frac{\nabla f_k({\varvec{x}})}{\Vert \nabla f_k({\varvec{x}}) \Vert }, \quad k=i,j, \end{aligned}$$

then the *normalized co-gradient function* is

70 $$\begin{aligned} {\hat{{\varvec{h}}}}({\varvec{x}}) = {\varvec{n}}_i({\varvec{x}}) \times {\varvec{n}}_j({\varvec{x}}). \end{aligned}$$

The scalar component in the *z*-direction of ${\hat{{\varvec{h}}}}({\varvec{x}}) \in {\mathbb {R}}^3$ is equal to $\sin \eta _{ij}({\varvec{x}})$ where $\eta _{ij}({\varvec{x}})$ is the angle between ${\varvec{n}}_i$ and ${\varvec{n}}_j$, well-defined modulo $2\pi$. Mimicking the definition (68) of the *z*-component of ${\hat{{\varvec{h}}}}$, the co-gradient locus in 2-D is simply the set of roots of

71 $$\begin{aligned} {\hat{h}}({\varvec{x}}) = \sin \eta _{ij}({\varvec{x}}). \end{aligned}$$

The angle $\eta _{ij}({\varvec{x}})$ can also be defined by identifying ${\varvec{n}}_i$ and ${\varvec{n}}_j$ with unitary complex numbers, so that, using complex multiplication

72 $$\begin{aligned} {\varvec{n}}_j({\varvec{x}}) = e^{i \eta _{ij}({\varvec{x}})} {\varvec{n}}_i({\varvec{x}}). \end{aligned}$$

The roots of ${\hat{h}}$ correspond to $\eta _{ij}({\varvec{x}}) = m \pi$, $m \in {\mathbb {Z}}$. We note that at infinity, relation (72) can be rewritten as ${\mathcal {N}}_j({\varvec{w}}) = e^{i \eta _{ij}({\varvec{w}})} {\mathcal {N}}_i({\varvec{w}})$, where ${\mathcal {N}}_i$ and ${\mathcal {N}}_j$ are the maps of Lemma 1, that is the angle $\eta _{ij} = \eta _{ij}({\varvec{w}})$ only depends on the direction ${\varvec{w}}$. Eventually, we will show that the angle $\eta _{ij}$ must belong to $]-\pi ,\pi [$, and hence is well defined. We now proceed with the proof of Theorem 6.

#### Proof

The proof will show that the co-gradient function *h*, which is a quadratic function according to (68), possesses four roots at infinity. This will imply that the roots ${\mathcal {H}}_{ij} \subset {\mathbb {R}}^2$ form a hyperbola because ellipses, parabolas, and hyperbolas possess, respectively, 0, 2, and 4 roots at infinity on the projective sphere. Afterward, we will argue that a single branch of the hyperbola must cross both centers of the ellipses, thereby justifying the existence of the parameterization.

The majority of the analysis will be performed on a pair of ellipses in a *generic* configuration but this will require us to begin the proof with a lengthy study of different *degenerate* configurations. Bivariate quadratic polynomials have roots that can degenerate to either of the following configurations: two intersecting lines, two parallel lines, a line with a second line at infinity, two coincident lines, or a single point. The last option will never occur because *h* already vanishes at the centers ${\varvec{c}}_i \ne {\varvec{c}}_j$. The analysis below will show that ${\mathcal {H}}_{ij}$ always contains at least four points at infinity and hence cannot be formed of two parallel lines or two coincident lines.

The first configuration we study assumes that ${\varvec{c}}_j$ belongs to the axis $\varvec{\xi }_i$ and that the principal axes of ${\mathcal {E}}_j$ are aligned with those of ${\mathcal {E}}_i$, although the argument will also work if ${\varvec{c}}_j$ belongs to the axes $\varvec{\eta }_i$ and $\varvec{\eta }_i = \varvec{\xi }_j$. Under these conditions, for all $t,s \in {\mathbb {R}}$, Property (ii) of Lemma 1 shows that

$$\begin{aligned} {\varvec{n}}_i({\varvec{c}}_i+t \varvec{\xi }_i) = {\text {sign}}(t) \varvec{\xi }_i = \pm \varvec{\xi }_j = \pm {\varvec{n}}_j( {\varvec{c}}_j + s \varvec{\xi }_j). \end{aligned}$$

Hence, every point of the axis ${\varvec{c}}_i+t \varvec{\xi }_i$ belongs to ${\mathcal {H}}_{ij}$. Furthermore, the fact that the axes are aligned and Property (iv)–(a) implies that

$$\begin{aligned} {\mathcal {N}}_i(\varvec{\eta }_i) = {\mathcal {N}}_j(\varvec{\eta }_j), \end{aligned}$$

or, in other words, *h* possesses roots at infinity in the direction ${\varvec{w}}= \pm \varvec{\eta }_i = \pm \varvec{\eta }_j$. In this configuration, the co-gradient locus has four roots at infinity and contains a line and hence is either two intersecting lines, or a line with a second line at infinity. If the two ellipses have the same aspect ratio, then relation (31) of Lemma 1 states that ${\mathcal {N}}_i({\varvec{w}}) = {\mathcal {N}}_j({\varvec{w}}),$ for all ${\varvec{w}}\in S^1$. In other words, ${\mathcal {H}}_{ij}$ contains the line at infinity. On the other hand, when the aspect ratios are different, the same relation shows that ${\mathcal {N}}_i \ne {\mathcal {N}}_j$ and thus the co-gradient locus must be formed of two intersecting lines.

**Fig. 6:**
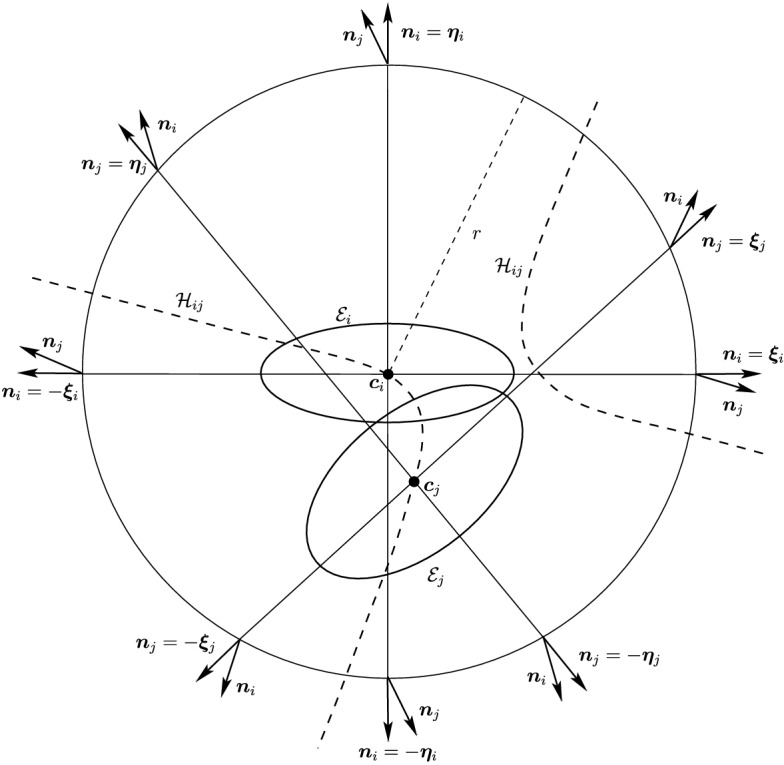
Illustration of the proof of Theorem 6. The ellipses ${\mathcal {E}}_i$ and ${\mathcal {E}}_j$ are in a configuration with ${\varvec{c}}_i$ at the origin and ${\mathcal {E}}_i$ is aligned with horizontal axis. The circle with radius *r* is large enough that all normals are external on the ellipses, i.e., ${\varvec{n}}_i \cdot {\varvec{n}}_j \ge 0$

Consider now the case where ${\varvec{c}}_j$ does not belong to either principal axis of ${\mathcal {E}}_i$ but continue to assume that the principal axes of both ellipses are aligned, say $\varvec{\xi }_i = {\varvec{x}}_j$ and $\varvec{\eta }_i = \varvec{\eta }_j$. Property (iv)–(a) of Lemma 1 tells us that

$$\begin{aligned} {\mathcal {N}}_i( \pm \varvec{\xi }_i) = {\mathcal {N}}_j(\pm \varvec{\xi }_j) \, , \qquad {\mathcal {N}}_i( \pm \varvec{\eta }_i) = {\mathcal {N}}_j(\pm \varvec{\eta }_j), \end{aligned}$$

and hence there are at least four roots at infinity. Again, if the ellipses have the same aspect ratio, then ${\mathcal {N}}_i = {\mathcal {N}}_j$ and the co-gradient locus is degenerate and contains a line at infinity. Otherwise, the co-gradient locus is a hyperbola, which or may not be degenerate; see Corollary 10 for more on this issue.

The general configuration on which we will focus the remainder of our attention assumes that the principal axes of the two ellipses are not aligned, whether or not either center belongs to the axis of its brethren. In this case, we observe that the straight line ${\varvec{c}}_j+t\varvec{\xi }_j$, $t\in {\mathbb {R}}$, for |*t*| sufficiently large, belongs to two opposing quadrants: either $[\varvec{\xi }_i, \varvec{\eta }_i]$ and $[-\varvec{\xi }_i, -\varvec{\eta }_i]$ or $[\varvec{\eta }_i, -\varvec{\xi }_i]$ and $[-\varvec{\eta }_i,\varvec{\xi }_i]$. When the second case occurs, the line $t \varvec{\xi }_i$ crosses the opposing quadrants $[\varvec{\xi }_j, \varvec{\eta }_j]$ and $[-\varvec{\xi }_j, -\varvec{\eta }_j]$. Hence, the second case can be brought into the first configuration by translating ${\mathcal {E}}_j$ to the origin and exchanging the indices *i* and *j*. Figure 6 illustrates this configuration, after assuming a translation and a rotation sending ${\varvec{c}}_i$ to the origin and the axes of ${\mathcal {E}}_i$ over to the usual Cartesian axes.

The map (30) associates with each direction ${\varvec{w}}\in S^1$, the unique normals ${\mathcal {N}}_i({\varvec{w}})$, ${\mathcal {N}}_j({\varvec{w}})$ on the line at infinity. We may then measure the angle $\eta _{ij} = \eta _{ij}({\varvec{w}})$ between the normals at infinity using the relation (72), rewritten here using complex multiplication as

73 $$\begin{aligned} {\mathcal {N}}_j( {\varvec{w}}) = e^{i \eta _{ij}({\varvec{w}})} {\mathcal {N}}_i({\varvec{w}}), \end{aligned}$$

In this last identity, positive or negative angles correspond, respectively, to a counterclockwise or clockwise rotation when rotating ${\mathcal {N}}_i$ toward ${\mathcal {N}}_j$. It is essential to observe that the angle $\eta _{ij}$ is well defined within $]-\pi ,\pi [$ because Property (iv)–(c) of Lemma 1 states that the rotation $\theta$ from ${\varvec{w}}$ to either ${\mathcal {N}}_i({\varvec{w}})$ or ${\mathcal {N}}_j({\varvec{w}})$ is strictly bounded $|\theta |<\pi /2$, and hence the rotation from ${\mathcal {N}}_i$ to ${\mathcal {N}}_j$ must be by an angle $\eta _{ij}$ strictly less than $\pi$ in absolute value. As ${\varvec{w}}$ moves counterclockwise around $S^1$ starting at $\varvec{\xi }_i$, the configuration we have chosen, as shown in Fig. 6, implies that we will encounter in order the directions $\varvec{\xi }_i$, $\varvec{\xi }_j$, $\varvec{\eta }_i$, $\varvec{\eta }_j$, $-\varvec{\xi }_i$, $-\varvec{\xi }_j$, $-\varvec{\eta }_i$, $-\varvec{\eta }_j$, $\varvec{\xi }_i$. We will focus on demonstrating that $\eta _{ij}$ possesses a root inside the arc $[\varvec{\xi }_j,\varvec{\eta }_i] \subset S^1$, but similar arguments will show that there are at least three other roots, one in each of the three arcs $[\varvec{\eta }_j,-\varvec{\xi }_i]$, $[-\varvec{\xi }_j,-\varvec{\eta }_i]$, and $[-\varvec{\eta }_j,-\varvec{\xi }_i]$. Each root of $\eta _{ij}$ corresponds to equal normals and hence to a root of *h*, thereby demonstrating that ${\mathcal {H}}_{ij}$ is a hyperbola.

Consider the principal axis for ${\mathcal {E}}_j$ centered at ${\varvec{c}}_j \ne {\varvec{c}}_i$, i.e., take ${\varvec{w}}= \varvec{\xi }_j$. Then, estimate (32) and Property (iv)–(d) of Lemma 1 show that the unit normal ${\varvec{n}}_j({\varvec{c}}_i +r\varvec{\xi }_j)$ converges to ${\mathcal {N}}_j(\varvec{\xi }_j)= \varvec{\xi }_j$ as the radius *r* increases. Furthermore, if $\varvec{\xi }_j = e^{i \sigma _j} \varvec{\xi }_i$ for $\sigma _j \in [0,\pi /2[$, then Property *iv)-(c)* states that

$$\begin{aligned} {\mathcal {N}}_i( \varvec{\xi }_j) = e^{i \theta _i} \varvec{\xi }_j, \end{aligned}$$

with

$$\begin{aligned} \tan (\theta _i+\sigma _j) = \left( \frac{a_i}{b_i}\right) ^2 \tan \sigma _j. \end{aligned}$$

Given that $\tan \sigma _j > 0$ and $a_i/b_i> 1$, we find that $\theta _i>0$ and therefore must belong to $[0,\pi /2[$. Using these facts and the estimate (32), we find

$$\begin{aligned} {\mathcal {N}}_i(\varvec{\xi }_j) = e^{i \theta _i} \varvec{\xi }_j = e^{i \theta _i} {\mathcal {N}}_j(\varvec{\xi }_j) = e^{i \theta _i} e^{i \eta _{ij}} {\mathcal {N}}_i(\varvec{\xi }_j) . \end{aligned}$$

This implies that $\eta _{ij}(\varvec{\xi }_j) = - \theta _i \le 0$. On the other hand, we have ${\mathcal {N}}_i( \varvec{\eta }_i) = \varvec{\eta }_i$, while the inequality (31) states that

$$\begin{aligned} {\mathcal {N}}_j(\varvec{\eta }_i) = e^{i \theta _j } \varvec{\eta }_i = e^{i \theta _j }{\mathcal {N}}_i( \varvec{\eta }_i ) = e^{i \theta _j } e^{-i \eta _{ij}} {\mathcal {N}}_j(\varvec{\eta }_i), \end{aligned}$$

with $\theta _j \in [0,\pi /2[$ following the previous argument. Hence, $\eta _{ij}(\varvec{\eta }_i) = \theta _j \ge 0$. Since $\eta _{ij}({\varvec{w}}) \in \ ]-\pi ,\pi [$ and changes sign as the direction ${\varvec{w}}$ varies from $\varvec{\xi }_j$ to $\varvec{\eta }_i$, there exists a direction ${\varvec{w}}$ in $]\varvec{\xi }_j,\varvec{\eta }_i[$ where $\eta _{ij}$ vanishes and both normals are equal. The same argument is applied to the three other intervals, which shows that there are four directions at infinity where the normals coincide, i.e., the co-gradient function *h* has four roots at infinity.

To conclude the proof, we need to show that a single connected branch of the hyperbola crosses the centers of the two ellipses. It is easy to verify that ${\varvec{c}}_i$ and ${\varvec{c}}_j$ belong to ${\mathcal {H}}_{ij}$ by substituting directly into (68). Consider the function $\alpha ({\varvec{x}}) = {\varvec{n}}_i({\varvec{x}}) \cdot {\varvec{n}}_j({\varvec{x}})$ for all ${\varvec{x}}$ belonging to the smooth affine variety ${\mathcal {H}}_{ij}$. By construction, $\alpha$ only takes on the values $\pm 1$ and is ill-defined at both centers. Since $\alpha$ is continuous on ${\mathcal {H}}_{ij} {\setminus }\{ {\varvec{c}}_i,{\varvec{c}}_j\}$, it must have constant values over each connected component of ${\mathcal {H}}_{ij} {\setminus }\{ {\varvec{c}}_i,{\varvec{c}}_j\}$. Furthermore, for points ${\varvec{x}}$ far from the centers, the normals are related by ${\varvec{n}}_j = e^{i \eta _{ij}} {\varvec{n}}_i$ where $\eta _{ij} \in \ ]-\pi ,\pi [$ and therefore the dot product must take positive values, i.e., $\alpha ({\varvec{x}}) = +1$. If the points ${\varvec{c}}_i$ and ${\varvec{c}}_j$ belong to different branches of the hyperbola ${\mathcal {H}}_{ij}$, then each connected component of ${\mathcal {H}}_{ij} {\setminus }\{ {\varvec{c}}_i,{\varvec{c}}_j\}$ reaches infinity and $\alpha$ must be equal to $+1$ everywhere. Yet, in the neighborhood of a center, say ${\varvec{c}}_i$, the normal ${\varvec{n}}_j$ varies smoothly, and along the tangent to ${\mathcal {H}}_{ij}$ at ${\varvec{c}}_i$, property (ii) of Lemma 1 states that the normals ${\varvec{n}}_i$ are equal and opposite on both sides of ${\varvec{c}}_i$. Hence, the function $\alpha$ must take opposite values, i.e., $+1$ and $-1$, when passing through the center ${\varvec{c}}_i$ along the branch of the hyperbola. This would contradict the conclusion that $\alpha$ is identically $+1$ on ${\mathcal {H}}_{ij} {\setminus }\{ {\varvec{c}}_i,{\varvec{c}}_j\}$, following from the hypothesis that ${\varvec{c}}_i$ and ${\varvec{c}}_j$ belong to different branches of ${\mathcal {H}}_{ij}$. The existence of the parameterization $\gamma _{ij}$ is a trivial consequence of the fact that both centers belong to the same branch of the hyperbola. The fact that ${\mathcal {H}}_{ij}$ is a hyperbola implies that the portion $\varvec{\gamma }_{ij}([0,1]) \subset {\mathcal {H}}_{ij}$ can only cross each ellipse ${\mathcal {E}}_k$, $k=i,j$, once when connecting both centers, thereby demonstrating the uniqueness of $t_k$, $k=i,j$. $\square$

The next result is included for the sake of completeness because it clearly shows that properties of the pair of ellipses are reflected in the geometry of ${\mathcal {H}}_{ij}$. In this sense, it indicates that additional study of the co-gradient locus might prove worthwhile, particularly the study of the second branch. Nevertheless, this result will only be used in the construction of an example later in this section.

#### Corollary 10

Two ellipses ${\mathcal {E}}_i$, ${\mathcal {E}}_j \subset {\mathbb {R}}^2$ with non-penetrating CoM are parameterized by an open subset of $\big ( {\mathbb {R}}^2 \times {\text {SPD}}_2({\mathbb {R}})\big )^2$, a manifold of dimension 10. The hyperbola ${\mathcal {H}}_{ij}$ only degenerates for a subset of codimension 3. When the hyperbola ${\mathcal {H}}_{ij}$ degenerates, it can only be either two intersecting lines, or a line with a second line at infinity. The line at infinity can only appear if the principal axes of both ellipses are aligned and have the same aspect ratio.

#### Proof

The analysis in Lemma 6 has already shown that the degenerate quadratic cannot be formed of a single point (${\varvec{c}}_i, {\varvec{c}}_j \in {\mathcal {H}}_{ij}$), two parallel lines or two coincident lines (${\mathcal {H}}_{ij}$ at infinity always has $\ge 4$ points). The only two remaining possibilities are those mentioned in the statement of the corollary. To complete the proof, we will first deduce the conditions required for ${\mathcal {H}}_{ij}$ to contain a line at infinity. Afterward, we will identify the conditions under which the co-gradient locus contains at least one line.

If the ellipses have aligned axes, then relation (31) clearly implies that the co-gradient locus possesses a line at infinity if and only if the aspect ratios are the same. Suppose now that the axes are not aligned but that the co-gradient locus still possesses a line at infinity. We will show that these hypotheses lead to a contradiction. Considering the axis directions as elements of $S^1$, suppose that $\varvec{\xi }_j \in \ ]\varvec{\xi }_i, \varvec{\eta }_i]$, although similar arguments will apply if it belongs to $]\varvec{\eta }_i, -\varvec{\xi }_i[$. Given that ${\mathcal {H}}_{ij}$ possesses a line at infinity, we have that ${\mathcal {N}}_i = {\mathcal {N}}_j$ and in particular

74 $$\begin{aligned} {\mathcal {N}}_i(\varvec{\xi }_j) = {\mathcal {N}}_j(\varvec{\xi }_j) = \varvec{\xi }_j , \qquad {\mathcal {N}}_j(\varvec{\xi }_i) = {\mathcal {N}}_i(\varvec{\xi }_i) = \varvec{\xi }_i . \end{aligned}$$

The map ${\mathcal {N}}_i$ cannot be the identity map, or else both ellipses would be circles and hence have aligned axes. Hence, for ${\varvec{w}}= e^{i \sigma } \varvec{\xi }_i$ with $\sigma \in \ ]0, \pi /2[$, there exists $\theta \in \ ]0,\pi /2[$ satisfying

$$\begin{aligned} {\mathcal {N}}(e^{i \sigma }\varvec{\xi }_i) = e^{i (\theta +\sigma )} \varvec{\xi }_i , \quad \text {with } \tan (\theta + \sigma ) = (a_i/b_i)^2 \tan \sigma . \end{aligned}$$

The first relation in (74) for $\varvec{\xi }_j = e^{i\sigma } \varvec{\xi }_i$ gives us

$$\begin{aligned} \varvec{\xi }_j ={\mathcal {N}}_i( \varvec{\xi }_j) = {\mathcal {N}}_i( e^{i \sigma } \varvec{\xi }_i) = e^{i(\theta + \sigma )} \varvec{\xi }_i = e^{i \theta } \varvec{\xi }_j , \end{aligned}$$

that is $\theta = 0$. The implicit relationship (31) between $\theta$ and $\sigma$ shows that $\theta$ can vanish only if $a_i/b_i = 1$. Repeating the same argument with the second relation in (74) proves that $a_j/b_j = 1$. In conclusion, if the axes are not aligned but ${\mathcal {H}}_{ij}$ possesses a line at infinity, then both ellipses are circles.

We now attempt to determine the most general conditions under which the co-gradient locus can degenerate to a pair of intersecting lines. If we examine the values of the continuous function $\alpha ({\varvec{x}}) = {\varvec{n}}_i({\varvec{x}}) \cdot {\varvec{n}}_j({\varvec{x}})$ along ${\mathcal {H}}_{ij}$, we notice that it only take on the values $\pm 1$. During the proof of Theorem 6, we observed that $\alpha$ changes sign as we cross either center but that $\alpha$ on co-gradient locus always took the value $\alpha ({\varvec{x}}) = 1$ when $\Vert {\varvec{x}}\Vert$ was sufficiently large. This implies that if ${\mathcal {H}}_{ij}$ degenerates to two intersecting lines, then the two centers cannot belong to different lines, and when they do, the second line cannot cross the first between the two centers.

Consider the line connecting both centers, which can be parameterized as ${\varvec{c}}_i + t {\varvec{w}}$, for $t \in \ ]0,\tau [$, with ${\varvec{w}}= ({\varvec{c}}_j-{\varvec{c}}_i)/\Vert {\varvec{c}}_j-{\varvec{c}}_i\Vert$ so that $\tau = \Vert {\varvec{c}}_j-{\varvec{c}}_i\Vert$. Along this segment, Property (iii) of Lemma 1 states that the normals are constant. Using Properties (ii) and (iv)–(c) only on the portion of the line between both centers, we compute

$$\begin{aligned} \begin{aligned} {\varvec{n}}_i({\varvec{c}}_i + t {\varvec{w}})&= {\mathcal {N}}_i({\varvec{w}}) = e^{i \theta _i} {\varvec{w}}, \\ {\varvec{n}}_j({\varvec{c}}_i + t {\varvec{w}})&= {\varvec{n}}_j({\varvec{c}}_j + (t - \tau ) {\varvec{w}}) = {\mathcal {N}}_j(-{\varvec{w}}) = - e^{i \theta _j} {\varvec{w}}\\&= - e^{i \theta _j} e^{- i \theta _i} {\mathcal {N}}_i( {\varvec{w}}) . \end{aligned} \end{aligned}$$

In the previous identities, the angles were functions $\theta _k = \theta _k(\sigma _k)$ for ${\varvec{w}}= e^{i \sigma _k} \varvec{\xi }_k$, $k=i,j$, according to the implicit relations

75 $$\begin{aligned} \tan \big ( \theta _k + \sigma _{k} \big ) = \left( \frac{a_k}{b_k}\right) ^2 \tan \sigma _{k}, \qquad k=i,j. \end{aligned}$$

Since ${\mathcal {N}}_i({\varvec{w}}) = - {\mathcal {N}}_j(-{\varvec{w}})$, we conclude that

76 $$\begin{aligned} \theta _i = \theta _j . \end{aligned}$$

Each ellipse is parameterized in a space of dimension 5, two for each center ${\varvec{c}}_k$ and three for each matrix ${\mathcal {Q}}_k$. These 10 parameters determine $a_k$, $b_k$, $\theta _{k,0}$, $\sigma _k$, and $\theta _k$; hence, the three identities (76) and (75) determine a subspace of co-dimension 3 where ${\mathcal {H}}_{ij}$ is degenerate. In other words, Sard’s theorem [33] states that ${\mathcal {H}}_{ij}$ degenerates only over a subset of measure zero in the space of parameters for ${\mathcal {E}}_i$ and ${\mathcal {E}}_j$. $\square$

#### Remark 5

We will present a degenerate co-gradient locus formed of two intersecting lines but for a pair of ellipses whose principal axes are not aligned. This example is instructive in that it goes beyond the degenerate examples identified during the proof of Theorem 6 and demonstrates that the conditions (75) and (76) are non-empty. Furthermore, this example is new to the literature and could be used for code verification.

Consider the ellipse ${\mathcal {E}}_i$ described by its geometric potential

77 $$\begin{aligned} f_i({\varvec{x}}) = \frac{x^2}{3} + y^2 - 1 . \end{aligned}$$

The point ${\varvec{x}}_i = [\sqrt{3/2}, 1/\sqrt{2}]^T$ belongs to ${\mathcal {E}}_i$ and the line $\ell$ passing through the origin and ${\varvec{x}}_i$ forms an angle of $\pi /6$ with the horizontal axis because

$$\begin{aligned} \tan (\pi /6) = 1/\sqrt{3} . \end{aligned}$$

Using Formula (16) shows that the gradient to ${\mathcal {E}}_i$ at ${\varvec{x}}_i$ is given by

$$\begin{aligned} \nabla f_i( {\varvec{x}}_i) = [\sqrt{3/2},\sqrt{2}]^T . \end{aligned}$$

A simple calculation shows that the angle measured counterclockwise between ${\varvec{x}}_i$ and $\nabla f_i({\varvec{x}}_i)$ is also $\pi /6$. This conforms with relation (31),

$$\begin{aligned} \tan \left( \frac{\pi }{6} + \frac{\pi }{6} \right) = \big ( \sqrt{3} \big )^2 \tan \left( \frac{\pi }{6} \right) . \end{aligned}$$

Our objective is to construct an ellipse ${\mathcal {E}}_j$ whose center ${\varvec{c}}_j$ belongs to the line $\ell$ and whose normal ${\mathcal {N}}_j$ in the direction $- {\varvec{x}}_i$ is opposite to the normal ${\mathcal {N}}_i$ in the direction ${\varvec{x}}_i$. To keep this as simple as possible, we will describe the ellipse in its local coordinates

78 $$\begin{aligned} {\widehat{f}}_j({\varvec{x}}) = \frac{\xi ^2}{2+\sqrt{3}} + \eta ^2 - 1 , \end{aligned}$$

and we introduce the point

$$\begin{aligned} {\widehat{{\varvec{x}}}}_j = \left[ - \sqrt{\frac{2+\sqrt{3}}{3+\sqrt{3}}}, - \sqrt{\frac{2+\sqrt{3}}{3+\sqrt{3}}}\right] ^T , \end{aligned}$$

on the ellipse. With respect to the $\varvec{\xi }_j$ axis and measured counterclockwise, the line through the origin and ${\widehat{{\varvec{x}}}}_j$ forms an angle of $5 \pi /4$. The gradient to ${\mathcal {E}}_j$ gradient at ${\widehat{{\varvec{x}}}}_j$ is

$$\begin{aligned} \nabla {\widehat{f}}_j( {\widehat{{\varvec{x}}}}_j) = 2 \left[ - \frac{1}{\sqrt{(2+\sqrt{3})(3+\sqrt{3})}}, - \sqrt{\frac{2+\sqrt{3}}{3+\sqrt{3}}}\right] ^T , \end{aligned}$$

and it is easy to see that it forms an angle of $\pi /6$ with ${\widehat{{\varvec{x}}}}_j$. Hence, relation (31) is again satisfied

$$\begin{aligned} \tan \left( \frac{\pi }{6} + \frac{5\pi }{4} \right) = \big ( 2+\sqrt{2} \big ) \tan \left( \frac{5\pi }{4} \right) , \end{aligned}$$

using the fact that $\tan ( 5\pi /12) = 2 + \sqrt{3}$.

The ellipse ${\mathcal {E}}_j$ can be translated so that ${\varvec{c}}_j$ is located along the line $\ell$, say in the first quadrant, and then it can be rotated by $- \pi /12 = \pi /6 - \pi /4$ so that the point ${\widehat{{\varvec{x}}}}_j$ also falls on the line. Afterward, the axis of the two ellipses will no longer be aligned, but their normals will be opposite everywhere on the line between the two centers. The line $\ell$ will therefore be a part of the co-gradient locus. We conclude by remarking that this example clearly satisfies the three conditions for degeneracy of Corollary 10, that is formulas (75) and (76).

### Characterizing near-perfect contacts

This section will demonstrate the existence and uniqueness of the MDP for pairs of ellipses in near-perfect contact, see Theorem 8, after proving the characterization of near-perfect contact in Theorem 7. The proof of Theorem 7 includes an explicit characterization of the intersection, summarized in Corollary 12, which is very useful when studying the MDP. The results in this section are again stated in ${\mathbb {R}}^2$, but the extensions to ${\mathbb {R}}^3$ are rather straightforward.

Rather than attempting directly to study the intersection of two ellipses, we begin with a first-order approximation with two circles. Once equipped with explicit estimates with pairs of overlapping circles, we will eventually argue that the intersection of pairs of ellipses can be approximated by intersections of pairs of circles. Although the detailed results of this section were not all necessary for the study of the MPP, as the proof of Theorem 9 shows, they are necessary for MDP. In fact, we expect that the tools described in this section will find other uses.

#### Lemma 11

Consider a pair of overlapping circles ${\mathcal {C}}_i$ and ${\mathcal {C}}_j$ with non-penetrating CoM. If $D_i$ and $D_j$ are the closed discs bounded by the respective circles ${\mathcal {C}}_i$ and ${\mathcal {C}}_j$, then the intersection is the union of two closed domains $A_{ij}^+$ and $A_{ij}^-$,

$$\begin{aligned} D_i \cap D_j = A_{ij}^+ \cup A_{ij}^- , \end{aligned}$$

whose intersection is $A_{ij}^+ \cap A_{ij}^- = {\mathcal {H}}_{ij} \cap (D_i \cap D_j)$. Both $A_{ij}^+$ and $A_{ij}^-$ are diffeomorphic to a triangle.

#### Proof

We begin by simplifying the geometry through a translation and a rotation sending the center ${\varvec{c}}_i$ to the origin, and the second center ${\varvec{c}}_j$ to $(c_j,0)$ along the positive *x*-axis. Corollary 10 states that the co-gradient locus is the *x*-axis with a second branch at infinity. The potential for the circles is

$$\begin{aligned} f_i(x,y) =&\Big ( \frac{x}{r_i} \Big )^2 + \Big ( \frac{y}{r_i} \Big )^2 - 1 , \\ f_j(x,y) =&\Big ( \frac{x-c_j}{r_j} \Big )^2 + \Big ( \frac{y}{r_j} \Big )^2 - 1 , \end{aligned}$$

and the normalized distance to the centers is

$$\begin{aligned} \lambda _i(x,y) =&\Big ( f_i(x,y) +1 \Big )^{1/2} , \\ \lambda _j(x,y) =&\Big ( f_j(x,y) + 1 \Big )^{1/2} . \end{aligned}$$

We now proceed to detail the intersection between the two discs. The circles are overlapping, hence $c_j < r_i+r_j$. The condition of non-penetrating CoM implies that $\max \{ r_i, r_j \} < c_j$. The intersection can be characterized as

$$\begin{aligned} D_i \cap D_j = \big \{ {\varvec{x}}\in {\mathbb {R}}^2 ;\ \lambda _i({\varvec{x}}), \lambda _j({\varvec{x}}) \le 1 \big \} = A_{ij}^+ \cup A_{ij}^- , \end{aligned}$$

where

$$\begin{aligned} \begin{aligned} A_{ij}^+ = \big \{ {\varvec{x}}= (x,y) \in D_i \cap D_j ;\ x \ge 0 \big \} , \\ A_{ij}^- = \big \{ {\varvec{x}}= (x,y) \in D_i \cap D_j ;\ x \le 0 \big \} . \end{aligned} \end{aligned}$$

It is clear that $A_{ij}^+ \cap A_{ij}^-$ is the portion of ${\mathcal {H}}_{ij}$ at the intersection of the two discs. Inside $D_i \cap D_j$, consider all points ${\varvec{x}}$ with a fixed value $\lambda _i({\varvec{x}})$, then the set of all possible values of $\lambda _j({\varvec{x}})$ range from 1 down to $(c_j-r_i \lambda _i({\varvec{x}}))/r_j$, where the lower bound occurs along ${\mathcal {H}}_{ij}$ when the gradients of $\lambda _i$ and $\lambda _j$ are multiples. In other words, for each value of $\lambda _i$, the value of $\lambda _j$ therefore ranges between $[{\widehat{\lambda }}_j,1]$ where

$$\begin{aligned} {\widehat{\lambda }}_j(\lambda _i) := \frac{c_j-r_i \lambda _i}{r_j} . \end{aligned}$$

This allows us to define the triangular domain

$$\begin{aligned} T :&= \big \{ (\lambda _i,\lambda _j) \in {\mathbb {R}}^2 ;\ (c_j-r_j)/r_i \le \lambda _i \le 1,\ {\widehat{\lambda }}_j(\lambda _i) \\&\le \lambda _j \le 1 \big \} . \end{aligned}$$

Our objective is now to show that the maps

79 $$\begin{aligned} \begin{array}{rl} \varphi ^\pm : A_{ij}^\pm &{} \longrightarrow T \\ {\varvec{x}}&{} \longmapsto \big ( \lambda _i({\varvec{x}}),\lambda _j({\varvec{x}}) \big ) . \end{array} \end{aligned}$$

are bijective diffeomorphisms. Given a point $(\lambda _i,\lambda _j) \in T$, we will find a point ${\varvec{x}}\in A_{ij}^+$, or $A_{ij}^-$, such that

$$\begin{aligned} \varphi ^+({\varvec{x}}) = (\lambda _i,\lambda _j), \quad \text {or}\ \varphi ^-({\varvec{x}}) = (\lambda _i,\lambda _j) . \end{aligned}$$

For ${\varvec{x}}= (x,y)$, the map is

$$\begin{aligned} \lambda _i^2 =&\Big ( \frac{x}{r_i} \Big )^2 + \Big ( \frac{y}{r_i} \Big )^2 , \\ \lambda _j^2 =&\Big ( \frac{x-c_j}{r_j} \Big )^2 + \Big ( \frac{y}{r_j} \Big )^2 . \end{aligned}$$

If we isolate the *x* coordinate, we find

$$\begin{aligned} r_j^2 \lambda _j^2 - r_i^2 \lambda _i^2= & {} (x-c_j)^2 -x^2 = -2xc_j + c_j^2 \quad \\&\Longrightarrow \quad x = \frac{1}{2c_j} \big ( r_i^2\lambda _i^2 - r_j^2 \lambda _j^2 + c_j^2 \big ) . \end{aligned}$$

Substituting back into the equation for $\lambda _i^2$ and simplifying

$$\begin{aligned} y^2 = r_i^2 \lambda _i^2 - x^2 = r_i^2 \lambda _i^2 - \frac{1}{4 c_j^2} \Big ( r_i^2\lambda _i^2 - r_j^2 \lambda _j^2 + c_j^2 \Big )^2 . \end{aligned}$$

This equation possesses two solutions for *y*, corresponding to either the map $\varphi ^+$ or $\varphi ^-$. Taking $\lambda _i = \lambda _j = 1$, we can quickly verify that there are only two solutions at the intersection of the two circles. From the construction, it is clear that $(x,y) \in A_{ij}^\pm$.

The previous calculation shows that the maps $\varphi ^\pm$ are bijective between $A_{ij}^\pm$ and *T*. To show that they are diffeomorphisms, we compute the differential by first observing that

$$\begin{aligned} \nabla f_k = \nabla \lambda _k^2 = 2 \lambda _k \nabla \lambda _k , \quad k=i,j, \end{aligned}$$

hence

$$\begin{aligned} d \varphi ^\pm ({\varvec{x}}) = \begin{bmatrix} \nabla \lambda _i \\ \nabla \lambda _j \end{bmatrix} = \begin{bmatrix} \dfrac{1}{2\lambda _i} \nabla f_i \\ \dfrac{1}{2\lambda _j} \nabla f_j \end{bmatrix}. \end{aligned}$$

If one recalls the definition (68) of *h*, one can conclude that

$$\begin{aligned} {\text {det}} \big ( d \varphi ^\pm ({\varvec{x}}) \big ) = \frac{h({\varvec{x}})}{4\lambda _i({\varvec{x}}) \lambda _j ({\varvec{x}})} . \end{aligned}$$

In the case of two circles, a simple calculation shows that

$$\begin{aligned} {\text {det}} \big ( d \varphi ^\pm ({\varvec{x}}) \big ) = \frac{y c_j}{\lambda _i({\varvec{x}}) \lambda _j ({\varvec{x}})r_i^2 r_j^2} , \end{aligned}$$

which only vanishes along the co-gradient locus, that is, along part of the boundary of $A_{ij}^\pm$. $\square$

**Fig. 7:**
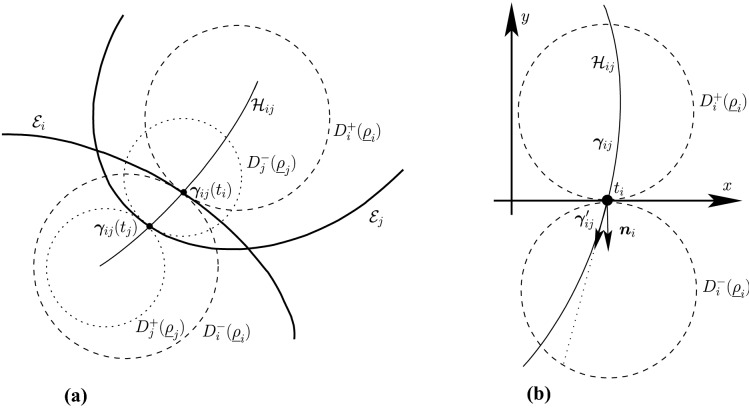
(**a**) Illustration of disks $D_k^\pm ({\underline{\rho }}_k)$ at points $\varvec{\gamma }_{ij}(t_k)$ with $k=i,j$ for a pair of ellipses ${\mathcal {E}}_i$ and ${\mathcal {E}}_j$. (**b**) Transformation of the two disks $D_i^\pm ({\underline{\rho }}_i)$ with $\varvec{\gamma }_{ij}(t_i)$ sent to the origin and the tangent line to ${\mathcal {E}}_i$ at $t_i$ aligned with the horizontal *x*-axis

The proof of the next theorem will show that the condition of near perfect contact implies that the pair of ellipses satisfy two geometric properties. To explain these conditions, we introduce $D_k^+(r)$ the disc of radius *r* tangent to ${\mathcal {E}}_k$ at $\varvec{\gamma }_{ij}(t_k)$ and whose interior is disjoint from $E_k:= \{ {\varvec{x}};\ f_k({\varvec{x}})\le 0 \}$. Similarly, let $D_k^-(r)$ be the disc of radius *r* tangent to ${\mathcal {E}}_k$ whose interior lies inside $E_k$. Intuitively, $D_k^+(r)$ ($D_k^-(r)$) is the disc tangent to ${\mathcal {E}}_k$ placed on the outside (inside) of ${\mathcal {E}}_k$, see Fig. 7a. We will also be using ${\underline{\rho }}_k$ and ${\overline{\rho }}_k$ the minimum and the maximum of the radius of curvature of the ellipse ${\mathcal {E}}_k$, respectively.

Definition 6 implies that

1. the discs $D_i^-({\underline{\rho }}_i)$ and $D_j^-({\underline{\rho }}_j)$ have non-penetrating CoM; and
2. the portion of ${\mathcal {H}}_{ij}$ inside the intersection of the two ellipses is entirely inside the intersection $D_i^-({\underline{\rho }}_i) \cap D_j^-({\underline{\rho }}_j)$.

Both conditions are generalizations of what was observed in the proof of Lemma 11. We now proceed with the proof of Theorem 7.

#### Proof

This will not be a complete proof of Theorem 7 but an attempt at explaining how the condition is related to the two geometrical properties in the previous remark, as well as the maps $\varphi ^\pm$ describing the intersection.

We begin by studying the neighborhood of the point $\varvec{\gamma }_{ij}(t_i) \in {\mathcal {E}}_i$. The tangent to the curve $\varvec{\gamma }_{ij}$ at $\varvec{\gamma }_{ij}(t_i)$ is not necessarily in the same direction as the normal ${\varvec{n}}_i( \varvec{\gamma }_{ij}(t_i))$, but certainly not perpendicular, and we note the defect as

$$\begin{aligned} \eta = {\text {arccos}}\bigg ( \frac{{\varvec{n}}_i( \varvec{\gamma }_{ij}(t_i)) \cdot \varvec{\gamma }_{ij}'(t_i)}{\Vert \varvec{\gamma }_{ij}'(t_i)\Vert } \bigg ) \in \ ]-\pi /2,\pi /2[. \end{aligned}$$

We now establish a condition under which the tangent line to $\varvec{\gamma }_{ij}$ at $\varvec{\gamma }_{ij}(t_i)$ remains inside $D_i^-({\underline{\rho }}_i)\cup D_i^+({\underline{\rho }}_i)$. Consider a translation of $\varvec{\gamma }_{ij}(t_i)$ to the origin, then apply a rotation to send the tangent line to ${\mathcal {E}}_i$ at $t_i$ to the horizontal *x*-axis, as in Fig. 7b. We observe that we can reduce the analysis to showing that a curve crossing the origin at an angle $\eta$ with respect to the vertical axis remains inside the discs tangent to the *x*-axis centered at the origin.

Recall Formula (18) for the smallest radius of curvature ${\underline{\rho }}_i:= b_i^2/a_i$, and the parameterization of the points on the boundary of $D_i^-({\underline{\rho }}_i)$, centered at $(0,-{\underline{\rho }}_i)$ below the horizontal axis:

$$\begin{aligned} \big ({\underline{\rho }}_i\cos (\theta ),{\underline{\rho }}_i\sin (\theta ) -{\underline{\rho }}_i \big ), \quad \forall \theta \in [-\pi ,\pi [. \end{aligned}$$

The smooth curve $\varvec{\gamma }_{ij}$ remains close to its tangent line $s \varvec{\gamma }_{ij}'(t_i)$, $\forall s \in {\mathbb {R}}$, as long as *t* stays close to $t_i$. We now compute the length of the portion of the tangent line inside $D_i^-({\underline{\rho }}_i)$, which by symmetry will be of the same length inside $D_i^+({\underline{\rho }}_i)$. The tangent line forms an angle $\eta$ with the vertical axis. Let ${\mathbf {p}} \ne {\mathbf {0}}$ be the unique point on $D_i^-({\underline{\rho }}_i)$ where the tangent line crosses. We observe that an equilateral triangle is formed between the origin ${\mathbf {0}}$, the center $(0, -{\underline{\rho }}_i)$, and the point ${\mathbf {p}}$ with two sides of length ${\underline{\rho }}_i$ and two angles of measure $\eta$. The length we are looking for is the length of the side of this equilateral triangle opposite to the center $(0, -{\underline{\rho }}_i)$. Based on this geometry, it is easy to verify that the length of the portion of the tangent line inside $D_i^-({\underline{\rho }}_i)$ is

$$\begin{aligned} 2{\underline{\rho }}_i \cos (\eta ) = 2 {\underline{\rho }}_i {\varvec{n}}_i\big ( \varvec{\gamma }_{ij}(t_i)\big ) \cdot \frac{ \varvec{\gamma }_{ij}'(t_i) }{\Vert \varvec{\gamma }_{ij}'(t_i)\Vert }. \end{aligned}$$

The curve $\varvec{\gamma }_{ij}(t)$ will remain close to its tangent line as long as the parameter *t* stays close to $t_i$, with the deviation being proportional to $|t-t_i|^2$ and the curvature of $\varvec{\gamma }_{ij}$. Requiring that the parameterized distance $|t_i - t_j|$ along the curve $\varvec{\gamma }_{ij}$ be small when compared to the length above implies that the curve cannot exit $D_i^+ \cap D_i^-$ and hence the portion of ${\mathcal {H}}_{ij}$ with *t* close to both $t_i$ and $t_j$ crosses ${\mathcal {E}}_i$ only once. The minimum over *i* and *j* in (60) also constrains the co-gradient locus, at least the part inside $D_j^-({\underline{\rho }}_j)\cup D_j^+({\underline{\rho }}_j)$, to contain only a single point from ${\mathcal {E}}_j$. It is also clear that by taking $t_j-t_i$ sufficiently small and negative, then the two discs $D_i^-({\underline{\rho }}_i)$ and $D_j^-({\underline{\rho }}_j)$ will satisfy the non-penetrating CoM condition.

We can now distinguish the following three cases:

- If $t_i=t_j$, then the definition of the co-gradient locus implies that both ellipses have opposing normals and that the two ellipses are in perfect contact.
- Suppose now that both $t_i < t_j$ and (60) are satisfied. The portion of the co-gradient locus between $\varvec{\gamma }_{ij}(t_i)$ and $\varvec{\gamma }_{ij}(t_j)$ must be inside $D_i^+({\underline{\rho }}_i)\cup D_j^+({\underline{\rho }}_j)$, which is outside of both ellipses. There exists a unique ellipse ${\mathcal {E}}_j(r_j)$ which is tangent to ${\mathcal {E}}_i$ at $\varvec{\gamma }_{ij}(t_i)$ and because $\varvec{\gamma }_{ij}(t_i)$ is outside of $E_j$, $r_j$ must be greater than 1. Since both ${\mathcal {E}}_i$ and ${\mathcal {E}}_j(r_j)$ are convex and tangent, $E_i$ is disjoint from ${\mathcal {E}}_j(r_j)$ and the set $E_j$ is strictly bounded by ${\mathcal {E}}_j(r_j)$. These remarks imply that the ellipses are disjoint when $t_i < t_j$.
- Suppose now that both $t_j < t_i$ and (60) hold. The objective is to show that ${\mathcal {E}}_i\cap {\mathcal {E}}_j$ contains exactly two points, which is fundamentally no longer a question only about a neighborhood of $\varvec{\gamma }_{ij}(t)$ and $t \in [t_i,t_j]$ but a global question about a region. The question will be answered by providing a detailed description of the region at the intersection $E_i \cap E_j$. The characterization of the intersection ${\mathcal {E}}_i \cap {\mathcal {E}}_j$ will be a simple corollary of the description of $E_i \cap E_j$. We shall show that there exists a diffeomorphism of a triangle toward each half of the domain $E_i \cap E_j {\setminus } {\mathcal {H}}_{ij}$.

**Fig. 8:**
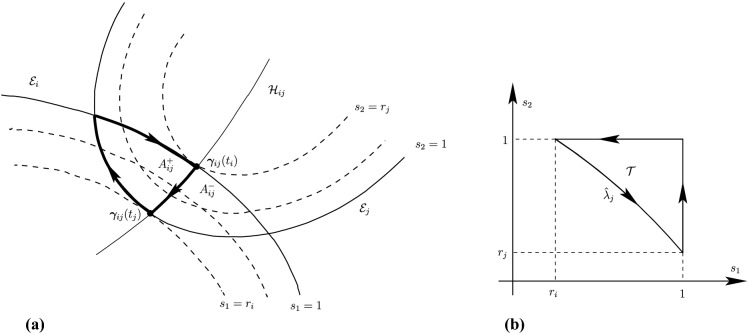
Illustration of the proof of Theorem 7: (**a**) The ellipses $${\mathcal {E}}_i$$ and $${\mathcal {E}}_j$$ are in near-perfect contact with overlap. The region $$ A_{ij}^+$$ is illustrated with bold boundary. (**b**) The region $${\mathcal {T}}$$ is the region mapped by function $$\varvec{\varphi }$$ from region $$ A_{ij}^+$$ and the curve $${\hat{\lambda }}_j$$ is mapped from the curve $$A_{ij}^+ \cap A_{ij}^-$$

We begin by identifying a subdivision of $E_i \cap E_j$. Following the definition of *h* in (66), we can construct

$$\begin{aligned} A_{ij}^+ =&\big \{ {\varvec{x}}\in E_i \cap E_j;\ h({\varvec{x}}) \ge 0 \big \}, \\ A_{ij}^- =&\big \{ {\varvec{x}}\in E_i \cap E_j;\ h({\varvec{x}}) \le 0 \big \}, \end{aligned}$$

and deduce that

$$\begin{aligned} A_{ij}^+ \cup A_{ij}^- = E_i \cap E_j . \end{aligned}$$

Intuitively, the normalized co-gradient function (71) can be used to uniquely define the angle $\eta _{ij}({\varvec{x}})$ between the two normals in a neighborhood of ${\mathcal {H}}_{ij}$ according to

$$\begin{aligned} {\hat{h}}({\varvec{x}}) = {\sin \eta _{ij}({\varvec{x}}) = 0} \quad \Longleftrightarrow \quad { \eta _{ij}({\varvec{x}}) = \pi }, \end{aligned}$$

and the convention that we measure increasing $\eta _{ij}$ when rotating counterclockwise from ${\varvec{n}}_i$ to ${\varvec{n}}_j$. Hence, for all ${\varvec{x}}$ in a neighborhood of ${\mathcal {H}}_{ij} = A_{ij}^+ \cup A_{ij}^-$, there is a well-defined angle $\eta _{ij}({\varvec{x}})$ with values near $\pi$. Since the scalar function *h* is the *z*-component of the cross-product ${\varvec{n}}_i \times {\varvec{n}}_j$, the two regions $A_{ij}^+$ and $A_{ij}^-$ correspond to the regions where $\eta _{ij}({\varvec{x}})$ is, respectively, less than $\pi$ and greater than $\pi$, see Fig. 8a.

As introduced earlier in Sect. 2.3, each point ${\varvec{x}}\in {\mathbb {R}}^2$ belongs to unique scaled ellipses ${\mathcal {E}}_i(\lambda _i({\varvec{x}}))$ and ${\mathcal {E}}_j(\lambda _j({\varvec{x}}))$ where the real-valued functions

$$\begin{aligned} \lambda _k({\varvec{x}}) = \sqrt{f_k({\varvec{x}}) +1}, \quad k=i,j, \end{aligned}$$

are smooth away from the centers ${\varvec{c}}_k$. The gradients of these functions are multiples of the gradient because

$$\begin{aligned} \nabla f_k = \nabla \lambda ^2_k = 2 \lambda _k \nabla \lambda _k . \end{aligned}$$

The values of $\lambda _i$ and $\lambda _j$ along the co-gradient locus belong to the intervals $[r_i, 1]$ and $[r_j,1]$, respectively, where $r_i:= \lambda _i(\varvec{\gamma }_{ij}(t_j))$ and $r_j:= \lambda _j( \varvec{\gamma }_{ij}(t_i))$. More precisely, along the segment $\varvec{\gamma }_{ij}([t_i,t_j])$, the parameter $\lambda _i(\varvec{\gamma }_{ij}(t))$ is increasing and $\lambda _j(\varvec{\gamma }_{ij}(t))$ is decreasing and therefore we can parameterize $\lambda _j$ as a function of $\lambda _i$ along the segment, say ${\widehat{\lambda }}_j(\lambda _i)$. From this, we can define a triangular domain

$$\begin{aligned} T = \big \{ (s_1, s_2) \in [r_i, 1] \times [r_j, 1];\ {\widehat{\lambda }}_j(s_1) \le s_2 \le 1 \big \}, \end{aligned}$$

and two smooth maps

$$\begin{aligned} \varvec{\varphi }^{\pm }: A_{ij}^\pm&\, \longrightarrow T \\ {\varvec{x}}&\, \longmapsto \big (\lambda _i({\varvec{x}}), \lambda _j({\varvec{x}}) \big ). \end{aligned}$$

The determinants of the differentials of these maps are

$$\begin{aligned} {\text {det}} \big ( d \varvec{\varphi }^{\pm } \big )= & {} \left| \begin{array}{c} (\nabla \lambda _i )^T \\ (\nabla \lambda _j)^T \end{array} \right| = \frac{1}{4 \lambda _i({\varvec{x}}) \lambda _j({\varvec{x}})} \left| \begin{array}{c} (\nabla f_i )^T \\ (\nabla f_j)^T \end{array} \right| \\= & {} \frac{h({\varvec{x}})}{4 \lambda _i({\varvec{x}}) \lambda _j({\varvec{x}})}, \end{aligned}$$

hence the maps $\varvec{\varphi }^{\pm }$ are of rank 2 except along ${\mathcal {H}}_{ij}$. This implies that each map is locally bijective, at least in the interior of $A_{ij}^{\pm }$.

The analysis presented so far shows that $\varphi ^\pm$ is a diffeomorphism only in a neighborhood of ${\mathcal {H}}_{ij}$. A complete proof would require a proof that $\lambda _j$ be monotone along the boundary of ${\mathcal {E}}_i$ in $A_{ij}^\pm$, and vice versa for $\lambda _i$. This would demonstrate that $\varphi ^\pm$ is bijective along the boundaries. Given that $D^-_k({\underline{\rho }}_k)$ is tangent to ${\mathcal {E}}_k$, it is intuitively clear that if Lemma 11 holds for both discs, then the maps $\varphi ^\pm$ in the case of ellipses should also characterize the intersection $E_i \cap E_j$. $\square$

The proof of Theorem 7 given above also provides the following explicit description of the intersection.

#### Corollary 12

Consider two ellipses ${\mathcal {E}}_i$, ${\mathcal {E}}_j \subset {\mathbb {R}}^2$ in near-perfect contact. If $E_i$ and $E_j$ are the closed regions bounded by the respective ellipses, then the intersection is the union of two closed domains

$$\begin{aligned} E_i \cap E_j = A_{ij}^+ \cup A_{ij}^- , \end{aligned}$$

whose intersection is $A_{ij}^+ \cap A_{ij}^- = {\mathcal {H}}_{ij} \cap (E_i \cap E_j)$. Both $A_{ij}^+$ and $A_{ij}^-$ are diffeomorphic to a triangle.

The proof of existence and uniqueness of the MDP for pairs of ellipses in near-perfect contact, namely Theorem 8, requires both Theorem 7 and Corollary 12. Although the result is intuitively convincing, the proof is long and will not be included in this review because the formulation of the MDP and the structure of the theory is, in the authors opinion, more important than the details of the individual proofs.

### Extension to ellipsoids

The objective of this paper is to review and compare algorithms for the rapid and accurate estimation of separation/penetration distance for pairs of ellipses and ellipsoids in the quasi-static regime. The main mathematical results are Lemma 1, Theorem 6, and Theorem 7, which were all stated and demonstrated in 2-D, so it is natural to enquire as to what can be said in ${\mathbb {R}}^3$. In this section, we will briefly suggest how these three results could be extended to 3-D. Before proceeding, we remark that Definitions 4 and 5 have obvious extensions, that Lemmas 3 and 4, as well as Theorem 5, have identical statements and proofs in 3-D, and that in practice no change is required to the algorithms in Sect. 5.

The most difficult result to extend to 3-D is Theorem 6, and it should be studied with the techniques of algebraic geometry [20, 23]. Lemma 1 was only a preliminary result for the proof of Theorem 6 and provided tools to circumvent real projective geometry; hence, we will discuss only Theorem 6. As far as Theorem 7 is concerned, the extension to 3-D is straightforward assuming that ${\mathcal {H}}_{ij}$ has been characterized. In fact, our initial proof of Theorem 7 was carried out in 3-D and the key map $\varvec{\varphi }$ is slightly easier to study because the intersection $E_i \cap E_j$ is path-connected (but not simply connected). In ${\mathbb {R}}^3$, the additional parameter in the intersection $E_i \cap E_j$ parameterizes a circle $S^1$ whose tangent is given by ${\varvec{n}}_i \times {\varvec{n}}_j = {\mathbf {h}}$.

The statement in 3-D of Theorem 6 concerns the co-gradient locus ${\mathcal {H}}_{ij}$, which is now the set of common roots of each of the three components of the cross-product (56), each of which can be written roughly in a quadratic form similar to (68). Each component defines a two-dimensional subvariety that intersects the sphere at infinity in real projective space ${\mathbb {R}}P^3$ along a curve. We conjecture that, as we did in 2-D, we can characterize the common zeros in ${\mathbb {R}}^3$ by identifying the isolated intersection points of the three 1-D curves at infinity. Each ellipse will define three planes corresponding to each pair of its orthogonal axis, where the normals take on known values. These six curves on the sphere at infinity will provide a triangular subdivision of the sphere and the existence of a root to (56) in each triangle will be determined by examining the signs of the components of ${\varvec{h}}$ along the edges and nodes of the triangle.

### Relationship to time-dependent contact detection

For pairs of rapidly moving and/or rotating ellipses/ellipsoids, estimating the contact point and the penetration distance requires anticipating future positions. More specifically, the future contact point might be very different from the MDP at a given time when the relative velocity of the two particles is large and their surfaces are close. In this section, we show that the constraint of the co-gradient locus ${\mathcal {H}}_{ij}$ appearing in the MPP can be interpreted as enforcing displacements of the ellipses along a specific trajectory. In other words, we can interpret ${\mathcal {H}}_{ij}$ as a specific particle trajectory bringing the two ellipses in contact. As we mentioned in the Introduction, the purpose of this review is not to study time-dependent collision detection for which there exist particular techniques, such as the *continuous contact detection method* of Wang et al. [6], but to highlight similarities that might allow one to formulate analogous results to those in Theorems 8 and 9 for time-dependent problems.

Consider two ellipses ${\mathcal {E}}_i$, ${\mathcal {E}}_j$ that are disjoint but in near-perfect contact. Consider the parameterization $\varvec{\gamma }_{ij}$ of the curve ${\mathcal {H}}_{ij}$ going from ${\varvec{c}}_i = \varvec{\gamma }_{ij}(0)$ to ${\varvec{c}}_j = \varvec{\gamma }_{ij}(1)$, and fix $t \in \ ]t_i,t_j[$ corresponding to $\varvec{\gamma }_{ij}(t)$ in the exterior of both ellipses. Along the directed line $\overline{{\varvec{c}}_j \varvec{\gamma }_{ij}(t)}$, there is one unique intersection point ${\varvec{p}}_j(t) \in {\mathcal {E}}_j,$ which depends analytically on *t*, and the translation is defined by

$$\begin{aligned} T_t : \; {\mathbb {R}}^2&\longrightarrow {\mathbb {R}}^2 \\ \; {\varvec{x}}&\longmapsto {\varvec{x}}+ \big ( \varvec{\gamma }_{ij}(t) - {\varvec{p}}_j(t) \big ). \end{aligned}$$

Remark that $\varvec{\gamma }_{ij}(t)-{\varvec{c}}_j$ and $\varvec{\gamma }_{ij}(t)-{\varvec{p}}_j(t)$ are colinear; hence, the displaced ellipse $T_t {\mathcal {E}}_j$ has the same normal along the line $T_t {\varvec{c}}_j$ to $T_t {\varvec{p}}_j(t)$ as it had along the line connecting ${\varvec{c}}_j$ to $\varvec{\gamma }_{ij}(t)$. This implies that $T_t$ will map ${\varvec{p}}_j(t_i)$ to $\varvec{\gamma }_{j}(t_i)$ and that at $\varvec{\gamma }_{ij}(t_i)$, the normal to $T_t {\mathcal {E}}_j$ will be the same as ${\varvec{n}}_j$ for ${\mathcal {E}}_j$. The definition of ${\mathcal {H}}_{ij}$ then implies that the image of $T_{t_i} {\mathcal {E}}_j$ will be in perfect contact with ${\mathcal {E}}_i$.

We have therefore shown that as *t* goes from $t_j$ to $t_i$, the transformation $T_t$ brings ${\mathcal {E}}_j$ in contact with ${\mathcal {E}}_i$ at $\varvec{\gamma }_{ij}(t)$. This transformation does not correspond to a free fall trajectory for ${\mathcal {E}}_j$, because on the one hand, each translation $T_t$ preserves the orientation and therefore the physical displacement could not have applied torque. Yet, the displacement of the center of mass $T_t {\varvec{c}}_j$ is determined (indirectly) by the points on a hyperbola in any rotated frame of reference, and therefore does not follow a parabola. Hence, $T_t$ does not describe a physical displacement. Nevertheless, this construction provides an additional interpretation of ${\mathcal {H}}_{ij}$ as a canonical trajectory associated with each pair of ellipses.

Finally, we would like to highlight the research of Wang and his collaborators done for rapidly moving particles. That research did not attempt to measure separation/penetration distance but only to identify whether the ellipsoids are disjoint or overlapping (or in perfect contact). In 2001, Wang, Wang and Kim [52] found an algebraic condition for the separation of two ellipsoids. They demonstrated that a certain characteristic polynomial $p_{ij}$ of degree 4 had two positive real roots precisely when the ellipsoids were disjoint. In [51], the authors showed that the earlier algebraic condition could be used to identify a plan separating the two ellipsoids. The use of a separating plane was shown to be criteria that could be reused for several consecutive timesteps, without additional computation, if the linear and angular velocities of the particles were not too large, and hence lead to significant savings. The work in [26] proposed to apply an improved criterion [3] for the determination of the sign of the roots of the characteristic polynomial $p_{ij}$ from the original work [52]. In the spirit of Descartes’ rule of signs, this extension involved using determinants of submatrices of the *i*th Sylvester–Habicht matrix, which is a family of reduced resultant matrices. This technique produced increased computational efficiency for the contact detection problem, but still without a computation of the distance between the ellipsoids.

## Initializing the positions of two ellipses for contact detection

Some algorithms to be presented in Sect. 5 share two common features. The first is that the problem can be simplified by introducing normalized coordinates where one of the ellipses has become a circle. The second common feature is that they may require an initial guess of the contact point, and obviously, the efficiency of an iterative algorithm will be highly dependent on the quality of that initial guess. In this section, we review two mapping steps; see Sects. 4.1 and 4.2. The mapping will simplify the later presentation of the algorithms, and one of our proposed techniques for guessing the contact point (see Sect. 4.3) is done in one of the normalized coordinate systems we introduce below. We remark that the estimation of the contact point is rarely addressed in the literature and, to the best of our knowledge, the *focal point* estimate was first presented by the authors in [29].

### Mapping of $({\mathcal {E}}_i,{\mathcal {E}}_j)$ into a unit circle ${\widehat{{\mathcal {C}}}}_i$ centered at origin and an ellipse ${\widehat{{\mathcal {E}}}}_j$

The first mapping was suggested by Ting et al. in [46]. Let ${\mathcal {E}}_i$ and ${\mathcal {E}}_j$ be two arbitrary ellipses defined by

80 $$\begin{aligned} f_i({\varvec{x}})&= ({\varvec{x}}-{\varvec{c}}_i)^T {\mathcal {Q}}_i ({\varvec{x}}-{\varvec{c}}_i) - 1 \nonumber \\&= ({\varvec{x}}-{\varvec{c}}_i)^T {\mathcal {R}}_i {\mathcal {D}}_i {\mathcal {R}}_i^T ({\varvec{x}}- {\varvec{c}}_i) - 1 = 0, \end{aligned}$$

81 $$\begin{aligned} f_j({\varvec{x}})&= ({\varvec{x}}-{\varvec{c}}_j)^T {\mathcal {Q}}_j ({\varvec{x}}-{\varvec{c}}_j) - 1 \nonumber \\&= ({\varvec{x}}-{\varvec{c}}_j)^T {\mathcal {R}}_j {\mathcal {D}}_j {\mathcal {R}}_j^T ({\varvec{x}}- {\varvec{c}}_j) - 1 = 0, \end{aligned}$$

where the diagonal matrices ${\mathcal {D}}_i$ and ${\mathcal {D}}_j$ and the rotation matrices ${\mathcal {R}}_i$ and ${\mathcal {R}}_j$ are as defined in (3) and (2), respectively.

**Fig. 9:**
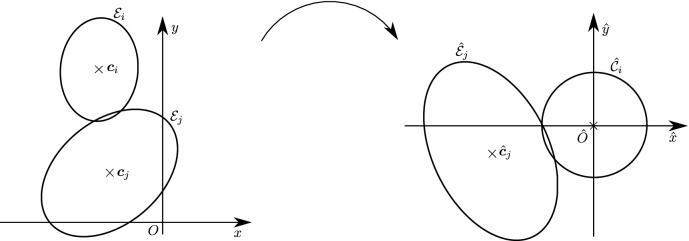
Mapping of $({\mathcal {E}}_i,{\mathcal {E}}_j)$ into a unit circle $\hat{{\mathcal {C}}}_i$ centered at the origin and an ellipse $\hat{{\mathcal {E}}}_j$

The mapping consists in transforming one of the two ellipses, say ${\mathcal {E}}_i$, into the unit circle $\hat{{\mathcal {C}}}_i$ centered at the origin and the other ellipse ${\mathcal {E}}_j$ into the ellipse ${\hat{{\mathcal {E}}}}_j$; see Fig. 9. The mapping that transforms $\hat{{\mathcal {C}}}_i$ into ${\mathcal {E}}_i$ consists of the transformation ${\mathcal {D}}_i^{-1/2}$, followed by the rotation ${\mathcal {R}}_i$ of angle $\theta _i$ and the translation ${\varvec{c}}_{i}$. We thus have

82 $$\begin{aligned} {\varvec{x}}= {\mathcal {R}}_i {\mathcal {D}}_i^{-1/2} {\hat{{\varvec{x}}}} + {\varvec{c}}_{i}. \end{aligned}$$

Indeed, it is straightforward to check that the equation of the circle $\hat{{\mathcal {C}}}_i$ in the coordinate system $({\hat{O}},{\hat{x}},{\hat{y}})$, using (82) in (80), thus reads

83 $$\begin{aligned} {\hat{f}}_i({\hat{{\varvec{x}}}}) = {\hat{{\varvec{x}}}}^T {\hat{{\varvec{x}}}} - 1 = 0. \end{aligned}$$

The equation of the new ellipse ${\hat{{\mathcal {E}}}}_j$, following the mapping of ellipse ${\mathcal {E}}_j$, is obtained by substituting (82) in (81). We find that ${\hat{{\mathcal {E}}}}_j$ in the $({\hat{O}},{\hat{x}},{\hat{y}})$ coordinates are the roots of

$$\begin{aligned} {\hat{f}}_j({\hat{{\varvec{x}}}}) = ( {\hat{{\varvec{x}}}} - {\hat{{\varvec{c}}}}_{j} )^T {\mathcal {D}}_i^{-1/2} {\mathcal {R}}_i^T {{\mathcal {Q}}}_j {\mathcal {R}}_i {\mathcal {D}}_i^{-1/2} ( {\hat{{\varvec{x}}}} - {\hat{{\varvec{c}}}}_{j} ) - 1 = 0, \end{aligned}$$

where we have introduced ${\hat{{\varvec{c}}}}_{j}$ satisfying

84 $$\begin{aligned} {\mathcal {R}}_i {\mathcal {D}}_i^{-1/2} {\hat{{\varvec{c}}}}_{j} = {\varvec{c}}_{j}-{\varvec{c}}_{i}. \end{aligned}$$

The equation for ${\hat{f}}_j$ reduces to

85 $$\begin{aligned} {\hat{f}}_j({\hat{{\varvec{x}}}}) = ( {\hat{{\varvec{x}}}} - {\hat{{\varvec{c}}}}_{j} )^T {\hat{{\mathcal {Q}}}}_j ( {\hat{{\varvec{x}}}} - {\hat{{\varvec{c}}}}_{j} ) - 1 = 0, \end{aligned}$$

where

$$\begin{aligned} \begin{aligned} {\hat{{\mathcal {Q}}}}_j \equiv {\mathcal {D}}_i^{-1/2} {\mathcal {R}}_i^T {\mathcal {Q}}_j {\mathcal {R}}_i {\mathcal {D}}_i^{-1/2} = {\mathcal {D}}_i^{-1/2} {\mathcal {R}}_i^T {\mathcal {R}}_j {\mathcal {D}}_j {\mathcal {R}}_j^T {\mathcal {R}}_i {\mathcal {D}}_i^{-1/2}. \end{aligned} \end{aligned}$$

For the sake of completeness, we remark that the coefficients of ${\hat{{\mathcal {Q}}}}_j$, i.e., 

$$\begin{aligned} {\hat{{\mathcal {Q}}}}_j = \begin{bmatrix} {\hat{A}}_j &{} {\hat{C}}_j \\ {\hat{C}}_j &{} {\hat{B}}_j \end{bmatrix} \end{aligned}$$

can be found explicitly as

$$\begin{aligned} \begin{aligned}&{\hat{A}}_j = a_i^2 \left( \frac{\cos ^2 (\theta _j-\theta _i) }{a_j^2} + \frac{\sin ^2 (\theta _j-\theta _i) }{b_j^2} \right) , \\&{\hat{B}}_j = b_i^2 \left( \frac{\sin ^2 (\theta _j-\theta _i) }{a_j^2} + \frac{\cos ^2 (\theta _j-\theta _i) }{b_j^2} \right) , \\&{\hat{C}}_j = a_i b_i \left( \frac{1}{a_j^2} - \frac{1}{b_j^2} \right) \cos (\theta _j-\theta _i) \sin (\theta _j-\theta _i), \end{aligned} \end{aligned}$$

and the parameters $\{{\hat{a}}_j, {\hat{b}}_j, {\hat{\theta }}_j\}$ associated with ellipse ${\hat{{\mathcal {E}}}}_j$, so that ${\hat{{\mathcal {Q}}}}_j= {\hat{{\mathcal {R}}}}_j {\hat{{\mathcal {D}}}}_j {\hat{{\mathcal {R}}}}_j^T$, can be recovered by identification from Formula (10).

The following lemma explains that the minimization problem in the new coordinates is related to a minimization in the original coordinates.

#### Lemma 13

In the coordinate system $({\hat{O}},{\hat{x}},{\hat{y}})$, the Euclidean distance is the same as the distance with respect to the ${\mathcal {E}}_i$-norm in the original coordinates (*O*, *x*, *y*).

#### Proof

For every ${\varvec{x}}$, its image ${\hat{{\varvec{x}}}}$ is computed as

$$\begin{aligned} {\mathcal {D}}^{1/2} {\mathcal {R}}_i^T({\varvec{x}}- {\varvec{c}}_i) = {\hat{{\varvec{x}}}}. \end{aligned}$$

We then have, for arbitrary ${\hat{{\varvec{x}}}}$ and ${\hat{{\varvec{y}}}}$,

$$\begin{aligned} \begin{aligned} \big \Vert {\hat{{\varvec{x}}}} - {\hat{{\varvec{y}}}} \big \Vert ^2&= \big ( {\hat{{\varvec{x}}}} - {\hat{{\varvec{y}}}} \big )^T \big ( {\hat{{\varvec{x}}}} - {\hat{{\varvec{y}}}} \big ) \\&= \Big ( {\mathcal {D}}_i^{1/2} {\mathcal {R}}_i^T({\varvec{x}}- {\varvec{c}}_i) - {\mathcal {D}}_i^{1/2} {\mathcal {R}}_i^T({\varvec{y}}- {\varvec{c}}_i) \Big )^T\\&\quad \times \, \Big ( {\mathcal {D}}_i^{1/2} {\mathcal {R}}_i^T({\varvec{x}}- {\varvec{c}}_i) - {\mathcal {D}}_i^{1/2} {\mathcal {R}}_i^T({\varvec{y}}- {\varvec{c}}_i) \Big ) \\&= \Big ( \big ( {\varvec{x}}- {\varvec{c}}_i\big ) - \big ( {\varvec{y}}- {\varvec{c}}_i\big ) \Big )^T \Big ( {\mathcal {D}}_i^{1/2} {\mathcal {R}}_i^T \Big )^T {\mathcal {D}}_i^{1/2} {\mathcal {R}}_i^T \\&\quad \times \, \Big ( \big ( {\varvec{x}}- {\varvec{c}}_i\big ) - \big ( {\varvec{y}}- {\varvec{c}}_i\big ) \Big ) \\&= \big ( {\varvec{x}}- {\varvec{y}}\big )^T {\mathcal {R}}_i {\mathcal {D}}_i{\mathcal {R}}_i^T \big ( {\varvec{x}}- {\varvec{y}}\big ) \\&= \big \Vert {\varvec{x}}- {\varvec{y}}\big \Vert _{{\mathcal {Q}}_i}^2. \end{aligned} \end{aligned}$$

$$\square $$

### Mapping of $({\mathcal {E}}_i,{\mathcal {E}}_j)$ into a unit circle $\bar{{\mathcal {C}}}_i$ and an ellipse ${\bar{{\mathcal {E}}}}_j$ centered at origin

**Fig. 10:**
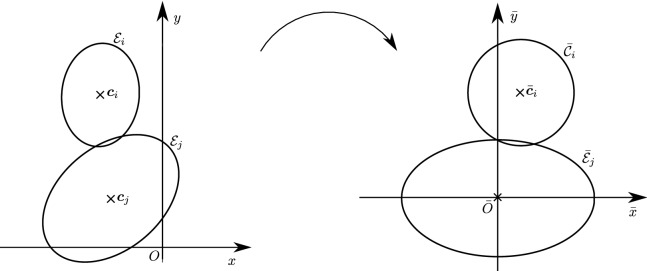
Mapping of $({\mathcal {E}}_i,{\mathcal {E}}_j)$ into a unit circle $\bar{{\mathcal {C}}}_i$ and an ellipse ${\bar{{\mathcal {E}}}}_j$ centered at the origin

Džiugys and Peters [12] suggested another mapping such that the ellipse ${\hat{{\mathcal {E}}}}_j$ introduced above is now positioned in its local reference system denoted here as $({\bar{O}},{\bar{x}},{\bar{y}})$. We will describe this new mapping as the previous mapping followed by a rotation and a translation sending the previous ellipse ${\hat{{\mathcal {E}}}}_j$ to the origin with axes aligned with $({\bar{O}},{\bar{x}},{\bar{y}})$. The circle $\hat{{\mathcal {C}}}_i$, previously at the origin under the mapping of Sect. 4.1, is now shifted around the ellipse centered at the origin, see Fig. 10.

The mapping amounts to considering the transformation that maps ${\bar{{\varvec{x}}}}$ into ${\hat{{\varvec{x}}}}$ in ${\mathbb {R}}^2$ by the rotation ${\hat{{\mathcal {R}}}}_j$ and translation ${\hat{{\varvec{c}}}}_j$:

86 $$\begin{aligned} {\hat{{\varvec{x}}}} = {\hat{{\mathcal {R}}}}_j {\bar{{\varvec{x}}}} + {\hat{{\varvec{c}}}}_j, \end{aligned}$$

so that circle ${\hat{{\mathcal {C}}}}_i$, located at the origin, is now mapped into circle ${\bar{{\mathcal {C}}}}_i$ with center ${\bar{{\varvec{c}}}}_i$ as

$$\begin{aligned} {\bar{{\varvec{c}}}}_i = - {\hat{{\mathcal {R}}}}_j^T {\hat{{\varvec{c}}}}_j. \end{aligned}$$

The equations of the two ellipses in the new coordinate system $({\bar{O}},{\bar{x}}, {\bar{y}})$ are then given by

87 $$\begin{aligned} \begin{aligned}&{\bar{f}}_i ({\bar{{\varvec{x}}}}) = ({\bar{{\varvec{x}}}}-{\bar{{\varvec{c}}}}_i)^T ({\bar{{\varvec{x}}}}-{\bar{{\varvec{c}}}}_i) - 1 = 0, \\&{\bar{f}}_j ({\bar{{\varvec{x}}}}) = {\bar{{\varvec{x}}}}^T {\hat{{\mathcal {D}}}}_j {\bar{{\varvec{x}}}} - 1 = 0. \end{aligned} \end{aligned}$$

In other words, the original ellipse ${\mathcal {E}}_j$ is now transformed into the ellipse $\bar{{\mathcal {E}}}_j$ centered at the origin and the ellipse ${\mathcal {E}}_i$ is mapped into the unit circle $\bar{{\mathcal {C}}}_i$ centered at ${\bar{{\varvec{c}}}}_i$ using the global transformation obtained by combining (82) and (86)

88 $$\begin{aligned} {\varvec{x}}= & {} {\mathcal {R}}_i {\mathcal {D}}_i^{-1/2} ({\hat{{\mathcal {R}}}}_j {\bar{{\varvec{x}}}} + {\hat{{\varvec{c}}}}_j) + {\varvec{c}}_i = {\mathcal {R}}_i {\mathcal {D}}_i^{-1/2} {\hat{{\mathcal {R}}}}_j {\bar{{\varvec{x}}}} \nonumber \\&\quad +\, \big ( {\mathcal {R}}_i {\mathcal {D}}_i^{-1/2} {\hat{{\varvec{c}}}}_j + {\varvec{c}}_i \big ) = {\mathcal {R}}_i {\mathcal {D}}_i^{-1/2} {\hat{{\mathcal {R}}}}_j {\bar{{\varvec{x}}}} + {\varvec{c}}_j, \end{aligned}$$

where we have used (84) to obtain the last expression.

#### Remark 6

The transformation of an arbitrary pair of ellipses into an ellipse in its local coordinate system and a circle can be useful to simplify the mathematical analysis of the pair of ellipses. As an example, we can easily show that the co-gradient locus in the given configuration (87) is a hyperbola. Recalling the co-gradient function (68), the equation of the co-gradient locus reads in this case

$$\begin{aligned} h({\varvec{x}}) = 4 ({\varvec{x}}-{\varvec{c}}_i)^T {\mathcal {Q}}_i A {\mathcal {Q}}_j ({\varvec{x}}-{\varvec{c}}_j) = 0, \end{aligned}$$

where

$$\begin{aligned} A= & {} \begin{bmatrix} 0 &{} 1 \\ -1 &{} 0 \end{bmatrix}, \qquad {\mathcal {Q}}_i = \begin{bmatrix} 1 &{} 0 \\ 0 &{} 1 \end{bmatrix}, \qquad {\mathcal {Q}}_j = \begin{bmatrix} 1/a_j^2 &{} 0 \\ 0 &{} 1/b_j^2 \end{bmatrix}, \qquad \\ {\varvec{c}}_i= & {} \begin{bmatrix} c_x \\ c_y \end{bmatrix}, \qquad {\varvec{c}}_j = {\varvec{0}}, \end{aligned}$$

so that

$$\begin{aligned} {\mathcal {Q}}_i A {\mathcal {Q}}_j = \begin{bmatrix} 0 &{} 1/a_j^2 \\ -1/b_j^2 &{} 0 \end{bmatrix}. \end{aligned}$$

Developing the above equation leads to

89 $$\begin{aligned} (b_j^2-a_j^2) xy - b_j^2 c_y x + a_j^2 c_x y = 0. \end{aligned}$$

This is actually the equation of a hyperbola in the case that $a_i \ne b_i$. Indeed, using classical formulas, the center of the hyperbola is given by

$$\begin{aligned} \begin{aligned}&x_h = \frac{a_j^2}{b_j^2 - a_j^2} c_x, \\&y_h = \frac{b_j^2}{a_j^2 - b_j^2} c_y, \end{aligned} \end{aligned}$$

and, using the change of variables $\xi = x - x_h$ and $\eta = y - y_h$, the equation can be recast as

$$\begin{aligned} \xi \eta = \frac{a_j^2 b_j^2 }{(a_j^2 - b_j^2)^2} c_x c_y, \end{aligned}$$

which is the equation of an hyperbola. In the case that $a_j = b_j$, the locus reduces to a straight line passing through the origin (i.e., the center of ellipse ${\mathcal {E}}_j$, which is a circle here) and the center of circle ${\mathcal {C}}_i$.

**Fig. 11:**
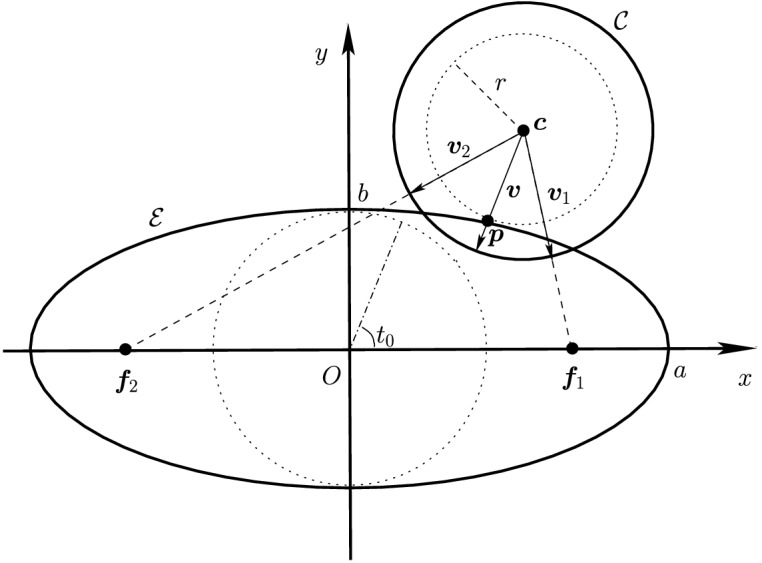
Location of the guess point ${\varvec{p}}={\varvec{c}}+r{\varvec{v}}$ on ellipse ${{\mathcal {E}}}$. The vectors ${\varvec{v}}_1$ and ${\varvec{v}}_2$ are defined with respect to the focal points ${\varvec{f}}_1$ and ${\varvec{f}}_2$. The angle $t_0$ corresponds to the coordinate of the guess point in parametric form, i.e., ${\varvec{p}}=(a\cos t_0,b\sin t_0)$

In the next section, we shall see that the mapping allows one to define accurate initial guess points for the solution of the minimum potential algorithm (GPA).

### Initialization of contact point algorithms

The purpose of this brief section is to discuss possible initial guesses for contact points, taking for granted that the various algorithms will often rely on iterative methods to produce the final estimate of the contact point. In practice, the accuracy and efficiency of iterative methods are closely tied to the quality of the initial guess. In fact, after each timestep of the DEM it is common to use the estimate of the contact point at the previous timestep as an initialization at the current timestep, and so the initial guess may only be required when two ellipsoids are first identified as a pair with a potential contact during the broad phase search. Nevertheless, this question of fundamental importance, that is rarely addressed, should at least be briefly discussed in a thorough review.

Let us consider an ellipse ${\mathcal {E}}$ in its local coordinate system and a unit circle ${\mathcal {C}}$ in near-perfect contact. (We dropped indices *i* and *j* here for the sake of simplification in the notation.) The goal here is to identify a reasonable approximation ${\varvec{p}}$ of the contact point ${\varvec{x}}\in {\mathcal {E}}$. Using the focal points of ${\mathcal {E}}$, which are located at

$$\begin{aligned} {\varvec{f}}_1 = \Big (\sqrt{a^2 - b^2},0 \Big ), \qquad {\varvec{f}}_2 = \Big (-\sqrt{a^2 - b^2},0 \Big ), \end{aligned}$$

one can introduce the two unit vectors ${\varvec{v}}_1$ and ${\varvec{v}}_2$,

$$\begin{aligned}&{\varvec{v}}_1 = ( {\varvec{f}}_1 - {\varvec{c}})/\Vert {\varvec{f}}_1 - {\varvec{c}}\Vert , \\&{\varvec{v}}_2 = ( {\varvec{f}}_2 - {\varvec{c}})/\Vert {\varvec{f}}_2 - {\varvec{c}}\Vert . \end{aligned}$$

We then consider the unit vector ${\varvec{v}}$

90 $$\begin{aligned} {\varvec{v}}=\frac{{\varvec{v}}_1+{\varvec{v}}_2}{\Vert {\varvec{v}}_1+{\varvec{v}}_2\Vert }. \end{aligned}$$

The guess point ${\varvec{p}}$ is then defined as the intersection of ellipse ${\mathcal {E}}$ and the line supported by ${\varvec{v}}$ passing through the center ${\varvec{c}}$ of ${\mathcal {C}}$; see Fig. 11. Parameterizing the line as the set of points ${\varvec{c}}+r {\varvec{v}}$, $r \in {\mathbb {R}}$, the point ${\varvec{p}}$ on ellipse ${\mathcal {E}}$ satisfies

$$\begin{aligned} ({\varvec{c}}+ r {\varvec{v}})^T {\mathcal {D}}({\varvec{c}}+ r {\varvec{v}}) = 0, \end{aligned}$$

which is a quadratic equation in *r*,

$$\begin{aligned} ({\varvec{v}}^T {\mathcal {D}}{\varvec{v}}) r^2 + 2 ({\varvec{v}}^T {\mathcal {D}}{\varvec{c}}) r + ({\varvec{c}}^T {\mathcal {D}}{\varvec{c}}) = 0. \end{aligned}$$

The point ${\varvec{p}}$ that we look for is the closest to ${\varvec{c}}$. Therefore, we choose the smallest of the two roots of the quadratic equation. Introducing the positive quantities $\alpha = ({\varvec{v}}^T {\mathcal {D}}{\varvec{v}})$, $\beta = - ({\varvec{v}}^T {\mathcal {D}}{\varvec{c}})$, and $\gamma = ({\varvec{c}}^T {\mathcal {D}}{\varvec{c}})$, we thus obtain

91 $$\begin{aligned} r = \frac{\beta - \sqrt{\beta ^2 - \alpha \gamma }}{\alpha }, \end{aligned}$$

so that

$$\begin{aligned} {\varvec{p}}= {\varvec{c}}+ r {\varvec{v}}. \end{aligned}$$

Other weighted averages of ${\varvec{v}}_1$ and ${\varvec{v}}_2$ could be considered, but the one in (90) seems to work best for the majority of configurations. In fact, the normal along an ellipse at ${\varvec{p}}$ bisects the angle $\angle {\varvec{f}}_1 {\varvec{p}}{\varvec{f}}_2$ and the simple average (90) is meant to mimic this observation, although in our algorithm ${\varvec{p}}$ is approximated by ${\varvec{c}}$. The optimal weighted average would actually be the one such that the normal vector to ${\mathcal {E}}$ at ${\varvec{p}}$ share the same direction as ${\varvec{v}}$ since ${\varvec{v}}$ would then be the normal unit vector to the circle centered at ${\varvec{c}}$ and of radius *r*, i.e., tangent to ${\mathcal {E}}$. In that case, the point ${\varvec{p}}$ would actually be the solution ${\varvec{x}}$ to the minimum potential pair approach (see Lemma 4). Note that in the case of two arbitrary ellipses, one could use the mapping of Sect. 4.2, find the guess point ${{\varvec{p}}}$ using the algorithm above, and map back ${{\varvec{p}}}$ to the current configuration using transformation (88).

**Fig. 12:**
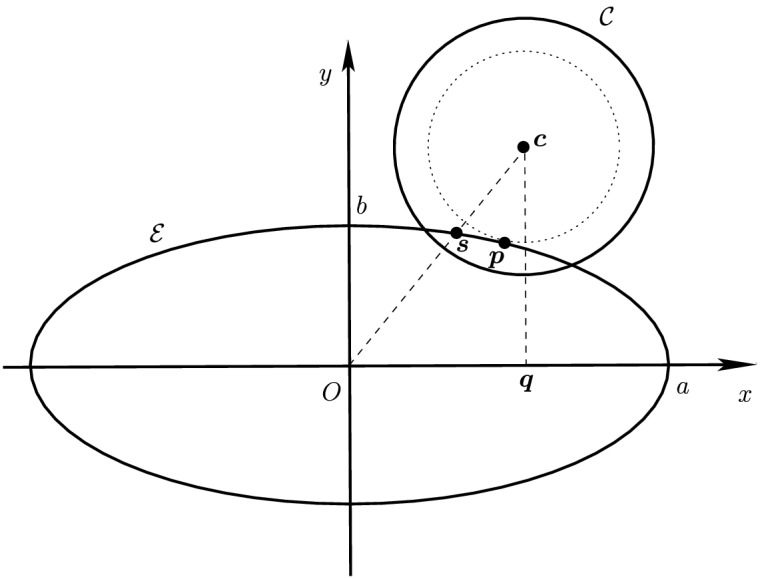
The initial guess point ${\varvec{p}}=(p_x,p_y)$ suggested by Džiugys and Peters [12] can be chosen as any arbitrary point satisfying the conditions (92)

In the same configuration of ${\mathcal {E}}$ and ${\mathcal {C}}$ as before, Džiugys and Peters [12] also proposed conditions to find an initial guess point ${\varvec{p}}$; see Fig. 12. Let ${\varvec{c}}=(c_x,c_y)$ be the center of ${\mathcal {C}}$ and let ${\varvec{q}}$ be defined as $(c_x,0)$. The initial point ${\varvec{p}}$ should be located on the ellipse ${\mathcal {E}}$ inside the triangle ${{\varvec{c}}} {O} {{\varvec{q}}}$. In other words, the coordinates $x_p$ and $y_p$ of point ${\varvec{p}}$ should satisfy the following conditions

92 $$\begin{aligned} \left\{ \begin{aligned}&\text {sign}(x_p)=\text {sign}(c_x),\\&\text {sign}(y_p)=\text {sign}(c_y),\\&|x_p| \le \min (a,|c_x|),\\&|x_p| \ge |s_x|,\\&|y_p| \le |s_y|, \end{aligned} \right. \end{aligned}$$

where ${\varvec{s}}=(s_x,s_y)$ is the intersection point between ellipse ${\mathcal {E}}$ and segment $O{\varvec{c}}$, i.e.,

$$\begin{aligned} s_x = \frac{c_x}{\sqrt{c_x^2/{a}^2+c_y^2/{b}^2}}, \qquad s_y = \frac{c_y}{\sqrt{c_x^2/{a}^2+c_y^2/{b}^2}}. \end{aligned}$$

**Fig. 13:**
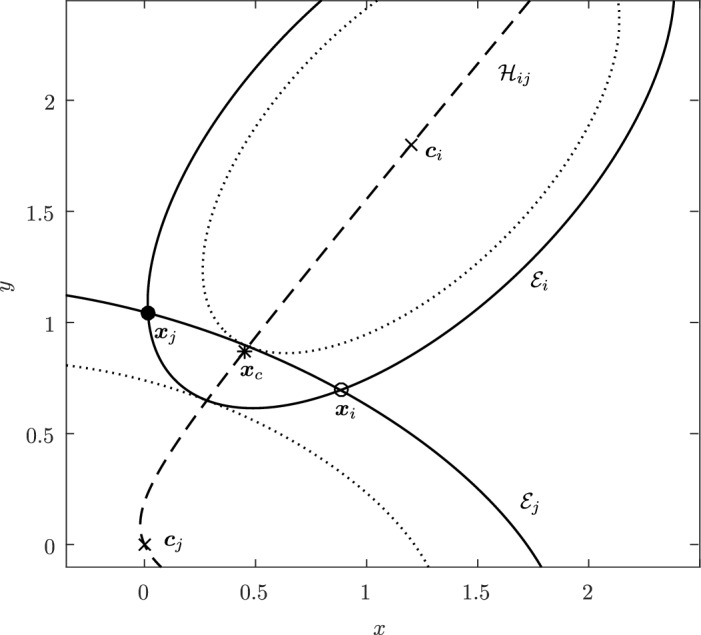
The points ${\varvec{x}}_i$ and ${\varvec{x}}_j$ are the intersection points of ellipses ${\mathcal {E}}_i$ and ${\mathcal {E}}_j$, i.e., ${\mathcal {I}}_{ij}=\{{\varvec{x}}_i,{\varvec{x}}_j\}$ from Definition 2. We observe that the contact point ${\varvec{x}}_c$, obtained here by the Intersection Algorithm (IA), is in this case close to the co-gradient locus ${\mathcal {H}}_{ij}$

## Contact detection algorithms

The main goal of this section is to review the main contact detection algorithms for pairs of ellipses that have been proposed in the literature and, more specifically, recast the methods as minimization problems such as those satisfied by the MDP and MPP introduced in Sect. 3. Many of the algorithms to be discussed were not explicitly defined as minimization problems (with or without explicit constraints) and were often categorized differently by the researchers themselves. Since it is ultimately the authors’ hope to better highlight the similarities and differences between the published algorithms, it is incumbent on us to introduce a new classification which may conflict with those found in the literature. Whenever possible, we will indicate those conflicts in naming and justify the new terms.

The framework will consist of a pair of ellipses ${\mathcal {E}}_i$, ${\mathcal {E}}_j \subset {\mathbb {R}}^2$ in near-perfect contact, with or without overlap, as defined in Definition 6, but we shall discuss, when deemed necessary, the behavior of the algorithms for other configurations of the ellipses. A common feature of all algorithms presented here is that they compute two points ${\varvec{x}}_i$ and ${\varvec{x}}_j$ on the ellipses ${\mathcal {E}}_i$ and ${\mathcal {E}}_j$, respectively. Then, the *contact point* is usually defined as the midpoint between ${\varvec{x}}_i$ and ${\varvec{x}}_j$

93 $$\begin{aligned} {\varvec{x}}_c = \frac{1}{2} \big ({\varvec{x}}_i+{\varvec{x}}_j \big ), \end{aligned}$$

and allows one to compute a penetration (or separation) distance $d_{ij} = \Vert {\varvec{x}}_i - {\varvec{x}}_j\Vert$. Moreover, one can identify the contact normal as the unit vector from the *normal vector* ${\varvec{n}}_i ({\varvec{x}}_i)$ and the opposite normal vector ${\varvec{n}}_j({\varvec{x}}_j)$

94 $$\begin{aligned} {\varvec{n}}_c({\varvec{x}}_c) = \frac{ {\varvec{n}}_i({\varvec{x}}_i) - {\varvec{n}}_j({\varvec{x}}_j) }{\Vert {\varvec{n}}_i({\varvec{x}}_i) - {\varvec{n}}_j({\varvec{x}}_j) \Vert }. \end{aligned}$$

Alternatively, the contact normal could be computed as

95 $$\begin{aligned} {\varvec{n}}_c({\varvec{x}}_c) = \frac{ {\varvec{n}}_i({\varvec{x}}_c) - {\varvec{n}}_j({\varvec{x}}_c) }{\Vert {\varvec{n}}_i({\varvec{x}}_c) - {\varvec{n}}_j({\varvec{x}}_c) \Vert }. \end{aligned}$$

Another definition for the normal vector [34] has been provided as the unit vector along the line passing through the centers of the two tangent circles at ${\varvec{x}}_i$ and ${\varvec{x}}_j$ to the ellipses ${\mathcal {E}}_i$ and ${\mathcal {E}}_j$, respectively. In the case of the intersection of two ellipses, the normal at the point of contact is usually defined as the unit vector perpendicular to the line passing through the two points of the intersection set [42].

Finally, the *tangent line* is defined as the line perpendicular to ${\varvec{n}}_c$. As the relative positions of the ellipses approach that of perfect contact, then the points ${\varvec{x}}_i$ and ${\varvec{x}}_j$ coalesce, the distance $d_{ij}$ vanishes, and the tangent line aligns with the tangent to ${\mathcal {E}}_i$ at ${\varvec{x}}_i$ and the tangent to ${\mathcal {E}}_j$ at ${\varvec{x}}_j$.

### Intersection algorithm (IA)

The intersection algorithm was introduced by Rothenburg et al. in [42] and relies on estimating the points in the intersection set ${\mathcal {I}}_{ij} = {\mathcal {E}}_i \cap {\mathcal {E}}_j$ introduced in Definition 2. It is perhaps the most intuitive algorithm. We thus believe that it needs to be included in this survey, despite some of its drawbacks and limitations described below.

Lemma 2 shows that the intersection algorithm can be cast as the minimization Problem (39), which in the presence of overlap is the same as the minimum distance pair Problem (42) but without a constraint on the normals:

$$\begin{aligned} ({\varvec{x}}_i,{\varvec{x}}_j)  = \mathop {\hbox {argmin}}\limits _{({\hat{{\varvec{x}}}}_i,{\hat{{\varvec{x}}}}_j) \in {\mathcal {E}}_i \times {\mathcal {E}}_j} \Vert {\hat{{\varvec{x}}}}_i - {\hat{{\varvec{x}}}}_j\Vert  = \mathop {\hbox {argmin}}\limits _{({\hat{{\varvec{x}}}}_i,{\hat{{\varvec{x}}}}_j) \in {\mathcal {E}}_i \times {\mathcal {E}}_j} \frac{1}{2} \Vert {\hat{{\varvec{x}}}}_i - {\hat{{\varvec{x}}}}_j\Vert ^2. \end{aligned}$$

From a practical point of view, the method, in either case, consists in solving the system of equations:

96 $$\begin{aligned} \left\{ \begin{aligned}&f_i(x,y) = A_i x^2+B_i y^2+2C_i xy+2D_i x+2E_i y+F_i = 0,\\&f_j(x,y) = A_j x^2+B_j y^2+2C_j xy+2D_j x+2E_j y+F_j = 0, \end{aligned} \right. \nonumber \\ \end{aligned}$$

where $f_i$ and $f_j$ from Eq. (13) are the global geometric potentials of ${\mathcal {E}}_i$ and ${\mathcal {E}}_j$, respectively. A priori, we make no assumptions concerning the two ellipses. If ${\mathcal {E}}_i$ and ${\mathcal {E}}_j$ do not coincide, the system of equations can naturally be reduced into a single quartic equation in the first coordinate *x* (alternatively, in the second coordinate *y*):

97 $$\begin{aligned} \sum _{k=0}^4 a_k x^k = 0. \end{aligned}$$

where the coefficients $a_k$, $k=0,\ldots ,4$, of the polynomial can be explicitly given in terms of the coefficients of (96), see [42]. The quartic Eq. (97) admits at most four real roots $x_\ell$, $\ell =1,\ldots ,4$. Moreover, for each root $x_\ell$, one can use an explicit formula, see [42], to compute the corresponding coordinate $y_\ell$.

Different configurations of the ellipses will provide a different number of solutions to the quartic Eq. (97):

1. There are no real root. This is the case when the two ellipses are disjoint, i.e., ${\mathcal {I}}_{ij}=\varnothing$, whether they are disjoint with non-penetrating CoM or one ellipse lies inside the other (penetrating CoM). In this case, the algorithm does not provide information about the penetration distance.
2. There is one real root of multiplicity two. This is the case when there is a point at which the two ellipses are in perfect contact or when the *x*-coordinate of two distinct intersection points coincide. In the latter case, one cannot compute the *y*-coordinate of the two distinct points using the explicit formula [42]. Instead, given the common coordinate $x_\ell$, one should solve the quadratic equation in *y* using the global potential of ellipse ${\mathcal {E}}_i$,
  $$\begin{aligned} B_i y^2+ (2C_i x_\ell +2E_i) y + (A_i x_\ell ^2+2D_i x_\ell +F_i) = 0. \end{aligned}$$
  Alternatively, one could consider solving the quartic equation in *y* to obtain two distinct roots $y_\ell$. If a single root in *x* corresponds to a single point (*x*, *y*), then the separation distance and the normal can be computed easily.
3. There are two real distinct roots only. This is the most common configuration if the ellipses are in near-perfect contact with overlap. If ${\varvec{x}}_i$ and ${\varvec{x}}_j$ are two intersection points, the length $\Vert {\varvec{x}}_i-{\varvec{x}}_j\Vert$ provides an approximation of the length of the overlap. However, more work is needed to provide an estimate of the penetration, see more details in [42]. This case is illustrated in Fig. 13.
4. The four roots of the polynomial are all real, either all distinct, or two distinct roots and one root of multiplicity two, or two roots of multiplicity two. In practice, these cases are unlikely to occur in DEM applications since all are indicative of relatively large overlaps between ellipses.

Drawbacks in the intersection algorithm are that one needs to compute all real roots of the quartic polynomial and handle all specific cases depending on the number of roots and their multiplicity. Moreover, a major issue is that the algorithm is prone to numerical instabilities [42] in the case of very small overlaps between ellipses when estimating the two points $\{{\varvec{x}}_i, {\varvec{x}}_j \}$ and the normal direction. For these reasons, it is usually not the recommended approach for contact detection. Finally, the extension of the intersection algorithm to 3-D is increasingly more elaborate since the intersection set ${\mathcal {I}}_{ij}$ consists of 2-D ellipses on the surface of the ellipsoids. Given the weaknesses of this approach, we will not be providing further details on its extension to 3-D. However, we refer the reader to Ouadfel and Rothenburg [38] who proposed an algorithm in 3-D to find the contact point and contact normal.

### Geometric potential algorithms (GPA)

A geometric potential algorithm was first described by Ng et al. [31, 35] and has been further improved by Mustoe and Miyata [34]. GPAs are based on the symmetric pair of minimization problems (48)–(49) that we recall here for convenience

98 $$\begin{aligned} {\varvec{x}}_i= & {} \mathop {\hbox {argmin}}\limits _{{\varvec{x}}\in {\mathcal {E}}_i} f_j({\varvec{x}}), \end{aligned}$$

99 $$\begin{aligned} {\varvec{x}}_j= & {} \mathop {\hbox {argmin}}\limits _{{\varvec{x}}\in {\mathcal {E}}_j} f_i({\varvec{x}}). \end{aligned}$$

For any pair of ellipses in near-perfect contact the two problems have unique solutions, see Lemma 4.

**Fig. 14:**
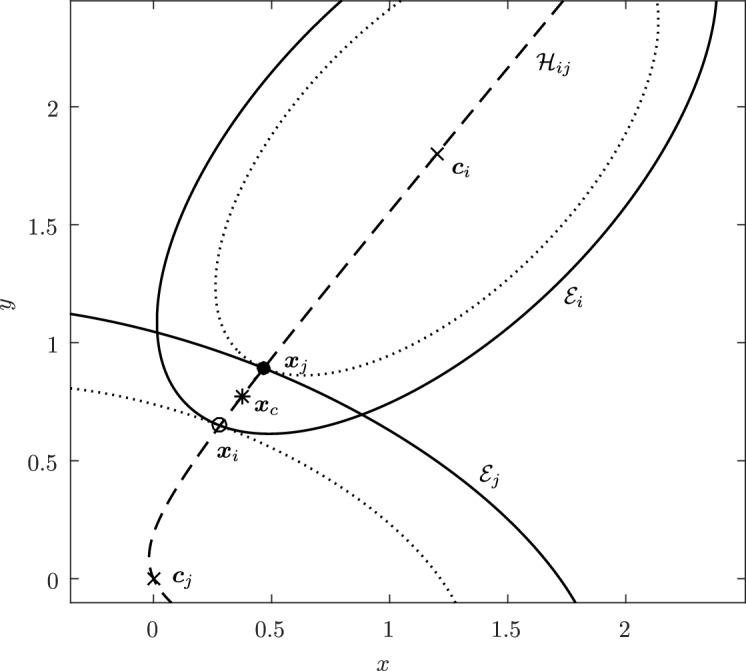
The points ${\varvec{x}}_i$ and ${\varvec{x}}_j$ obtained by geometric potential algorithms that provide the MPP, i.e., $({\varvec{x}}_i,{\varvec{x}}_j)$. Note that the contact point ${\varvec{x}}_c$ does not necessary belong to the gradient locus ${\mathcal {H}}_{ij}$

As the numerical experiments will show, GPAs are quite robust, even for pairs of ellipses with high aspect ratios, and at a computational cost competitive with other methods. However, two distinct problems must be solved and each problem generates up to four critical points from which the global minimum must be found. We present several numerical implementations of the GPA, including a Lagrangian and a parametric formulation. Penalization has not been found to be an effective means of solving (48)–(49) because the wide range of values of volume and aspect ratio make the choice of the penalization parameter difficult. In GPA algorithms, ${\varvec{x}}_i$ and ${\varvec{x}}_j$ are found by solving two distinct problems in the same way. Therefore, in the following, we discuss only the problem of finding the point ${\varvec{x}}_j$. The point ${\varvec{x}}_i$ is found in a similar fashion (Fig. 14).

**Fig. 15:**
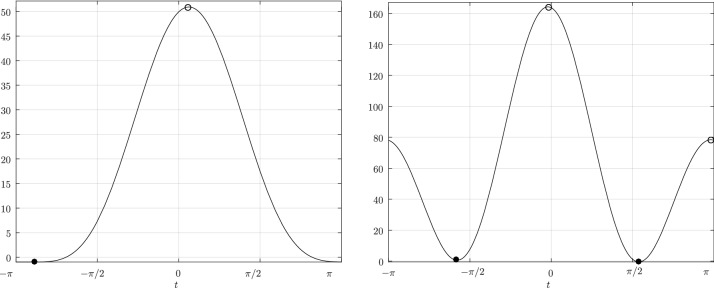
Graphs of the functions ${\widehat{f}}_i(t)$ (left) and ${\widehat{f}}_j(t)$ (right) associated with the ellipses ${\mathcal {E}}_i$ and ${\mathcal {E}}_j$ shown in Fig. 16. The function ${\widehat{f}}_i(t)$ has exactly one global minimum and one global maximum while the function ${\widehat{f}}_j(t)$ has two local minima and two local maxima in $[-\pi ,\pi [$. In both graphs, the local minima and maxima are represented by a dot ($\bullet$) and a disc ($\circ$), respectively

**Fig. 16:**
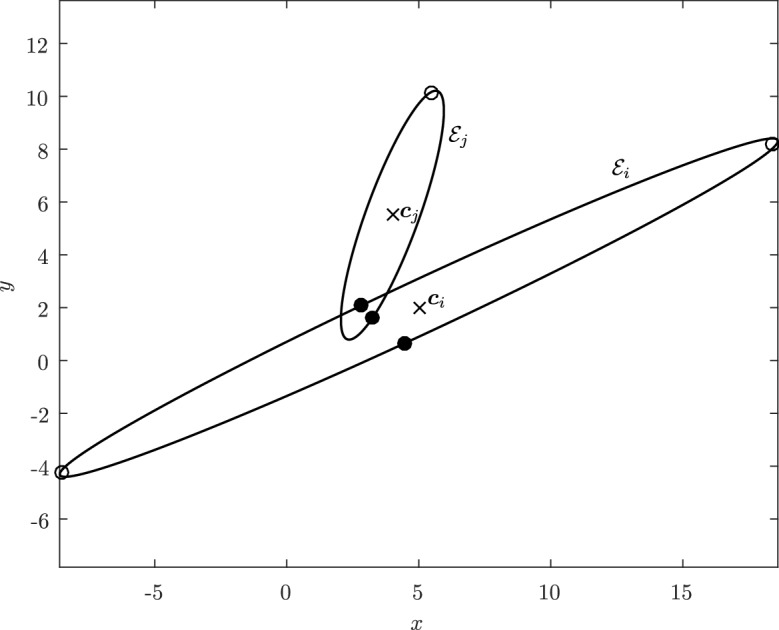
Configuration of the two ellipses ${\mathcal {E}}_i$ and ${\mathcal {E}}_j$ for which ${\widehat{f}}_j(t)$ has multiple local minima, as shown in Fig. 15. The points corresponding to local minima and local maxima are represented by a dot ($\bullet$) and a disc ($\circ$), respectively

1. L-GPA: The Lagrangian approach
  We describe the solution method proposed in [31] for solving the constrained minimization problem (99) to find ${\varvec{x}}_j$. We thus introduce the Lagrangian
  $$\begin{aligned} {\mathcal {L}}_j({\varvec{x}},\lambda ) = f_i({\varvec{x}}) - \lambda f_j({\varvec{x}}), \qquad \forall {\varvec{x}}\in {\mathbb {R}}^2,\ \forall \lambda \in {\mathbb {R}}, \end{aligned}$$
  where the constraint ${\varvec{x}}\in {\mathcal {E}}_j$, i.e., $f_j({\varvec{x}})=0$, is enforced via the Lagrange multiplier $\lambda$. Using the representation (1) for $f_k$, $k=i,j$, i.e.,
  $$\begin{aligned} {\mathcal {L}}_j({\varvec{x}},\lambda )&=({\varvec{x}}- {\varvec{c}}_i)^{T} {\mathcal {Q}}_i ({\varvec{x}}- {\varvec{c}}_i) \\&\quad -\lambda ({\varvec{x}}- {\varvec{c}}_j)^{T} {\mathcal {Q}}_j ({\varvec{x}}- {\varvec{c}}_j), \end{aligned}$$
  the stationary points $({\varvec{x}},\lambda )$ of ${\mathcal {L}}_i$ satisfy the system:
  100 $$\begin{aligned}&0 = \partial _{{\varvec{x}}} {\mathcal {L}}_i({\varvec{x}},\lambda ) = 2 {\mathcal {Q}}_i ({\varvec{x}}- {\varvec{c}}_i) - 2 \lambda {\mathcal {Q}}_j ({\varvec{x}}- {\varvec{c}}_j), \end{aligned}$$
  101 $$\begin{aligned}&0 = \partial _\lambda {\mathcal {L}}_i({\varvec{x}},\lambda ) = ({\varvec{x}}-{\varvec{c}}_j)^T {\mathcal {Q}}_j ({\varvec{x}}- {\varvec{c}}_j) - 1. \end{aligned}$$
  Isolating ${\varvec{x}}$ as a function of $\lambda$ from (100), we find
  102 $$\begin{aligned} {\varvec{x}}(\lambda ) = ({\mathcal {Q}}_i - \lambda {\mathcal {Q}}_j)^{-1}({\mathcal {Q}}_i {\varvec{c}}_i - \lambda {\mathcal {Q}}_j {\varvec{c}}_j). \end{aligned}$$
  Substituting (102) for ${\varvec{x}}(\lambda )$ in (101) produces a quartic polynomial in $\lambda$:
  103 $$\begin{aligned} \sum _{k=0}^4 a_k \lambda ^k = 0, \end{aligned}$$
  whose coefficients $a_k$ can be computed explicitly and can be found in [31]. Solving (103) provides at least two and at most four real roots $\lambda _\ell$, which in turn yields four candidate points ${\varvec{x}}(\lambda _\ell )$, $\ell =1,\ldots ,4$, according to (102). Then, the point ${\varvec{x}}_j$ is selected as the point that minimizes $f_i({\varvec{x}})$ among the points ${\varvec{x}}(\lambda _\ell )$. Extension of the Lagrangian approach to the case of ellipsoids is straightforward and results in a root-finding problem equivalent to (103), but involving a polynomial of degree 6 (see [31] for details).
2. P-GPA: The parametric approach
  We briefly describe here the approach proposed in [34] to solve the minimization problem (99). The main idea is to use the parametric representation (15) of an ellipse in order to eliminate the constraint from the minimization problem. This technique works in both two and three dimensions. Let the local potential ${\widehat{f}}_i$ of ${\mathcal {E}}_i$ be given as in (5), i.e.,
  104 $$\begin{aligned} {\widehat{f}}_i(\varvec{\xi }) = \varvec{\xi }^T {\mathcal {D}}_i \varvec{\xi }- 1, \end{aligned}$$
  where
  $$\begin{aligned} {\mathcal {D}}_i = \begin{bmatrix} 1/a_i^2 &{} 0 \\ 0 &{} 1/b_i^2 \end{bmatrix}. \end{aligned}$$
  The points ${\varvec{x}}$ on ${\mathcal {E}}_j$ can be parameterized in the local coordinate system by $\varvec{\zeta }(t) = (a_j \cos t, b_j \sin t)$ for $t \in [-\pi ,\pi [$. Using (4), the coordinates of ${\varvec{x}}$ in the local reference system $(O,\xi ,\eta )$ associated with ${\mathcal {E}}_i$ are then given by
  105 $$\begin{aligned} \varvec{\xi }(t) = {\mathcal {R}}_i^T \big [ ( {\mathcal {R}}_j \varvec{\zeta }(t) + {\varvec{c}}_j ) - {\varvec{c}}_i \big ] = {\mathcal {R}}_i^T {\mathcal {R}}_j \varvec{\zeta }(t) + \varvec{\xi }_0 \, ,\nonumber \\ \end{aligned}$$
  with $\varvec{\xi }_0 = (\xi _0,\eta _0) = {\mathcal {R}}_i^T ({\varvec{c}}_j - {\varvec{c}}_i).$ Replacing $\varvec{\xi }(t)$ in (104) by (105), one can then express ${\widehat{f}}_i$ as a function of parameter *t* only, i.e.,
  $$\begin{aligned} \begin{aligned} {\widehat{f}}_i(t)&= \varvec{\xi }(t)^T D_i \varvec{\xi }(t) - 1 = [{\mathcal {R}}_i^T {\mathcal {R}}_j \varvec{\zeta }(t) \\&\quad +\, \varvec{\xi }_0]^T {\mathcal {D}}_i [{\mathcal {R}}_i^T {\mathcal {R}}_j \varvec{\zeta }(t) + \varvec{\xi }_0] - 1, \end{aligned} \end{aligned}$$
  which can be reduced to
  $$\begin{aligned} {\widehat{f}}_i(t)= & {} \frac{ \big ( a_j \cos \theta _{ij} \cos t - b_j \sin \theta _{ij} \sin t + \xi _0 \big )^2}{a_i^2} \\&\quad +\, \frac{ \big ( b_j \cos \theta _{ij} \sin t + a_j \sin \theta _{ij} \cos t + \eta _0 \big )^2}{b_i^2}-1. \end{aligned}$$
  where $\theta _{ij} = \theta _j - \theta _i$, with $\theta _k$ for $k=i,j$, being the angle of rotation of ${\mathcal {R}}_k$, as defined in (2). It follows that the constrained minimization problem (99) for ${\varvec{x}}_j$ can be recast into the unconstrained minimization problem in one variable
  106 $$\begin{aligned} t_j = \mathop {\hbox {argmin}}\limits _{t \in [-\pi ,\pi [} {\widehat{f}}_i(t), \end{aligned}$$
  from which one would obtain the point ${\varvec{x}}_j \in {\mathcal {E}}_j$ by the change of variable (4):
  107 $$\begin{aligned} {\varvec{x}}_j = {\mathcal {R}}_j \varvec{\zeta }(t_j) + {\varvec{c}}_{j}. \end{aligned}$$
  The nonlinear functions ${\widehat{f}}_k(t)$, $k=i,j$, may have several extrema, which makes root-finding algorithms for $({\widehat{f}}_k)'(t)=0$ difficult. For example, in Fig. 15, we plot ${\widehat{f}}_i(t)$ and ${\widehat{f}}_j(t)$ associated with the pair of ellipses ${\mathcal {E}}_i$ and ellipse ${\mathcal {E}}_j$ shown in Fig. 16. In particular, we observe that the function ${\widehat{f}}_j(t)$ has two local minima and two local maxima. In order words, without any additional constraint, one needs to search for all extrema in order to find the global minimum.
3. M-GPA: The mapping approach
  Džiugys and Peters [12] proposed an alternative approach by introducing the mapping described in Sect. 4.2, which transforms ${\mathcal {E}}_i$ into a unit circle ${\hat{{\mathcal {C}}}}_i$ and the other ellipse ${\mathcal {E}}_j$ into an ellipse ${\hat{{\mathcal {E}}}}_j$ in its local reference system by the same mapping. Assuming the map has been applied and dropping the hat symbols, the circle and ellipse are then given by
  $$\begin{aligned} \begin{aligned}&f_i({\varvec{x}}) = ({\varvec{x}}-{\varvec{c}}_i)^T ({\varvec{x}}- {\varvec{c}}_i) - 1 = 0, \\&f_j({\varvec{x}}) = {\varvec{x}}^T {\mathcal {D}}_j {\varvec{x}}- 1 = 0. \end{aligned} \end{aligned}$$
  We first observe that the ${\mathcal {E}}_i$-norm is simply the Euclidean norm as the ellipse ${\mathcal {E}}_i$ is now reduced to the circle ${\mathcal {C}}_i$; see Lemma 13. It follows that the minimization problem (99) now reads
  108 $$\begin{aligned} {\varvec{x}}_j = \mathop {\hbox {argmin}}\limits _{{\varvec{x}}\in {\mathcal {E}}_j} f_i({\varvec{x}}) = \mathop {\hbox {argmin}}\limits _{{\varvec{x}}\in {\mathcal {E}}_j} \Vert {\varvec{x}}- {\varvec{c}}_i \Vert ^2. \end{aligned}$$
  In other words, the problem is to find the point ${\varvec{x}}_j$ on ellipse ${\mathcal {E}}_j$ that is the closest to ${\varvec{c}}_i$ in Euclidean norm.
  Džiugys and Peters [12, 13] proposed to solve the minimization problem (108) using two different approaches, one based on an iteration method and the other based on partial analytical results. We shall present only the latter approach below. Introducing the distance $\rho _i$ from the center of circle ${\mathcal {C}}_i$ to any point ${\varvec{x}}\in {\mathcal {E}}_j$
  109 $$\begin{aligned} \rho _i ({\varvec{x}})= \Vert {\varvec{x}}- {\varvec{c}}_i \Vert , \end{aligned}$$
  they explicitly enforce the constraint ${\varvec{x}}\in {\mathcal {E}}_j$ by rewriting $\rho _i$ as a function of *x* only, using the equation of ellipse ${\mathcal {E}}_j$
  $$\begin{aligned} \rho _i^2(x) = (x - c_{x})^2+ ( \pm \kappa \sqrt{a_j^2 - x^2} - c_{y})^2, \quad |x| \le a_j,\nonumber \\ \end{aligned}$$
  where $\kappa = b_j/a_j$ and ${\varvec{c}}_i = (c_{x},c_{y})$. The goal is therefore to find the global minimizer of the functional $\rho _i^2$, i.e.,
  $$\begin{aligned} x_j = \mathop {\hbox {argmin}}\limits _{ |x| \le a_j} \rho _i^2(x). \end{aligned}$$
  Finding the critical points of the minimization problem leads to solving a quartic equation in *x*:
  110 $$\begin{aligned} \sum _{k=0}^4 a_k x^k = 0 , \quad \text {with} \quad \left\{ \begin{aligned}&a_4 = (1-\kappa ^2)^2, \\&a_3 = - 2 c_{x} (1-\kappa ^2), \\&a_2 = c_{x}^2 + \kappa ^2 c_{y}^2 - a_j^2 (1-\kappa ^2)^2, \\&a_1 = 2 a_j^2 c_{x} (1-\kappa ^2), \\&a_0 = - a_j^2 c_{x}^2. \end{aligned} \right. \nonumber \\ \end{aligned}$$
  The quartic equation has a maximum of four roots, with possibly some conjugate complex roots, which can be found using an iterative nonlinear solver. Džiugys and Peters [12] also suggested an approach in which the solution procedure could be reduced to solving a cubic equation after introducing a special change of variable. Once the roots $x_\ell$, for $\ell =1,\ldots ,4$, are found, one can compute the corresponding $y_\ell$ coordinate, and the point ${\varvec{x}}_j$ is selected among the solutions ${\varvec{x}}_\ell = (x_\ell ,y_\ell )$ such that it minimizes $\rho _i({\varvec{x}})$ in (109).

### The constrained geometric potential algorithm (C-GPA)

We have seen in Lemma 4 that the additional constraints on the normals (50) and (51) are always satisfied at the minimum potential pair (MPP). Although the additional constraints on the normals are non-binding and could simply be ignored, it does significantly modify how the problems are solved and should further stabilize the problems. Ting and his collaborators [44–46] proposed to relax the constraints on the normals and to simply enforce that the points of the MPP belong to the so-called co-gradient locus. The constrained geometric potential algorithm can therefore be viewed as an extension of the GPAs. In other words, the point ${\varvec{x}}_j$ (resp. ${\varvec{x}}_i$) of the MPP is defined as the closest point with respect to the ${\mathcal {E}}_i$-norm (resp. ${\mathcal {E}}_j$-norm) to the center of the ellipse ${\mathcal {E}}_i$ (resp. ${\mathcal {E}}_j$) that intersects the co-gradient locus ${\mathcal {H}}_{ij}$ and the ellipse ${\mathcal {E}}_j$ (resp. ${\mathcal {E}}_i$). Formally, the problems can be recast as the constrained minimization problems:

111 $$\begin{aligned}&{\varvec{x}}_i = \mathop {\hbox {argmin}}\limits _{{\varvec{x}}\in {\mathcal {E}}_i \cap {\mathcal {H}}_{ij}} f_j({\varvec{x}}), \end{aligned}$$

112 $$\begin{aligned}&{\varvec{x}}_j = \mathop {\hbox {argmin}}\limits _{{\varvec{x}}\in {\mathcal {E}}_j \cap {\mathcal {H}}_{ij}} f_i({\varvec{x}}). \end{aligned}$$

We emphasize here that above problems are different from the minimization Problems (53) and (54). Indeed, the fact that a point ${\varvec{x}}$ belongs to the co-gradient locus ${\mathcal {H}}_{ij}$ does not necessarily imply that ${\varvec{n}}_i({\varvec{x}}) + {\varvec{n}}_j({\varvec{x}}) = {\varvec{0}}$ as one could also have ${\varvec{n}}_i({\varvec{x}}) = {\varvec{n}}_j({\varvec{x}})$.

**Fig. 17:**
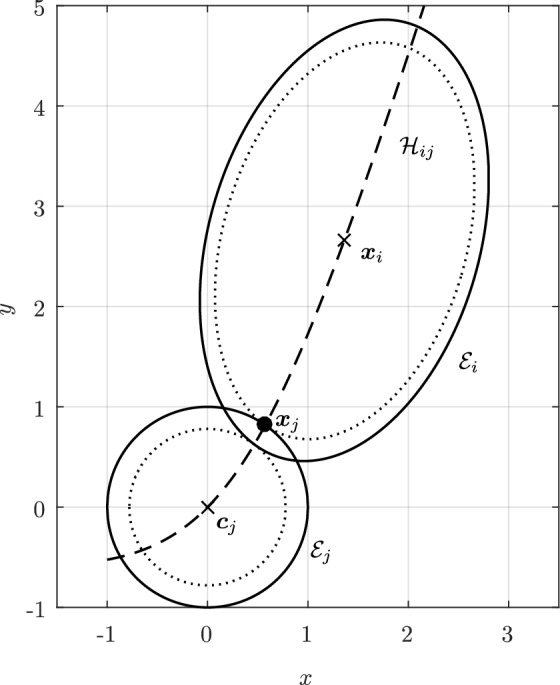
The two steps of the C-GPA algorithm are illustrated above. First, the mapping of Sect. 4.1 is applied to transform ellipses ${\mathcal {E}}_i$ and ${\mathcal {E}}_j$ to ellipse ${\mathcal {E}}_i$ and the unit circle ${\mathcal {C}}_j$ with center at the origin. Second, the solution of Problem (112) provides the point ${\varvec{x}}_j$ as the closest point to ellipse ${\mathcal {E}}_i$ among the points that intersect co-gradient locus ${\mathcal {H}}_{ij}$ and circle ${\mathcal {C}}_j$

The method proposed by Ting et al. in [45, 46] to solve problems (111) and (112) aims at finding the intersection points between each ellipse and the locus ${\mathcal {H}}_{ij}$ (the authors refer to ${\mathcal {H}}_{ij}$ as the *locus of common slope* in two dimensions). Since the two branches of the hyperbola may cross twice each ellipse, the solutions ${\varvec{x}}_i$ and ${\varvec{x}}_j$ are chosen as those that minimize $f_i$ and $f_j$ (equivalently, the ${\mathcal {E}}_i$- and ${\mathcal {E}}_j$-norms), respectively. In order to simplify the analysis to search for ${\varvec{x}}_i$, Ting et al. in [45, 46] proposed to consider the transformation of Sect. 4.1 that maps ${\mathcal {E}}_i$ into the unit circle ${\mathcal {C}}_i$ centered at the origin. Similarly, the second point ${\varvec{x}}_j$ is found by transforming the other ellipse ${\mathcal {E}}_j$ into the unit circle ${\mathcal {C}}_j$ while mapping the ellipse ${\mathcal {E}}_i$ into a new ellipse under the same transformation, see Fig. 17.

We briefly describe the algorithm in the case of a unit circle ${\mathcal {C}}_j$ and an ellipse ${\mathcal {E}}_i$ given by

$$\begin{aligned} \begin{aligned}&f_i({\varvec{x}}) = ({\varvec{x}}- {\varvec{c}}_i)^T {\mathcal {Q}}_i ({\varvec{x}}- {\varvec{c}}_i) - 1 = 0, \\&f_j({\varvec{x}}) = {\varvec{x}}^T {\varvec{x}}- 1 = 0. \end{aligned} \end{aligned}$$

The equation of the co-gradient locus ${\mathcal {H}}_{ij}$, see Eq. (68), is given in that case by

$$\begin{aligned} h({\varvec{x}}) = 4 {\varvec{x}}^T A {\mathcal {Q}}_i ({\varvec{x}}-{\varvec{c}}_i) = 0, \end{aligned}$$

where *A* is the anti-symmetric matrix (67). Using the notation of Sect. 2, the equation can be explicitly written as

113 $$\begin{aligned} h(x,y)&= 4C_i x^2 - 4C_i y^2 + 4(B_i - A_i) xy + 4 E_i x \nonumber \\&\quad - 4 D_i y = 0. \end{aligned}$$

The intersection of ${\mathcal {H}}_{ij}$ and ${\mathcal {C}}_j$ can be found by combining this equation with $x^2+y^2-1=0$ to obtain a single quartic equation in *x*

114 $$\begin{aligned} \sum _{k=0}^{4} a_k x^k = 0, \quad \text {with} \quad \left\{ \begin{aligned}&a_4 = (A_i - B_i)^2 + 4 C_i^2,\\&a_3 = 2 (A_i - B_i) D_i + 4 C_i E_i,\\&a_2 = - (A_i - B_i)^2 - 4 C_i^2 + D_i^2 + E_i^2,\\&a_1 = - 2 (A_i - B_i)D_i -2 C_i E_i,\\&a_0 = C_i^2 - D_i^2. \end{aligned} \right. \nonumber \\ \end{aligned}$$

Solving for (114) yields a maximum of four real roots $x_\ell$, $\ell =1,\ldots ,4$. Ting et al. [45, 46] compute the second coordinate $y_\ell$ from $x_\ell$ using the formula derived from the equations of the circle and ellipse:

115 $$\begin{aligned} y_\ell = \frac{ C_i ( 2 x_\ell ^2 - 1) + E_i x_\ell }{ (A_i - B_i) x_\ell + D_i}, \qquad \ell =1,2, \end{aligned}$$

as long as the denominator does not vanish for $x_\ell$. There may be an issue when the solution of (114) yields one real root of multiplicity two, which is the case when the two intersection points between the circle and the ellipse have the same coordinate $x_\ell$. Similarly to IA, the computation of the *y*-coordinate using (115) would result in one intersection point. One remedy is to use the equation of the circle to solve for $y_\ell$

$$\begin{aligned} y_\ell = \pm \sqrt{1 - x_\ell ^2}. \end{aligned}$$

### The steered geometric potential algorithm (S-GPA)

The authors proposed a novel approach [29] for contact detection between pairs of ellipses and ellipsoids combining existing techniques and new features. The algorithm belongs to the class of geometrical potential methods and, more specifically, involves solving the constrained minimization problem (112) after applying the mapping of Sect. 4.2. The efficiency of the algorithm thus relies on the following key ingredients: 1) the transformation (88) that maps the pair of ellipses into an ellipse centered at the origin and a unit circle; 2) the construction of an effective initial guess following the approach described in Sect. 4.3; 3) the use of Newton’s method for the root finding problem; and 4) the introduction of an additional constraint to guarantee convergence to the desired root.

Suppose now that the pair of ellipses, after transformation, consists of the ellipse ${\mathcal {E}}_j$ centered at the origin in its local coordinate system and of the unit circle ${\mathcal {C}}_i$ centered at ${\varvec{c}}_i=(c_x,c_y)$. We are thus looking for the point ${\varvec{x}}_j$ that satisfies:

$$\begin{aligned} {\varvec{x}}_j = \mathop {\hbox {argmin}}\limits _{{\varvec{x}}\in {\mathcal {E}}_j \cap {\mathcal {H}}_{ij}} f_i({\varvec{x}}). \end{aligned}$$

Recalling Eq. (89), a point ${\varvec{x}}=(x,y) \in {\mathcal {H}}_{ij}$ if it satisfies

$$\begin{aligned} h({\varvec{x}}) = ( a_j^2 - b_j^2 ) xy + b_j^2 c_y x - a_j^2 c_x y = 0. \end{aligned}$$

Moreover, ${\varvec{x}}\in {\mathcal {E}}_j$ can be expressed in parametric form with $t \in [-\pi ,\pi [$ as

$$\begin{aligned} {\varvec{x}}(t) = \begin{bmatrix} a_j \cos t \\ b_j \sin t \end{bmatrix}. \end{aligned}$$

Therefore, points ${\varvec{x}}$ belong to ${\mathcal {E}}_j \cap {\mathcal {H}}_{ij}$ if and only if they are the roots of the nonlinear function in *t*:

$$\begin{aligned} h(t) = ( a_j^2 - b_j^2 ) \sin t \cos t + b_j c_y \cos t - a_j c_x \sin t. \end{aligned}$$

We first note that the roots of *h*(*t*) are actually the critical points of the function $f_i({\varvec{x}})$ when ${\varvec{x}}\in {\mathcal {E}}_j$. Indeed, introducing the function *g*(*t*) defined for $t \in [-\pi ,\pi [$ as

$$\begin{aligned} g(t)= & {} \frac{1}{2} f_i ({\varvec{x}}(t)) = \frac{1}{2} \Vert {\varvec{x}}(t) - {\varvec{c}}_i \Vert ^2\\= & {} \frac{1}{2} \big [ (a_j \cos t - c_x)^2 +\, (b_j \sin t - c_y)^2 -1 \big ], \end{aligned}$$

it is straightforward to show that *h*(*t*) is associated with the derivative of *g*(*t*) as follows:

$$\begin{aligned} g'(t)= & {} - a_j \sin t ( a_j \cos t - c_x)+ b_j \cos t (b_j \sin t - c_y) \\= & {} - h(t). \end{aligned}$$

In other words, the roots of *h*(*t*) also satisfy $g'(t) = 0$, meaning that the points in ${\mathcal {E}}_j \cap {\mathcal {H}}_{ij}$ are either at a local minimum or at a local maximum of distance from ${\varvec{c}}_i$ when travelling along the ellipse ${\mathcal {E}}_i$. Since we have seen that the hyperbola ${\mathcal {H}}_{ij}$ may intersect ellipse ${\mathcal {E}}_j$ up to four times, we know that there exist up to four roots of *h*(*t*). The point that corresponds to the global minimum of *g*(*t*), and hence $f_i({\varvec{x}}(t))$, is thus the point ${\varvec{x}}_j= {\varvec{x}}(t_j)$ that we are looking for. The scalar function *h*(*t*) is clearly continuous on $t\in [-\pi ,\pi [$ but obviously non-convex. If one wants to use the second-order Newton’s method to find the roots of *h*(*t*), one needs to define an accurate initial guess point and possibly an additional constraint to ensure that the method converges to the desired root $t_j$, which will provide the actual solution ${\varvec{x}}_j$ to Problem (112). The initial guess point provided by the approach described in Sect. 4.3, although generally accurate, is not guaranteed to be located inside the basin of attraction of $t_j$, in which case the method will converge to another root of *h*(*t*).

Among the roots of the function *h*(*t*), only $t_j$ satisfies ${\varvec{n}}_i({\varvec{x}}(t_j)) \cdot {\varvec{n}}_j({\varvec{x}}(t_j)) < 0$ or, in an equivalent manner, $\nabla f_i({\varvec{x}}(t_j)) \cdot \nabla f_j({\varvec{x}}(t_j))< 0$; see [29] for a formal justification. This motivates us to introduce the function *q*(*t*), defined on $[-\pi ,\pi [$, as

$$\begin{aligned} q(t)= & {} \frac{1}{4} \nabla f_i({\varvec{x}}(t)) \cdot \nabla f_j({\varvec{x}}(t)) \nonumber \\= & {} \frac{x(t)}{a_i^2} (x(t) - c_x) +\, \frac{y(t)}{b_i^2} (y(t) - c_y)\nonumber \\= & {} 1 - \frac{c_x}{a_i} \cos t - \frac{c_y}{b_i} \sin t, \end{aligned}$$

and the constraint set

116 $$\begin{aligned} {\mathcal {S}}= \{ t \in [-\pi ,\pi [;\ q(t) < 0 \}. \end{aligned}$$

Therefore, the solution ${\varvec{x}}_j$ to Problem (112) can be obtained by searching for the **unique** root $t_j$ of *h*(*t*) that satisfies the constraint $t_j \in {\mathcal {S}}$. Using Newton’s method, we check that each new iterate belongs to ${\mathcal {S}}$. If not, we apply one iteration of the line search method to get closer to $t_j$, and we assume back in ${\mathcal {S}}$, hence the name chosen for this approach.

Extension of the algorithm to the case of pairs of ellipsoids is straightforward and is described in [29]. The advantage of this approach is that only the global solution to the minimization problem is actually computed and the use of the Newton’s method and the choice of the initial guess point make it very efficient, as shown in the numerical examples of Sect. 6.5.

### The common normal algorithm (CNA)

The common normal algorithm (CNA), first introduced by Lin et al. [31] in the case of ellipsoids, aims at rewriting the condition on the normals (43) satisfied by the MDP (42) into a system of solvable equations for the points ${\varvec{x}}_i \in {\mathcal {E}}_i$ and ${\varvec{x}}_j \in {\mathcal {E}}_j$. We recall that the condition on the normals, namely

117 $$\begin{aligned} {\varvec{n}}_i ({\varvec{x}}_i) + {\varvec{n}}_j ({\varvec{x}}_j) = {\varvec{0}}, \end{aligned}$$

is a natural property of the minimum distance pair of closest points $({\varvec{x}}_i,{\varvec{x}}_j) \in {\mathcal {E}}_i \times {\mathcal {E}}_j$ when the ellipses are disjoint, but the constraint is actually necessary when the two ellipses overlap, as the minimization problem (42) without the constraint on the normals leads in this case to the intersection set. Our objective in this section is to re-interpret the original formulation of the CNA [31] as a minimization problem.

Let ${\mathcal {E}}_i$ and ${\mathcal {E}}_j$ be two arbitrary ellipses. For any given point ${\varvec{x}}_i$ on ${\mathcal {E}}_i$, one can always identify a unique point ${\varvec{x}}_j$ on ${\mathcal {E}}_j$ such that ${\varvec{n}}_j ({\varvec{x}}_j) + {\varvec{n}}_i ({\varvec{x}}_i) = {\varvec{0}}$. This implies that the set of constraints $({\varvec{x}}_i,{\varvec{x}}_j) \in {\mathcal {E}}_i \times {\mathcal {E}}_j$ and ${\varvec{n}}_i ({\varvec{x}}_i) + {\varvec{n}}_j ({\varvec{x}}_j) = {\varvec{0}}$ provides an underdetermined system and, more precisely, one additional scalar constraint is required. Lin and Ng [31] proposed to consider that the unit vector going from ${\varvec{x}}_i$ toward ${\varvec{x}}_j$ be also equal to the normal vectors ${\varvec{n}}_j$ and $-{\varvec{n}}_i$. Introducing the unit vector

118 $$\begin{aligned} {\varvec{n}}({\varvec{x}}_i,{\varvec{x}}_j) = \frac{{\varvec{x}}_j - {\varvec{x}}_i}{\Vert {\varvec{x}}_j - {\varvec{x}}_i \Vert }, \end{aligned}$$

the problem of finding ${\varvec{x}}_i$ and ${\varvec{x}}_j$ would then consist in the following set of equations:

119 $$\begin{aligned} \left\{ \begin{aligned}&f_i({\varvec{x}}_i)=0,\\&f_j({\varvec{x}}_j)=0,\\&{\varvec{n}}_j ({\varvec{x}}_j) + {\varvec{n}}_i ({\varvec{x}}_i) = {\varvec{0}},\\&{\varvec{n}}_j ({\varvec{x}}_j) - {\varvec{n}}_i ({\varvec{x}}_i) = 2 {\varvec{n}}({\varvec{x}}_i,{\varvec{x}}_j). \end{aligned} \right. \end{aligned}$$

**Fig. 18:**
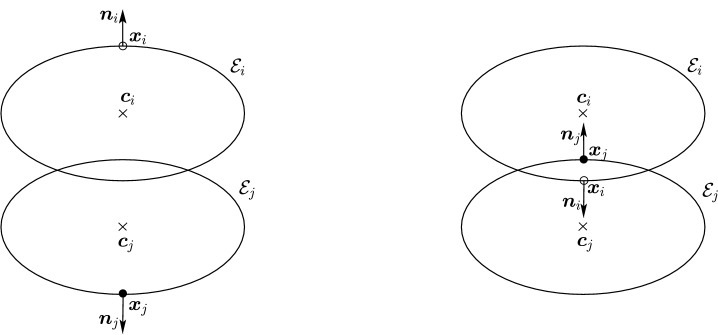
Example of a configuration of two ellipses with overlap for which the set of equations (119) leads to two solution pairs $({\varvec{x}}_i,{\varvec{x}}_j)$

Unfortunately, the problem above presents a few issues, which can be clearly described in the case of two circles: (1) If the two circles are disjoint, the problem has a unique solution pair, but the distance between the two points ${\varvec{x}}_i$ and ${\varvec{x}}_j$ reaches a maximum rather than a minimum, meaning that it is not really the pair of points that one is looking for; (2) if the two circles overlap, the problem admits two solutions, one solution pair for which the distance between the two points is a minimum and the other for which the distance is a maximum; (3) moreover, if the overlap between the two circles becomes very small, the calculation of the vector ${\varvec{n}}({\varvec{x}}_i,{\varvec{x}}_j)$ defined in (118) becomes problematic and the problem is ill-posed in the limit case of perfect contact. We illustrate in Fig. 18 the existence of two solutions to the system of equations (119).

**Fig. 19:**
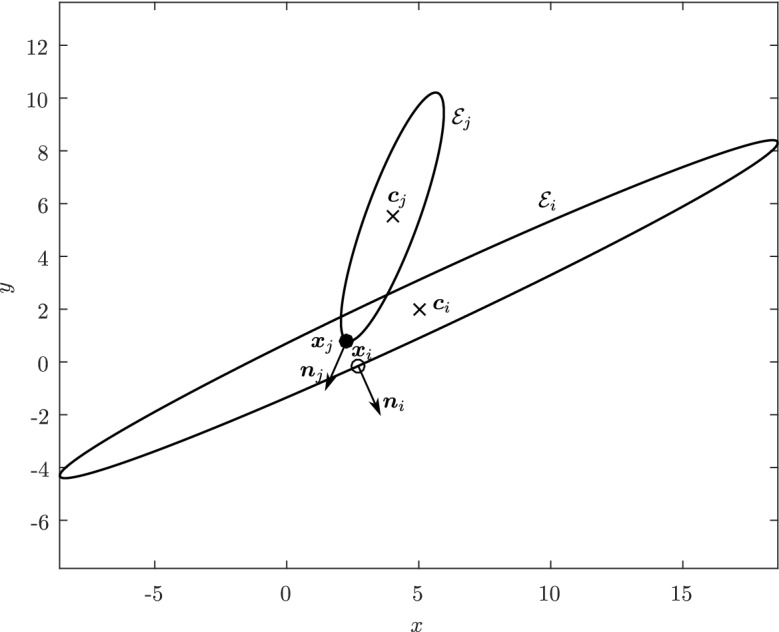
The pair $({\varvec{x}}_i,{\varvec{x}}_j)$ is found by solving (120). The normal vectors are obtained as ${\varvec{n}}_i({\varvec{x}}_i)=(0.410,-0.912)$ and ${\varvec{n}}_j({\varvec{x}}_j)=(-0.410,-0.912)$. In other words, the condition on the normals (117) is satisfied only with respect to the *x*-component as the *y*-components of the normal vectors are equal rather than being opposite. This explains that the system of equations (120) leads to an incorrect solution

Moreover, the system (119) consists of six nonlinear equations for the four variables ${\varvec{x}}_i = (x_i,y_i)$ and ${\varvec{x}}_j = (x_j,y_j)$. In order to circumvent the issue, Lin and Ng [31] proposed to consider only the following four equations (adapting the method described in 3-D in [31] to the 2-D case):

120 $$\begin{aligned} \left\{ \begin{aligned}&f_i({\varvec{x}}_i)=0,\\&f_j({\varvec{x}}_j)=0,\\&\frac{x_j-x_i}{\Vert {\varvec{x}}_j - {\varvec{x}}_i \Vert } = - \frac{1}{\Vert \nabla f_i\Vert } \frac{\partial f_i}{\partial x}({\varvec{x}}_i), \\&\frac{x_j-x_i}{\Vert {\varvec{x}}_j - {\varvec{x}}_i \Vert } = + \frac{1}{\Vert \nabla f_j\Vert } \frac{\partial f_j}{\partial x}({\varvec{x}}_j). \end{aligned} \right. \end{aligned}$$

Unfortunately, this arbitrary reduction in the number of equations has the effect of introducing additional solutions, as shown in Fig. 19. This is due to the fact that one should consider the direction of the normal vectors rather than simply equating their components in the *x*-direction, as done in (120). For all these reasons, the common normal algorithm is deemed inappropriate for finding contact points between ellipses or ellipsoids. As a final remark, the constrained minimization problem associated with Problem (119) can be cast as

121 $$\begin{aligned} ({\varvec{x}}_i,{\varvec{x}}_j) = \mathop {\hbox {argmin}}\limits _{\begin{array}{c} ({\hat{{\varvec{x}}}}_i,{\hat{{\varvec{x}}}}_j) \in {\mathcal {E}}_i \times {\mathcal {E}}_j \\ {\varvec{n}}_i({\hat{{\varvec{x}}}}_i) + {\varvec{n}}_j({\hat{{\varvec{x}}}}_j) = {\varvec{0}} \end{array}} \Vert {\varvec{n}}_j ({\hat{{\varvec{x}}}}_j) - {\varvec{n}}_i ({\hat{{\varvec{x}}}}_i) - 2 {\varvec{n}}({\hat{{\varvec{x}}}}_i,{\hat{{\varvec{x}}}}_j) \Vert ^2.\nonumber \\ \end{aligned}$$

We note that the above problem is actually similar to the minimization problem (47) except for the choice of the objective function. We now elaborate on the closest co-normal algorithm.

### The closest co-normal algorithm (CCA)

The algorithm was proposed in [53] as a variation of the common normal algorithm. The problem can be formulated as the closest co-normal minimization problem (47) that we recall here for convenience:

122 $$\begin{aligned} ({\varvec{x}}_i,{\varvec{x}}_j) = \mathop {\hbox {argmin}}\limits _{\begin{array}{c} ({\hat{{\varvec{x}}}}_i,{\hat{{\varvec{x}}}}_j) \in {\mathcal {E}}_i \times {\mathcal {E}}_j \\ {\varvec{n}}_i({\hat{{\varvec{x}}}}_i) + {\varvec{n}}_j({\hat{{\varvec{x}}}}_j) = {\varvec{0}} \end{array}} \Vert {\hat{{\varvec{x}}}}_i - {\hat{{\varvec{x}}}}_j \Vert ^2. \end{aligned}$$

This problem with constraints can be solved by various minimization methods, e.g., Lagrangian method. However, the authors in [53] proposed an original approach by parameterizing the distance $\Vert {\varvec{x}}_j - {\varvec{x}}_i \Vert$ in terms of a single parameter $t \in [-\pi ,\pi [$. The main idea relies on the fact that there exists a bijection between a point on an ellipse and the normal vector to the ellipse at that point. Indeed, let each ellipse ${\mathcal {E}}_k$, $k = i$ or *j*, be defined in its own local reference system as

123 $$\begin{aligned} {\widehat{f}}_k(\varvec{\xi })= \varvec{\xi }^T {\mathcal {D}}_k \varvec{\xi }-1 = 0. \end{aligned}$$

The outward unit normal vector at $\varvec{\xi }$ to ${\mathcal {E}}_k$ is then given by

$$\begin{aligned} {\hat{{\varvec{n}}}}_k (\varvec{\xi }) = \frac{\nabla {\widehat{f}}_k}{\Vert \nabla {\widehat{f}}_k\Vert } = \frac{2{\mathcal {D}}_k \varvec{\xi }}{\Vert \nabla {\widehat{f}}_k\Vert }, \end{aligned}$$

which implies that

124 $$\begin{aligned} \varvec{\xi }= \frac{\Vert \nabla {\widehat{f}}_k\Vert }{2} {\mathcal {D}}_k^{-1} {\hat{{\varvec{n}}}}_k (\varvec{\xi }). \end{aligned}$$

**Fig. 20:**
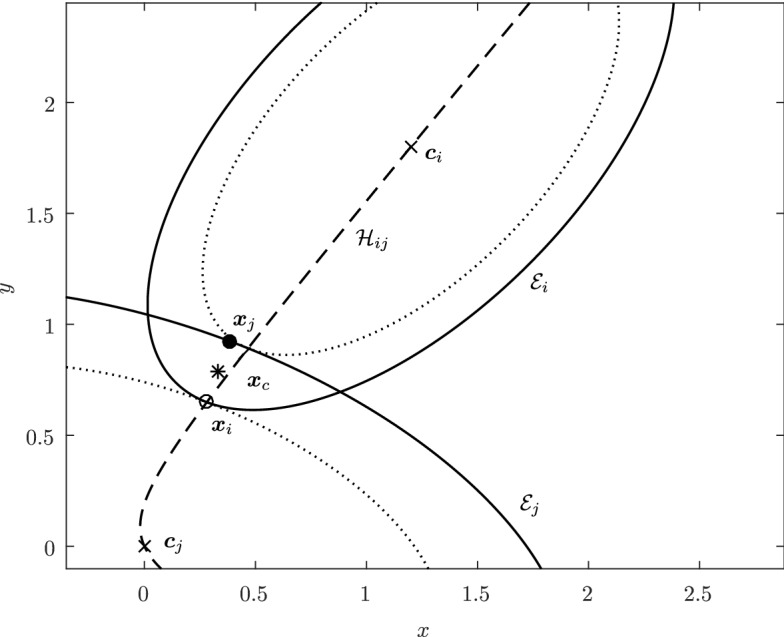
Solution pair $({\varvec{x}}_i,{\varvec{x}}_j)$ obtained by CNA solving the minimization problem (129). We observe that the points ${\varvec{x}}_i$ and ${\varvec{x}}_j$ are not necessary located on ${\mathcal {H}}_{ij}$

Since the normal vector ${\varvec{n}}_k$ in the global reference system is related to ${\hat{{\varvec{n}}}}_k$ by the rotation matrix ${\mathcal {R}}_k$ of angle $\theta _k$ (2) as

125 $$\begin{aligned} {\hat{{\varvec{n}}}}_k = {\mathcal {R}}_k^T {\varvec{n}}_k, \end{aligned}$$

we get, using (5),

126 $$\begin{aligned} {\varvec{x}}_k = {\mathcal {R}}_k \varvec{\xi }+ {\varvec{c}}_k = \frac{\Vert \nabla {\widehat{f}}_k\Vert }{2} {\mathcal {R}}_k {\mathcal {D}}_k^{-1} {\mathcal {R}}_k^T {\varvec{n}}_k + {\varvec{c}}_k. \end{aligned}$$

Moreover, substituting (124) for $\varvec{\xi }$ in (123) provides the new expression for the norm of the gradient:

127 $$\begin{aligned} \frac{1}{\Vert \nabla {\widehat{f}}_k\Vert ^2}&= \frac{1}{4} {\hat{{\varvec{n}}}}_k^T {\mathcal {D}}_k^{-1} {\hat{{\varvec{n}}}}_k \nonumber \\&= \frac{1}{4} \Vert {\mathcal {D}}_k^{-1/2} {\hat{{\varvec{n}}}}_k \Vert ^2 = \frac{1}{4} \Vert {\mathcal {D}}_k^{-1/2} {\mathcal {R}}_k^T {\varvec{n}}_k \Vert ^2, \end{aligned}$$

that is,

$$\begin{aligned} \Vert \nabla {\widehat{f}}_k\Vert = \frac{2}{ \Vert {\mathcal {D}}_k^{-1/2} {\mathcal {R}}_k^T {\varvec{n}}_k \Vert }, \end{aligned}$$

so that

128 $$\begin{aligned} {\varvec{x}}_k = \frac{{\mathcal {R}}_k {\mathcal {D}}_k^{-1} {\mathcal {R}}_k^T {\varvec{n}}_k }{\Vert {\mathcal {D}}_k^{-1/2} {\mathcal {R}}_k^T {\varvec{n}}_k \Vert } + {\varvec{c}}_k, \quad k = i,j. \end{aligned}$$

Using the above formula, one can now express the difference ${\varvec{x}}_j - {\varvec{x}}_i$ as

$$\begin{aligned} {\varvec{x}}_j - {\varvec{x}}_i= & {} \left[ \frac{{\mathcal {R}}_j {\mathcal {D}}_j^{-1} {\mathcal {R}}_j^T {\varvec{n}}_j }{\Vert {\mathcal {D}}_j^{-1/2} {\mathcal {R}}_j^T {\varvec{n}}_j \Vert } - \frac{{\mathcal {R}}_i {\mathcal {D}}_i^{-1} {\mathcal {R}}_i^T {\varvec{n}}_i }{\Vert {\mathcal {D}}_i^{-1/2} {\mathcal {R}}_i^T {\varvec{n}}_i \Vert } \right] \\&+ ({\varvec{c}}_j-{\varvec{c}}_i). \end{aligned}$$

In order to apply the constraint ${\varvec{n}}_i + {\varvec{n}}_j = {\varvec{0}}$, one can introduce the common direction ${\varvec{n}}$, parameterized with respect to $t \in [-\pi ,\pi [$, i.e.,

$$\begin{aligned} {\varvec{n}}(t) = \begin{bmatrix} \cos t \\ \sin t \end{bmatrix}, \end{aligned}$$

such that ${\varvec{n}}(t) = {\varvec{n}}_j = - {\varvec{n}}_i$. Therefore,

$$\begin{aligned}&{\varvec{x}}_j(t) - {\varvec{x}}_i(t) \\&\quad = \left[ \frac{{\mathcal {R}}_j {\mathcal {D}}_j^{-1} {\mathcal {R}}_j^T }{\Vert {\mathcal {D}}_j^{-1/2} {\mathcal {R}}_j^T {\varvec{n}}(t) \Vert } + \frac{{\mathcal {R}}_i {\mathcal {D}}_i^{-1} {\mathcal {R}}_i^T }{\Vert {\mathcal {D}}_i^{-1/2} {\mathcal {R}}_i^T {\varvec{n}}(t) \Vert } \right] {\varvec{n}}(t) + ({\varvec{c}}_j-{\varvec{c}}_i). \end{aligned}$$

The minimization problem (122) can thus be recast as that of finding the value $t_{ij} \in [-\pi ,\pi [$ such that

129 $$\begin{aligned} t_{ij} = \mathop {\hbox {argmin}}\limits _{t \in [-\pi ,\pi [} \Vert {\varvec{x}}_j(t) - {\varvec{x}}_i(t) \Vert ^2. \end{aligned}$$

**Fig. 21:**
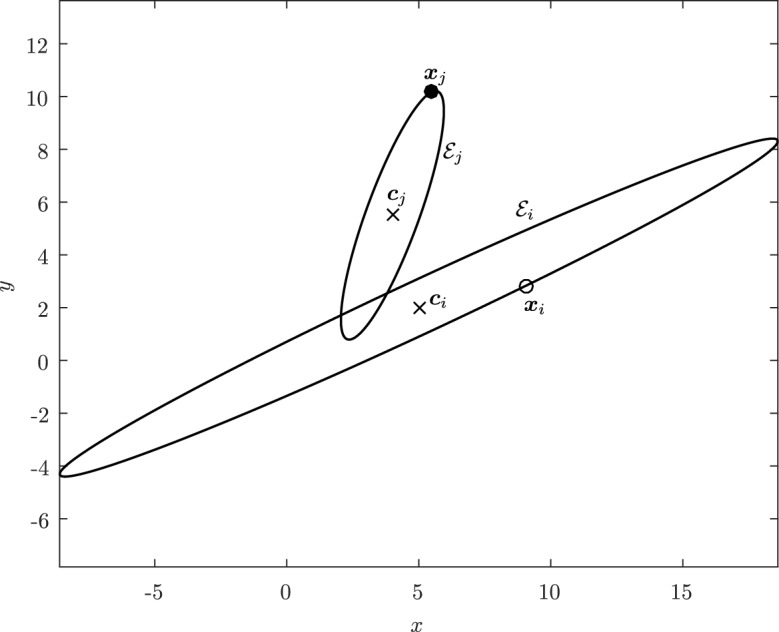
Solution pair $({\varvec{x}}_i,{\varvec{x}}_j)$ that corresponds to a local minimum of the minimization problem (129). The function $d(t) =\Vert {\varvec{x}}_j (t) - {\varvec{x}}_i(t) \Vert$ associated with this configuration of ellipses is plotted in Fig. 22

The original minimization problem with multiple constraints in the four variables $({\varvec{x}}_i, {\varvec{x}}_j)$ is now replaced by the unconstrained minimization problem (129) in the only variable *t*. We show in Fig. 20 the solution pair $({\varvec{x}}_i,{\varvec{x}}_j)$ obtained by solving (129) and observe that the two points do not necessarily belong to the co-gradient locus ${\mathcal {H}}_{ij}$. However, we note that for some configurations of ellipses, the distance $d(t) = \Vert {\varvec{x}}_j(t) - {\varvec{x}}_i(t) \Vert$ may be difficult to minimize as it may exhibit very flat regions and multiple extrema (up to two local minima and two local maxima), as shown in Fig. 21.

**Fig. 22:**
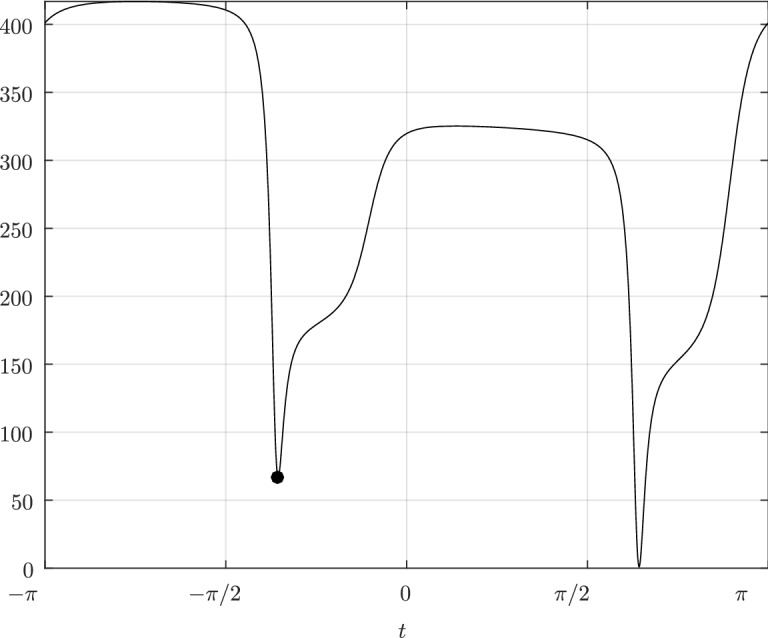
Plot of $d(t) = \Vert {\varvec{x}}_j (t) - {\varvec{x}}_i(t) \Vert$ for the pair of ellipses ${\mathcal {E}}_i$ and ${\mathcal {E}}_j$ shown in Fig. 21. The local minimizer of the distance function is represented with a dot ($\bullet$)

Finally, we note that the algorithm extends straightforwardly to the case of ellipsoids by considering the following parameterization of the normal vector:

130 $$\begin{aligned} {\varvec{n}}(u,v) = \begin{bmatrix} \cos {u} \cos {v} \\ \sin {u} \cos {v} \\ \sin {v} \end{bmatrix}, \quad u \in [-\pi ,\pi [,\ v \in [0,\pi ].\nonumber \\ \end{aligned}$$

## Numerical results

The objective of this section is twofold. First, it is to provide examples where one can observe the significant differences between the contact point associated with the intersection set (IS), the MDP, and the MPP. Second, it is to provide direct comparisons in accuracy and efficacy between the different contact detection algorithms presented in Sect. 5. In fact, the existing literature only provides a few comparisons between existing algorithms, mostly on a few pairs of ellipses, and those comparisons never emphasize how different the contact points from different algorithms may be. In practice, comparisons are difficult because

(i) algorithms based on different minimization problems have fundamentally different solutions;
(ii) some algorithms may be based on root finding of scalar polynomial equations, others on large coupled nonlinear systems, while others may utilize a key transformation to normalized coordinate systems. These choices dramatically affect accuracy and cost, how they are initialized, and even how accuracy is measured;
(iii) the wide variety of numerical techniques deployed imply that the computational efficiency is highly dependent on specific implementation details (code optimization, language strengths, or platform choice);
(iv) some algorithms have weaknesses in robustness, or may not be able to exploit prior accurate estimates of contacts, which render them undesirable in certain applications.

These difficulties will be circumvented by focusing mostly on the underlying minimization problems driving the algorithms and then dedicating the last subsection to applying the algorithms on a randomly generated set of pairs of ellipses in close or almost perfect contact.

The first four examples, detailed in Sects. 6.1–6.4, will present pairs of ellipses for which the differences are attributable solely to the minimization problem on which they are based. This implies that the computational cost will be ignored and all the algorithms will be run with a high tolerance in order to provide to machine precision the *exact* solution to the minimization problem. For these first four test problems, none of the contact detection algorithms failed, so we could focus only on the minimization problem.

**Table 1:** The two ellipses ${\mathcal {E}}_i$ and ${\mathcal {E}}_j$ of Example 6.1

	*a*	*b*	${\varvec{c}}$	$\theta$	*a*/*b*
Ellipse ${\mathcal {E}}_i$	10	4.1	(0, 0)	$-\,0.5$	2.44
Ellipse ${\mathcal {E}}_j$	10	4.1	(4, 5)	$-\,1.0$	2.44

**Table 2:** The contact points ${\varvec{x}}_c$ and their normal vectors ${\varvec{n}}_c$ for the different contact detection algorithms in Example 6.1

Algorithm	${\varvec{x}}_i$	${\varvec{x}}_j$	${\varvec{x}}_c$	${\varvec{n}}_i$	${\varvec{n}}_j$	${\varvec{n}}_c$
IA	(8.977, − 3.611)	(− 0.619, 4.816)	(4.179, 0.602)	(0.994, 0.112)	(− 0.890, − 0.455)	(0.660, 0.751)
P-GPA	(4.497, 2.002)	$(2.778, -\,0.154)$	(3.638, 0.923)	(0.587, 0.809)	$(-\,0.744, -\,0.668)$	(0.669, 0.743)
L-GPA	(4.497, 2.002)	$(2.778, -\,0.154)$	(3.638, 0.923)	(0.587, 0.809)	$(-\,0.744, -\,0.668)$	(0.669, 0.743)
M-GPA	(4.497, 2.002)	$(2.778, -\,0.154)$	(3.638, 0.923)	(0.587, 0.809)	$(-\,0.744, -\,0.668)$	(0.669, 0.743)
C-GPA	(4.497, 2.002)	$(2.778, -\,0.154)$	(3.638, 0.923)	(0.587, 0.809)	$(-\,0.744, -\,0.668)$	(0.669, 0.743)
CNA	(6.108, 0.703)	$(4.118, -\,1.504)$	$(5.113, -\,0.401)$	(0.670, 0.743)	$(-\,0.670, -\,0.743)$	(0.670, 0.743)
CCA	(6.108, 0.703)	$(4.118, -\,1.504)$	$(5.113, -\,0.401)$	(0.670, 0.743)	$(-\,0.670, -\,0.743)$	(0.670, 0.743)

In Sect. 6.5, our objective is to highlight the algorithmic aspects of the resolution of the different minimization problems. To accomplish this, ten thousand pairs of ellipses in close or almost perfect contact are randomly generated according to an algorithm detailed in another publication by the authors [29]. For each algorithm, the total computational time is found and then further subdivided into its different steps; see Tables 9 and 10. This allows us to address issues (ii) and (iii). For those tests, two different tolerances were used. Overall, Sect. 6.5 indicates that the S-GPA is the fastest, but because of the issues just mentioned above any comparison between algorithms comes with significant caveats.

### Comparison of the intersection set (IS), the MDP, and the MPP

**Fig. 23:**
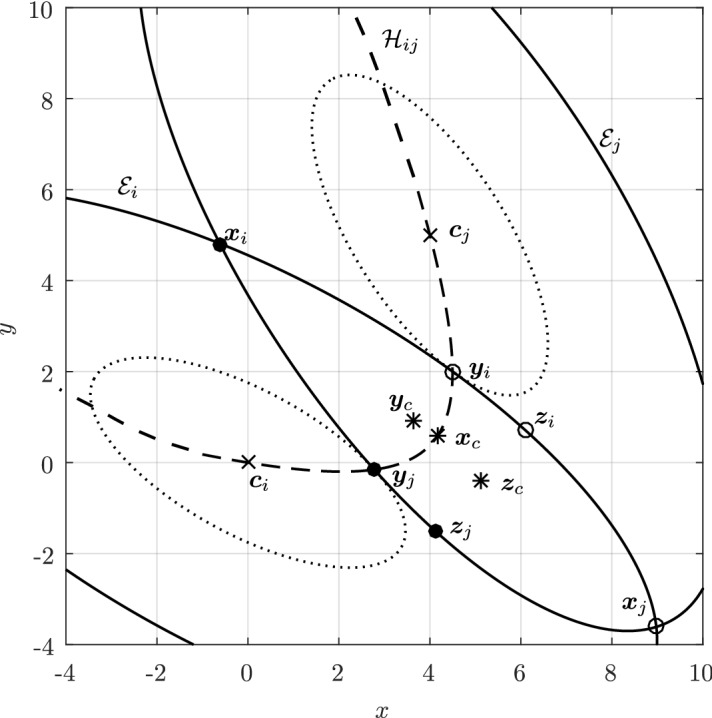
Location of the contact points using different contact detection algorithms: the intersection set is the pair $({\varvec{x}}_i,{\varvec{x}}_j)$ with contact point ${\varvec{x}}_c$, the MPP is $({\varvec{y}}_i,{\varvec{y}}_j)$ with contact point ${\varvec{y}}_c$, and the MDP is $({\varvec{z}}_i,{\varvec{z}}_j)$ with contact point ${\varvec{z}}_c$. The co-gradient locus ${\mathcal {H}}_{ij}$ is drawn as a dashed line, and it traverses both centers ${\varvec{c}}_i$ and ${\varvec{c}}_j$. The dotted line presents the scaled ellipses that are tangent at the MPP

The objective of this example is to demonstrate that with strict tolerances, the contact point obtained by a given algorithm will depend only on the minimization problem which the algorithm attempted to solve. Following this remark, the numerical results of Sects. 6.2–6.4 will focus only on the different solutions associated with either the MDP or the MPP. Nevertheless, the example in this section is also interesting in its own right since it presents a pair of overlapping ellipses, given in Table 1, for which the intersection set $({\varvec{x}}_i,{\varvec{x}}_j)$, the minimum distance pair $({\varvec{z}}_i,{\varvec{z}}_j)$, and the minimum potential pair $({\varvec{y}}_i,{\varvec{y}}_j)$ are different. In particular, this example shows that the resulting contact points ${\varvec{x}}_c$, ${\varvec{z}}_c$, and ${\varvec{y}}_c$ will be distinct, as seen in Fig. 23. On the other hand, the normals at each of these three contact points are roughly the same, computed according to either (94) or (95). In general, we have observed the normals to be less sensitive to the choice of the algorithm than the estimates of the contact point.

The numerical results in Table 2 show that the MPP $({\varvec{y}}_i, {\varvec{y}}_j)$ is the same whether it is computed by the P-GPA, L-GPA, M-GPA, or the C-GPA. Similarly, using sufficiently high tolerances, we find that the estimated MDP is the same whether computed by the CNA (when CNA provides the correct answer) or the CCA.

Examining Fig. 23 and comparing it to the definition of the MDP and MPP, it is easy to explain the differences between the contact points ${\varvec{x}}_c$, ${\varvec{z}}_c$, and ${\varvec{y}}_c$. For example, the MPP $({\varvec{y}}_i,{\varvec{y}}_j)$ clearly belongs to the co-gradient locus, but their normals are different at each point. On the other hand, it is clear in Fig. 23 that the normals at the MDP $({\varvec{z}}_i,{\varvec{z}}_j)$ are opposite. The contact point ${\varvec{z}}_c$ is far from the co-gradient locus because both ellipses have roughly parallel surfaces along a large portion of the intersection $E_i \cap E_j$.

### Different MDP and MPP with the same contact points

In this example, we present a pair of ellipses, described in Table 3, for which the contact point ${\varvec{z}}_c$ of the MDP $({\varvec{z}}_i,{\varvec{z}}_j)$ and the contact point ${\varvec{y}}_c$ of the MPP $({\varvec{y}}_i,{\varvec{y}}_j)$ are the same, but the associated normals are different. This expands on the previous example because the pairs are different, yet lead to equal contacts. The fact that the normals are different but the contact points are the same implies that the choice of using the MDP or the MPP could lead to significantly different forces between ellipses in a DEM model. The example is quite simple because, as Table 3 shows, both ellipses have the same aspect ratio and the co-gradient locus degenerates to a straight line through both centers. The quartic equations (110) and (114) derived from M-GPA and C-GPA, respectively, degenerate in this case to quadratic equations, i.e., $a_4=a_3=a_1=0$. This implies that the mappings in Sects. 4.1 and 4.2, applied, respectively, in M-GPA and C-GPA, send both ellipses to circles. In those new coordinates, the contact is easy to compute analytically. Those analytic verifications show that the coincidence ${\varvec{z}}_c = {\varvec{y}}_c$ is exact, and not simply an artifact of the numerical algorithms.

**Fig. 24:**
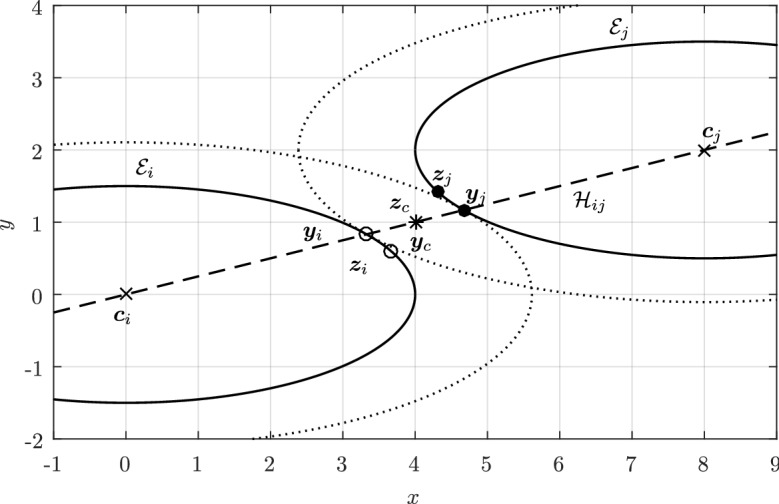
Two disjoint ellipses ${\mathcal {E}}_i$ and ${\mathcal {E}}_j$ with the same major and minor axis are aligned with *x*-axis. In this configuration, ${\mathcal {H}}_{ij}$ degenerates to a straight line passing through ${\varvec{c}}_i$ and ${\varvec{c}}_j$; see Corollary 10. The MDP $({\varvec{z}}_i,{\varvec{z}}_j)$ are not located on the co-gradient locus ${\mathcal {H}}_{ij}$ as the MPP $({\varvec{y}}_i,{\varvec{y}}_j)$. However, the contact points ${\varvec{z}}_c$ and ${\varvec{y}}_c$ are identical and are both located on the co-gradient locus ${\mathcal {H}}_{ij}$

We report in Table 4 and show in Fig. 24 the MDP and the MPP, obtained, respectively, by a GPA algorithm and by the CCA with high tolerance. It is a coincidence in this example that the normals are opposite, both for the MDP and for the MPP.

### Ellipses in perfect contact

**Fig. 25:**
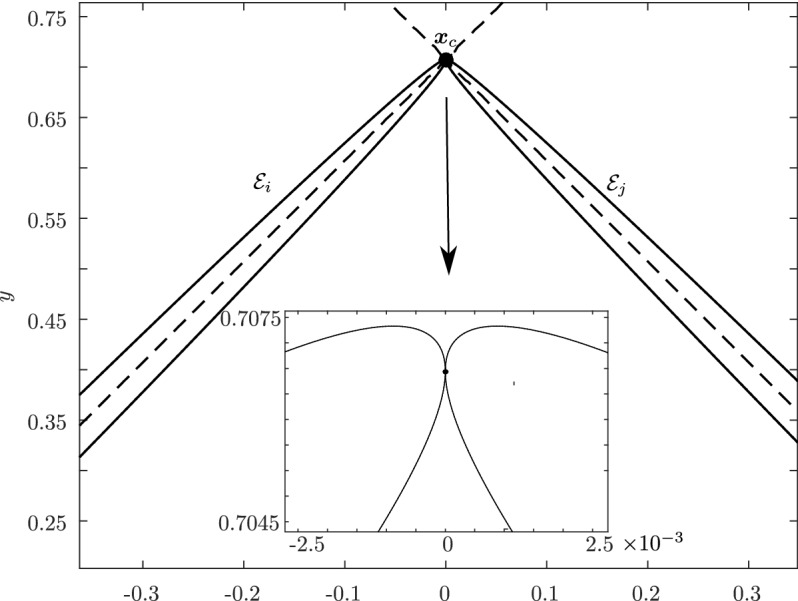
The location of the contact points ${\varvec{x}}_c$ is the same for both the MDP and MPP pairs

Following the work of Dziugys et al. [12, 13], we consider a pair of ellipses of high aspect ratio in perfect contact, according to Definition 4. This is a numerically challenging case, yet both the MDP and the MPP coincide mathematically in this case. As expected, all algorithms identify the same contact point. This perfect contact is illustrated in Fig. 25. The ellipses are described in Table 5, and the resulting MDP and MPP pairs are given in Table 6.

**Table 3:** The two ellipses ${\mathcal {E}}_i$ and ${\mathcal {E}}_j$ of Example 6.2

	*a*	*b*	${\varvec{c}}$	$\theta$	*a*/*b*
Ellipse ${\mathcal {E}}_i$	4.0	1.5	(0, 0)	0	2.67
Ellipse ${\mathcal {E}}_j$	4.0	1.5	(8, 2)	0	2.67

**Table 4:** The contact points ${\varvec{x}}_c$ and their normal vectors ${\varvec{n}}_c$ for the two classes of contact detection algorithms in Example 6.2

	GPA	CCA
${\varvec{x}}_i$	(3.328, 0.832)	(3.662, 0.604)
${\varvec{x}}_j$	(4.672, 1.168)	(4.338, 1.396)
$${\varvec{x}}_c$$	(4, 1)	(4, 1)
${\varvec{n}}_i({\varvec{x}}_i)$	(0.490, 0.872)	(0.649, 0.761)
${\varvec{n}}_j({\varvec{x}}_j)$	$(-\,0.490, -\,0.872)$	$(-\,0.649, -\,0.761)$
${\varvec{n}}_c({\varvec{x}}_c)$	(0.490, 0.872)	(0.649, 0.761)

### Ellipses with small overlap

**Fig. 26:**
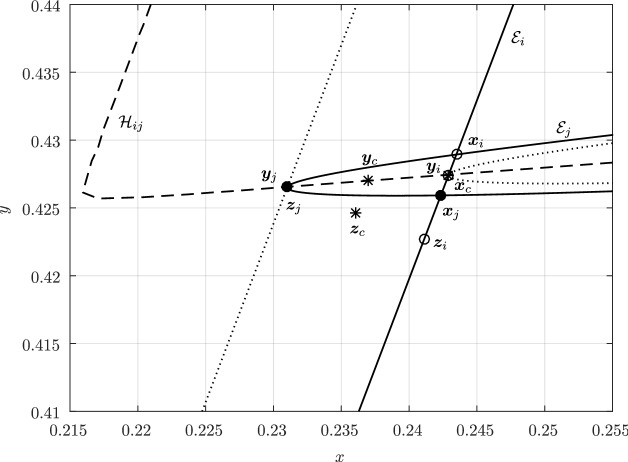
Locations of contact points using different contact detection algorithms: $({\varvec{x}}_i,{\varvec{x}}_j,{\varvec{x}}_c)$ are obtained by using IA, $({\varvec{y}}_i,{\varvec{y}}_j,{\varvec{y}}_c)$ are obtained by using GPA, and $({\varvec{z}}_i,{\varvec{z}}_j,{\varvec{z}}_c)$ are obtained by using CCA

In this challenging test, the two ellipses of high aspect ratio have a relatively innocuous overlap which, with respect to their size, one would not expect to cause trouble. Yet, as Fig. 26 shows, neither the intersection point ${\varvec{x}}_c$, nor the point ${\varvec{z}}_c$ associated with the MDP belongs to the intersection of both ellipses. However, the MPP produces a reasonable contact point ${\varvec{y}}_c$ inside the intersection between both ellipses $E_i \cap E_j$. We insist here that these ellipses are not in near-perfect contact, that is according to Definition 6, because the two disks of radius ${\underline{\rho }}_i$ and ${\underline{\rho }}_j$, tangent to ${\varvec{y}}_i$ and ${\varvec{y}}_j$, respectively, do not have non-penetrating CoM, as required by Theorem 7. The description of the ellipses is given in Table 7. The estimates of the contact points for both the IS, the MDP, and the MPP are given in Table 8. We note that the estimated normals are very close to each other.

**Table 5:** The two ellipses ${\mathcal {E}}_i$ and ${\mathcal {E}}_j$ of Example 6.3

	*a*	*b*	${\varvec{c}}$	$\theta$	*a*/*b*
Ellipse ${\mathcal {E}}_i$	1.0	0.025	$(-\,0.7073277, 0)$	0.753	40
Ellipse ${\mathcal {E}}_j$	1.0	0.025	(0.7073277, 0)	$-$ 0.753	40

**Table 6:** Contact points ${\varvec{x}}_c$ and normal vectors ${\varvec{n}}_c$ obtained by contact detection algorithms in Example 6.3

	GPA	CCA
${\varvec{x}}_i$	$(1.73 \times 10^{-8}, 0.706)$	$(1.73 \times 10^{-8}, 0.706)$
${\varvec{x}}_j$	$(-\,1.73 \times 10^{-8}, 0.706)$	$(-\,1.73 \times 10^{-8}, 0.706)$
${\varvec{x}}_c$	(0, 0.706)	(0, 0.706)
$${\varvec{n}}_i({\varvec{x}}_i)$$	(1, 0)	(1, 0)
${\varvec{n}}_j({\varvec{x}}_j)$	$(-\,1,0)$	$(-\,1,0)$
$${\varvec{n}}_c({\varvec{x}}_c)$$	(1, 0)	(1, 0)

### Large-scale tests

In contrast to the previous four sections, we attempt here to analyze and compare the accuracy and efficiency of the algorithms presented in Sect. 5. More precisely, we study the individual numerical approximations implemented in the algorithms used to solve the two main contact detection problems we identified, that is the MDP and the MPP, independent of the characteristics of the problems themselves studied in the previous four sections. As we argued in the introductory comments of this section, there are many reasons why comparisons between contact detection algorithms could be difficult. Nevertheless, as we will explain below, we can come to a limited number of conclusions by studying the behavior of these algorithms on a large sample of pairs of ellipses in close contact, and then separately considering the contributions to accuracy and efficiency of the individual components of the algorithm. The tests in this section are new to the literature and should help to establish benchmarks for comparisons between such algorithms for contact detection.

**Fig. 27:**
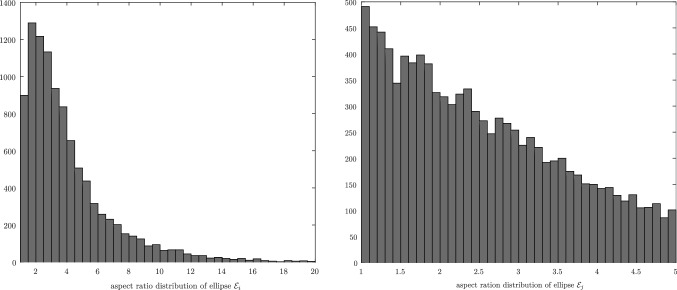
Distribution of the aspect ratio of the ellipses ${\mathcal {E}}_i$ (left) and ${\mathcal {E}}_j$ (right)

**Fig. 28:**
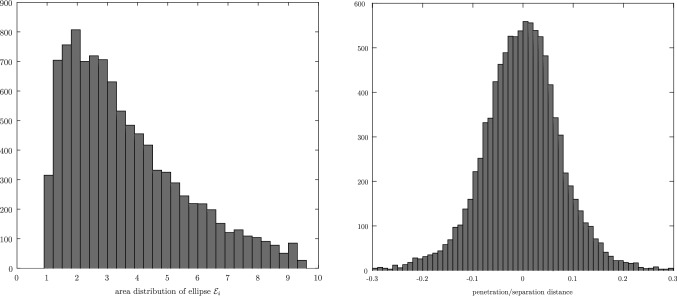
(Left) Distribution of the area of the ellipses ${\mathcal {E}}_i$. (Right) Distribution of the penetration/separation distance for the pairs of ellipses, where a positive (negative) distance represents a separation (penetration) distance

The first step was to generate a random set of 10,000 pairs of ellipses in almost or close contact, according to an algorithm described in a companion publication [29], and to apply to each pair of ellipses all algorithms (except IA) of Sect. 5. In order to reproduce pairs of ellipses one might encounter in DEM, the generating algorithm provided some control on the aspect ratio of each ellipse, on their relative orientation, on their relative closeness, and on the location of the contact point along the boundary of each ellipse. First of all, the first ellipse ${\mathcal {E}}_i$ was permitted to have maximum aspect ratio of $a/b=5$, while ellipse ${\mathcal {E}}_j$ was permitted to have an aspect ratio as large as $a/b=20$. Figure 27 presents the actual distribution of the aspect ratio *a*/*b* for ${\mathcal {E}}_i$ and ${\mathcal {E}}_j$. The generating algorithm assumes that $a_jb_j=1$ for ${\mathcal {E}}_j$, but the distribution of the area $\pi ab$ for ${\mathcal {E}}_i$ is randomly determined and shown in Fig. 28.

**Table 7:** The ellipses ${\mathcal {E}}_i$ and ${\mathcal {E}}_j$ of Example 6.4

	*a*	*b*	${\varvec{c}}$	$\theta$	*a*/*b*
Ellipse ${\mathcal {E}}_i$	7.0	0.025	(0.2350, 0.4770)	1.2077	280
Ellipse ${\mathcal {E}}_j$	7.0	0.025	(7.2113, 0.9515)	0.0750	280

**Table 8:** Contact point ${\varvec{x}}_c$ and normal vector ${\varvec{n}}_c$ obtained by different algorithms in Example 6.4

	IS	MPP	MDP
${\varvec{x}}_i$	(0.24348, 0.42893)	(0.24291, 0.42743)	(0.24111, 0.42270)
${\varvec{x}}_j$	(0.24234, 0.42592)	(0.23102, 0.42653)	(0.23102, 0.42653)
${\varvec{x}}_c$	(0.24291, 0.42743)	(0.23696, 0.42698)	(0.23606, 0.42462)
${\varvec{n}}_i({\varvec{x}}_i)$	$(0.934815,-\,0.355135)$	$(0.934815,-\,0.35513)$	$(0.934814,-\,0.35514)$
${\varvec{n}}_j({\varvec{x}}_j)$	$(0.012235,-\,0.999920)$	$(-\,0.934807,0.35515)$	$(-\,0.934814,0.35514)$
${\varvec{n}}_c({\varvec{x}}_c)$	$(0.934815,-\,0.355130)$	$(0.934810,-\,0.35514)$	$(0.934814,-\,0.35514)$

The algorithm generating these pairs is able to provide exactly one of the two points in the MPP, thus allowing us to estimate the relative error of the algorithms in the GPA family. On the other hand, for the IA and the MDP algorithms, estimates of error are not directly accessible. In any case, we are able to control the distribution of the penetration/separation distance, as shown in Fig. 28, supporting our claim that the pairs of ellipses in our tests were relatively close. Furthermore, the generation of the MPP for the pair of ellipses also allows one to uniformly select the second ellipse along the boundary of the first ellipse, thereby ensuring that we consider contact points both in the flatter or in the more curved regions of the boundary of the ellipses.

**Table 9:** Computational cost, number of iterations, and logarithmic error for different algorithms using a relative tolerance of $10^{-5}$

	L-GPA	P-GPA	M-GPA	C-GPA	S-GPA	CNA	CCA
Total computational cost (s)	10.79	8.24	15.22	13.76	1.96	107.58	22.03
Mapping	0	0	1.31	1.07	1.31	0	0
Initialization (focal points)	0	0	0	0	0.21	0	0
Initialization (golden search)	0	5.55	0	0	0	0	0
Root finding (Francis’ algorithm)	9.90	0	12.79	11.65	0	0	0
Root finding (Newton’s method)	0	0.09	0	0	0.09	0	0
Root finding (MATLAB functions)	0	0	0	0	0	101.22	17.47
Number of iterations							
Maximum	41	199	56	50	4	109	13
Minimum	11	15	5	5	1	4	1
Median	18	18	23	22	3	10	4
Standard deviation	3.27	7.31	8.31	8.11	0.68	17.90	2.07
Logarithmic error							
Maximum	$-\,5.17$	$-\,5.18$	$-\,5.05$	$-\,5.12$	$-\,5.12$		
Median	$-\,11.97$	$-\,10.84$	$-\,12.00$	$-\,12.46$	$-\,10.22$		
Standard deviation	2.14	1.65	2.43	2.39	2.61		

The numerical results of the experiments are summarized in Table 9, where a relative tolerance of $10^{-5}$ was used, and in Table 10, where a stricter relative tolerance of $10^{-9}$ was used. We will discuss the results of Table 10 later, since it mostly concerns the observed convergence and how it depends on the underlying numerical approximations. Table 9 presents for each of the seven algorithms of Sect. 5, the total computational time required for the resolution of the 10,000 pairs of ellipses, the statistics of the number of iterations required for the solution, and the statistics of the error in those approximate solutions. First of all, the error could only be measured for the algorithms estimating the MPP, since the algorithm generating the pairs only provided the exact MPP. This explains why the errors for the CNA and the CCA are not tabulated. The table also includes the number of iterations that were required to attain the desired relative tolerance, but one must be careful when comparing these values because the nature of the iterations in, say, the golden search algorithm, Newton’s method, or Francis’ algorithm is completely different. Finally, we have included the total computational time required to solve the 10,000 pairs of ellipses, as measured by the profiler in MATLAB. Although estimates of computational time in MATLAB are known to be somewhat variable, we have performed many such studies and found the estimates of computational time to be consistently reproducible to within $10\%$.

We now proceed to analyze the results of the experiments in Table 9, going from the most general to the most specific conclusions. First of all, we remark that the median error was roughly $10^{-11}$, that is several orders of magnitude lower than the chosen tolerance, showing that for most pairs of ellipses, the algorithms converged quickly to the MPP. The relatively low standard deviation further shows that the error was close to the relative tolerance of $10^{-5}$ for only a small subset of the pairs of ellipses. In Table 9, we immediately remark that the total computational time is only roughly equal to the sum of the time required for the different components because we omitted the computationally insignificant but necessary step of computing the coefficients in the systems of equations we needed to solve. We observe that in the L-GPA, M-GPA and C-GPA algorithms, a significant fraction of the computational effort is spent on finding the roots of a polynomial. However, in the S-GPA algorithm, mapping costs more than finding a root by Newton’s method. The data also show that the most expensive algorithm was CNA because it required a system of equations to be solved rather than simply finding the roots of a polynomial of degree four as in the case of the algorithms L-GPA, M-GPA, and C-GPA.

Table 9 contains some interesting observations about the efficacy of the iterative solvers underlying some of these algorithms. First of all, it appears that the Lagrange approach leads to the system requiring the smallest number of iterations. On the other hand, the P-GPA uses a golden search algorithm to compute initial estimates of the minima and maxima before invoking Newton’s method to converge rapidly to the minima and maxima. Unfortunately, our tests indicate that it is difficult to reduce the number of iterations in the golden search without obtaining initial estimates for which Newton’s method will not converge. As a matter of fact, even using the golden search algorithm with the recommended tolerances, roughly $1\%$ of the pairs of ellipses did not converge to a contact point for the P-GPA. In order to maintain the consistency of our tests, the pairs of ellipses for which P-GPA failed to converge were also excluded from our tests with the other methods. We remark that the standard deviation of number of iterations for M-GPA and C-GPA are higher than the other algorithms, which leads us to conclude that the number of iterations of M-GPA and C-GPA depend on the relative configuration of the two ellipses. In contrast, the convergence of the S-GPA appears to be independent of the relative geometry between the ellipses.

**Table 10:** Computational cost, number of iterations, and logarithmic error for different algorithms using a relative tolerance of $10^{-9}$

	L-GPA	P-GPA	M-GPA	C-GPA	S-GPA
Total computational cost (s)	12.12	8.26	16.57	15.20	1.96
Mapping	0	0	1.31	1.07	1.31
Initialization (focal points)	0	0	0	0	0.21
Initialization (golden search)	0	5.55	0	0	0
Root finding (Francis’ algorithm)	11.25	0	14.14	13.09	0
Root finding (Newton’s method)	0	0.11	0	0	0.11
Root finding (MATLAB functions)	0	0	0	0	0
Number of iterations					
Maximum	46	201	59	55	6
Minimum	14	15	5	5	2
Median	21	19	26	24	5
Standard deviation	3.37	7.24	8.66	8.53	0.80
Logarithmic error					
Maximum	$-\,9.14$	$-\,9.08$	$-\,9.16$	$-\,9.05$	$-\,9.16$
Median	$-\,14.61$	$-\,15.14$	$-\,14.75$	$-\,15.41$	$-\,15.67$
Standard deviation	1.47	2.01	1.01	0.88	0.84

**Fig. 29:**
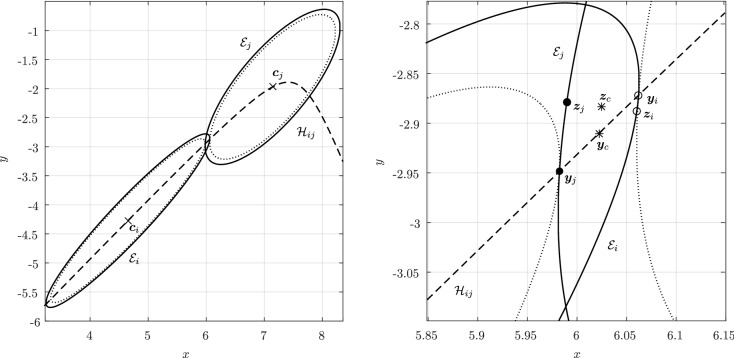
In this configuration of ellipses, M-GPA requires the largest number of iterations to find ${\varvec{y}}_j$. The points $({\varvec{y}}_i,{\varvec{y}}_j,{\varvec{y}}_c)$ are the MPP and the contact point, while $({\varvec{z}}_i,{\varvec{z}}_j,{\varvec{z}}_c)$ are associated with the MDP and its contact point

**Fig. 30:**
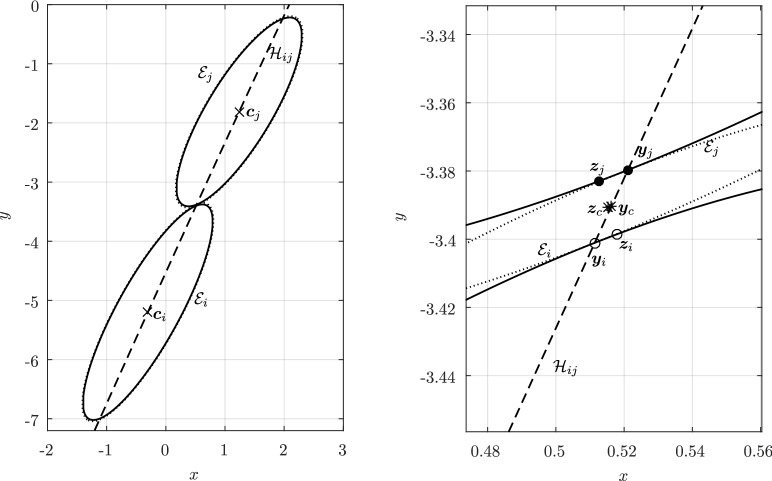
In this configuration of ellipses, L-GPA required its largest number of iterations to find ${\varvec{y}}_i$

**Fig. 31:**
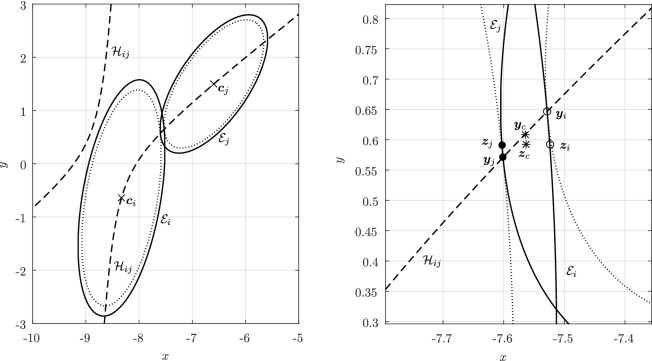
In this configuration of ellipses, C-GPA requires its largest number of iterations to find ${\varvec{y}}_i$

Later in this section, we will examine the pairs of ellipses for which different algorithms attained the maximum number of iterations; see Figs. 29, 30, and 31. Although the number of iterations required by different algorithms is not necessarily correlated, the pairs of ellipses for which a specific algorithm had more difficulty could indicate that certain geometrical properties reduce robustness.

Overall, the data indicate that the S-GPA algorithm was the most efficient. Our hypothesis is that this algorithm combines a good initial estimate of the MPP (for an ellipse at origin and a unit circle) with Newton’s method quadratic convergence. In practice, we found that the S-GPA often converged in a single iteration. Finally, the numerical experiments indicate that the CNA and CCA algorithms were by far the most costly alternatives. We will therefore refrain from discussing them any further.

We now turn to the data in Table 10 obtained using the same set of pairs of ellipses but with a stricter tolerance of $10^{-9}$. A priori, we expect the results to indicate the same overall trends but the stricter tolerance should help to identify any robustness issues. It is clear that the stricter tolerance produces more accurate estimates and requires a larger number of iterations. Yet, it is noticeable that the median number of iterations is almost identical, while the median error is roughly $10^{-4}$ smaller. This indicates that for the majority of the test cases we considered, the algorithm was already within the asymptotic regime of convergence and in many cases one or no iteration was required to satisfy the desired tolerances.

Among the L-GPA, M-GPA, and C-GPA, the L-GPA algorithm is again the most efficient and accurate, but it is still an order of magnitude slower than the S-GPA. The low standard deviation of the number of iterations suggests that the S-GPA was also the most robust algorithm. It appears that the M-GPA and C-GPA algorithms both required more iterations of their root-finding algorithm, Francis’ method, in order to obtain the MPP, particularly when contrasted with L-GPA. We hypothesize that the mapping step, present in M-GPA and C-GPA but not in L-GPA, might make the root-finding problem harder, although further tests would be required to confirm this.

Finally, we conclude this section by examining pairs of ellipses for which certain algorithms required the largest number of iterations from among our sample set. Figure 29 presents the pair of ellipses that required the largest number of iterations in Francis’ method. In this case, the two ellipses appear to have their principal axis roughly aligned and to be in contact near the regions of the highest curvature. We also note that in this configuration, the points on the segment formed by ${\varvec{y}}_i$ and ${\varvec{y}}_j$ cross the segment formed of ${\varvec{z}}_i$ and ${\varvec{z}}_j$. It is somewhat surprising that this configuration might be difficult for the M-GPA to handle. Figure 30 presents the pair of ellipses that required the most iterations of the L-GPA. This configuration appears very similar to the one associated with the M-GPA. Finally, Fig. 31 presents the worst-case scenario for the C-GPA and again the configuration seems unexceptional although the principal axis is angled by roughly $\pi /4$ and the contact occurs at points where the curvature is intermediate. In other words, the pair of ellipses in Fig. 31 is completely the opposite to what we found in the previous two configurations. We did not present the pair of ellipses associated with the worst-case scenario for the S-GPA algorithm because there was very little variation on the number of iterations. Furthermore, the S-GPA algorithm profited from a unique initialization; hence, a comparison with the other GPA-type algorithms would be biased.

## Conclusions and perspectives

This research attempted to review and restructure the problem of detecting and estimating contact detection between pairs of ellipses. The topic has been studied by numerous engineers with most of the algorithms resting on heuristics, but as a result, much of the mathematical theory had yet to be fleshed out. This research has filled some of the mathematical and numerical gaps, particularly in 2D, and this has led the authors to reclassify published algorithms in order to highlight their differences and similarities. Furthermore, the authors have proposed a methodology to compare fairly the different contact detection algorithms (CDAs), something that was lacking in the literature. Finally, the authors demonstrated the usefulness of their analysis by introducing a novel CDA combining old and new techniques.

Although this research has identified and characterized several concepts that were implicit or ignored in past research, it is the authors hope that this work will help to stimulate research on this topic. Much of the mathematical theory still needs to be extended to 3D, particularly the co-gradient locus, and a proper study may provide some new insight into computational aspects of the subject. A careful analysis of the mathematical theory indicates that the main tools are the convexity of the ellipses and scaling arguments in projective space, which suggests that the notion of the co-gradient locus, and many of its associated concepts, could be extended to other families of convex particles. There are numerous mechanical, and even esoteric, procedures to construct ellipses, some of which may lead to original CDAs. This research only briefly mentioned connections to the Perram–Wertheim contact theory, or to the time-dependent contact detection problem, and connections to both of these theories could be fruitful. On the other hand, there exists a large literature on contact potentials [16, 39] that may be very instructive to designing better CDAs, if not just better initial approximations for contact points. Our research has identified three notions of contact that could be used in collision detection, and although we believe these may be the only ones possible, there may exist others.

This paper has discussed only the narrow phase of contact detection, but reductions in the computational cost of the broad-phase search could have significant impacts on DEM/MD simulations. It seems reasonable to assume that machine learning techniques could lead to breakthrough on this problem.

The numerical methodology for the comparison of CDAs could be pursued even further to obtain more precise information about accuracy and robustness. Although the notions of contact all coincide, it is not clear whether or not these notions might introduce long-term biases in large-scale particle simulations. On the other hand, the potential for bias is more likely in approximations of particles by clusters of spheres, and even that issue has not been explored. Finally, there is still a need for more test problems, such as those of Sects. 6.1–6.4, and we encourage other researchers to continue to share challenging configurations of ellipses.

## Footnotes

1 In the introduction, ellipses will be used to refer to both ellipses in 2-D and ellipsoids in 3-D.

2 Note that we have chosen to follow the usual notation ${\mathcal {E}}_i$ and ${\mathcal {E}}_j$ for an arbitrary pair of ellipses in order to be consistent with the notation most frequently encountered in the literature.
